# Quick assessment for systematic test statistic inflation/deflation due to null model misspecifications in genome-wide environment interaction studies

**DOI:** 10.1371/journal.pone.0219825

**Published:** 2019-07-18

**Authors:** Masao Ueki, Masahiro Fujii, Gen Tamiya, for Alzheimer’s Disease Neuroimaging Initiative and the Alzheimer’s Disease Metabolomics Consortium

**Affiliations:** 1 Statistical Genetics Team, RIKEN Center for Advanced Intelligence Project, Chuo-Ku, Tokyo, Japan; 2 Graduate School of Medicine, Kurume University, Kurume, Fukuoka, Japan; 3 Tohoku Medical Megabank Organization, Tohoku University, Aoba-Ku, Sendai, Japan; University Hospital Jena, GERMANY

## Abstract

Gene-environment (GxE) interaction is one potential explanation for the missing heritability problem. A popular approach to genome-wide environment interaction studies (GWEIS) is based on regression models involving interactions between genetic variants and environment variables. Unfortunately, GWEIS encounters systematically inflated (or deflated) test statistics more frequently than a marginal association study. The problematic behavior may occur due to poor specification of the null model (i.e. the model without genetic effect) in GWEIS. Improved null model specification may resolve the problem, but the investigation requires many time-consuming analyses of genome-wide scans, e.g. by trying out several transformations of the phenotype. It is therefore helpful if we can predict such problematic behavior beforehand. We present a simple closed-form formula to assess problematic behavior of GWEIS under the null hypothesis of no genetic effects. It requires only phenotype, environment variables, and covariates, enabling quick identification of systematic test statistic inflation or deflation. Applied to real data from the Alzheimer’s Disease Neuroimaging Initiative (ADNI), our formula identified problematic studies from among hundreds GWEIS considering each metabolite as the environment variable in GxE interaction. Our formula is useful to quickly identify problematic GWEIS without requiring a genome-wide scan.

## Introduction

Gene-environment (GxE) interaction is one potential way to unravel the missing heritability problem [1–3]. Genome-wide GxE interaction studies are becoming popular, as evidenced by recent reviews [4–9]. In large-scale genomic analyses, such as genome-wide association studies (GWAS) and genome-wide environment interaction studies (GWEIS), hypothesis tests based on regression models are widely used to discover genetic susceptibility variants. Each genetic variant is marginally examined with a univariate regression model in a GWAS, and the analysis may be adjusted for covariates—such as sex and age. GWEIS is similarly conducted in a regression model that involves GxE interaction. Due to low power for testing interactions between genetic variants and environment variables (statistical interaction), however, a joint test for the presence of genetic effects allowing GxE interaction [10] is frequently used [11–14]. Its simplicity is an advantage over other existing methods, and we focus on the joint test throughout this paper. With GWAS data, up to a million genetic variants can be tested, and the multiplicity of hypotheses can be accounted for. For valid discovery of genetic susceptibility variants, it is necessary that the type I error is properly controlled. Systematic inflation or deflation in test statistics over all loci means that the study is problematic; population stratification and cryptic relatedness are typical reasons. A poor specification of null model (i.e. the model which includes no genetic effect but may include covariate/environment variables) may also cause problematic behavior of the test statistics unexpected under the null hypothesis, because then the null hypothesis is false for all loci regardless of the presence of genetic effects. Problematic behavior is observed in GWEIS more frequently than in GWAS [15, 16].

The severity of problematic behavior can be quantified by the median or mean of the chi-squared statistics for association from a genome-wide scan. For example, the genomic inflation factor constant λ is estimated by the median [17] or mean [18, 19] of genome-wide chi-squared statistics. Currently, numerous feature variables tend to be collected from a large number of participants in cohort or biobank studies [20]. Researchers often have many candidate variables that can be used as covariates and environment variables in GxE interaction analyses. Severe systematic inflation (or deflation) in genome-wide test statistics indicates that the study is problematic. However, the computational load is high when one needs numerous genome-wide scans with large samples, as in recent biobank studies, in which a large number of candidate environment variables needs to be evaluated. Moreover, with genome-wide imputed data [21, 22] or whole-genome sequencing data, a larger number of variants is tested for association than with SNPs (single nucleotide polymorphisms) in GWAS. It would therefore be helpful if problematic behavior could be found before conducting a computationally expensive genome-wide scan.

By assuming no genetic effect, we present a novel closed-form approximation to the mean of the genome-wide joint GxE interaction test statistics, which can be used to assess problematic behavior due to null model misspecification. The formula disregards characteristics of genetic loci, thereby enabling preliminary use before computing chi-squared statistics by genome-wide scan. We show that the approximation agrees well with the mean of the chi-squared statistics from various joint GxE interaction tests for GWAS data from the ADNI. Our approximation is also valid for marginal association tests. The approximation formula reveals that the joint GxE interaction test is sensitive to null model misspecification, whereas the marginal association test is not.

The remainder of the paper is organized as follows. In the Materials and Methods section, we provide the proposed approximation formula, describe the real data application using SNP-GWAS data from ADNI for joint GxE interaction and marginal association analyses, and provide the setup of simulation studies to evaluate the performance of the approximation. In the Results section, we give the results from the real data application and simulation studies. In the Discussion section, we summarize the results and discuss about the proposed approach both theoretically and empirically.

## Materials and methods

### The approximation formula

Suppose that *n* samples are observed with phenotypic value (binary, numeric value, or a factor) denoted by *y*_1_, …, *y*_*n*_, and *L* genetic variants, **g**_*l*_ = (*g*_*l*,1_, …, *g*_*l*,*n*_)^*T*^ for *l* = 1, …, *L*, are to be tested for association with the phenotype. We introduce *p* variables, $w_{l,i}^{T}=\left(w_{l,i1},\ldots ,w_{l,ip}\right)$ for sample *i* (*i* = 1, …, *n*) at the *l*th locus to be tested for association (i.e. *H*_0*l*_), such as **g**_*l*_ itself or an interaction between **g**_*l*_ and an environment variable. Let $z_{i}^{T}=\left(z_{i1},\ldots ,z_{iq}\right)$ denote *q* covariates (e.g. sex or age) of sample *i* to be adjusted in common for all *L* tests. We consider *L* hypothesis tests of the null hypothesis *H*_0*l*_: *β*_*l*_ = **0** under the following regression model for the conditional mean of *y*_*i*_ with transformation,

$$\eta_{i}=\eta \left\{E\left(y_{i}|w_{l,i}^{T},z_{i}^{T}\right)\right\}=w_{l,i}^{T}\beta_{l}+z_{i}^{T}\gamma_{l},$$ (1)

for *i* = 1, …, *n*, where *η* is a monotone increasing function, and $\beta_{l}^{T}=\left(\beta_{l,1},\ldots ,\beta_{l,p}\right)$ and $\gamma_{l}^{T}=\left(\gamma_{l,1},\ldots ,\gamma_{l,q}\right)$ are the regression coefficients. The above model reduces to the ordinary linear regression model if *η* is the identity function and *y*_*i*_ follows a Gaussian distribution. The model reduces to the logistic regression model if *η* is the logit function and *y*_*i*_ follows a Bernoulli distribution. The model under *H*_0*l*_ (i.e. $\eta_{i}=z_{i}^{T}\gamma$) is referred to as the null model throughout the paper.

In what follows, we show that the above test includes the joint GxE interaction test as well as the marginal association test. Let **x**_*i*_ for the *i*th sample be an environment variable to be tested for interaction with *g*_*l*,*i*_. Here we allow multivariate environment variables for **x**_*i*_. Then, if **w**_*l*,*i*_ = *g*_*l*,*i*_**x**_*i*_, the above test turns out to be the joint GxE interaction test of Kraft et al. [10] with **x**_*i*_ as environment variables, in which the first element is one for all *i*. If **x**_*i*_ = 1 for all *i* and *p* = 1, the test reduces to the marginal association test.

We study the chi-squared statistic for the score test of the above regression model (1). According to [23], the test statistic for testing *H*_0*l*_: *β*_*l*_ = **0** can be expressed as follows:

$$t_{l}=u^{T}\left(Q_{\tilde{Z}}{\tilde{W}}_{l}\right){\left({\tilde{W}}_{l}^{T}Q_{\tilde{Z}}{\tilde{W}}_{l}\right)}^{-1}{\left(Q_{\tilde{Z}}{\tilde{W}}_{l}\right)}^{T}u,$$ (2)

where $Q_{\tilde{Z}}=I-P_{\tilde{Z}}$, $P_{\tilde{Z}}=\tilde{Z}{\left({\tilde{Z}}^{T}\tilde{Z}\right)}^{-1}{\tilde{Z}}^{T}$, ${\tilde{W}}_{l}=\Omega^{1/2}W_{l}$, $\tilde{Z}=\Omega^{1/2}Z$, Ω = *diag*(*ω*_1_, …, *ω*_*n*_), $W_{l}^{T}=\left(w_{l,1}^{T},\ldots ,w_{l,n}^{T}\right)$, $Z^{T}=\left(z_{1}^{T},\ldots ,z_{n}^{T}\right)$, the *ω*_*i*_s are positive values specific to the regression model, and **u**^*T*^ = (*u*_1_, …, *u*_*n*_) depends on *y*_1_, …, *y*_*n*_. The above *t*_*l*_ in (2) is just another representation of the standard score test statistic, and hence, the null distribution is asymptotically chi-squared with *p* degrees of freedom (*p*df).

For example, *t*_*l*_ reduces to the score statistic for a logistic regression model by setting $u_{i}=\left(y_{i}-{\hat{\mu }}_{i}\right)/{\sqrt{\omega }}_{i}$ with $\omega_{i}={\hat{\mu }}_{i}\left(1-{\hat{\mu }}_{i}\right)$, ${\hat{\mu }}_{i}=1/\left\{1+\text{exp}\left(-z_{i}^{T}\hat{\gamma }\right)\right\}$, and $\hat{\gamma }$ is the maximum likelihood estimator under the null hypothesis *β*_*l*_ = **0**. More generally, *t*_*l*_ reduces to the score statistic for a more general regression model having loglikelihood function *ℓ* = *ℓ*(*η*) by setting $u_{i}=\left(\partial /\partial \eta_{i}\right)\ell /{\sqrt{\omega }}_{i}$ with *ω*_*i*_ = −(∂^2^/∂^2^*η*_*i*_)*ℓ* evaluated at the null hypothesis *β*_*l*_ = **0**. Furthermore, if *ω*_*i*_ = 1 for all *i*, $\hat{\gamma }={\left(Z^{T}Z\right)}^{-1}Z^{T}y$, and **u** = **y**, then

$$T_{l}=\frac{t_{l}}{(∥Q_{Z}y{∥}^{2}-t_{l})/n}$$ (3)

is approximately the t-test statistic in a Gaussian linear regression model. For the above representation of the score test statistic, see [23] for mathematical details.

We specifically provide the form of (2) for joint GxE interaction test. By defining $\tilde{X}=\Omega^{1/2}X$ where $X^{T}=\left(x_{1}^{T},\ldots ,x_{n}^{T}\right)$, the relation **w**_*l*,*i*_ = *g*_*l*,*i*_**x**_*i*_ can be written in matrix form as **W**_*l*_ = **G**_*l*_**X**, and also as ${\tilde{W}}_{l}=G_{l}\tilde{X}$, where **G**_*l*_ = *diag*(*g*_*l*,1_, …, *g*_*l*,*n*_). Then, (2) is expressed as

$$t_{l}=u^{T}\left\{Q_{\tilde{Z}}\left(G_{l}\tilde{X}\right)\right\}{\left\{{\left(G_{l}\tilde{X}\right)}^{T}Q_{\tilde{Z}}\left(G_{l}\tilde{X}\right)\right\}}^{-1}{\left\{Q_{\tilde{Z}}\left(G_{l}\tilde{X}\right)\right\}}^{T}u.$$ (4)

The Kraft’s 2df test is obtained if **x**_*i*_ = (1, *E*_*i*_) with an environment variable *E*_*i*_ considered for GxE interaction. For marginal association test, letting ${\tilde{g}}_{l}=\Omega^{1/2}g_{l}$, (2) is given by

$$t_{l}={\left\{{\left(Q_{\tilde{Z}}{\tilde{g}}_{l}\right)}^{T}u\right\}}^{2}/{\tilde{g}}_{l}^{T}Q_{\tilde{Z}}{\tilde{g}}_{l}.$$ (5)

Systematic inflation (or deflation) of test statistics can be quantified by the overall behavior of genome-wide test statistics. Under the presence of population stratification, empirical distribution from test statistics is inflated from $\chi_{1}^{2}$ to $\lambda \chi_{1}^{2}$ [24]. This inflation factor λ can be estimated from test statistics, *t*_1_, …, *t*_*L*_. Two estimators for λ are the median of *t*_1_, …, *t*_*L*_ divided by the theoretical median of $\chi_{1}^{2}$ distribution [17] and the mean [18]. Deviation of the estimated λ from one suggests that test statistics are problematic, e.g. due to the presence of population stratification or cryptic relatedness.

We study the mean of genome-wide test statistics analytically. Specifically, we approximate the expectation of the mean of test statistics for the *L* loci, $t_{mean}=\frac{1}{L}\sum_{l=1}^{L}t_{l}$,

$$E_{g_{1},\ldots ,g_{L}}\left(t_{mean}\right)=\frac{1}{L}\sum_{l=1}^{L}E_{g_{l}}(t_{l})$$ (6)

by

$$\frac{1}{L}\sum_{l=1}^{L}\text{tr}[{\left\{E_{g_{l}}\left({\tilde{W}}_{l}^{T}Q_{\tilde{Z}}{\tilde{W}}_{l}\right)\right\}}^{-1}E_{g_{l}}\left\{{\left(Q_{\tilde{Z}}{\tilde{W}}_{l}\right)}^{T}uu^{T}\left(Q_{\tilde{Z}}{\tilde{W}}_{l}\right)\right\}],$$

where $E_{g_{1},\ldots ,g_{L}}$ and $E_{g_{l}}$ denote the expectations with respect to the joint distribution of **g**_1_, …, **g**_*L*_ and to the marginal distribution of **g**_*l*_, respectively. If *q* = 1, the matrix inverse is just the reciprocal. Hence, the proposed approximation is a multi-dimensional extension of the approximation of the mean of a ratio by the ratio of means. In order to derive the above approximation, we impose the following assumption for each tested variant independently: *g*_*l*,1_, …, *g*_*l*,*n*_ are independently and identically distributed whose all moments are finite, with mean and variance denoted by *μ*_*l*_ and $\sigma_{l}^{2}$, respectively (e.g. with a binomial distribution of size 2 and success probability being the minor allele frequency (MAF), which is the law under Hardy–Weinberg equilibrium (HWE)). We also assume that the **g**_*l*_ are independent of **u**, **Z**, and **X**. Since the approximation applies separately for each *l*, each variant may have a different genotype distribution (i.e. different MAF), and variant frequencies may be correlated due to linkage disequilibrium. The above assumptions exclude the case where the tested variant **g**_*l*_ itself causes systematically inflated test statistics, e.g., due to population stratification, cryptic relatedness, sample difference of genotyping efficiency, or a batch effect. In addition, we assume that *p* ≤ *q* and **Z** contains **X**. Without loss of generality, the first *p* columns of **Z** correspond to **X**, or **Z** = (**Z**_1:*p*_, **Z**_(*p*+1):*q*_) = (**X**, **Z**_(*p*+1):*q*_), where **Z**_1:*p*_ represents the first *p* columns of **Z** and **Z**_(*p*+1):*q*_ are the remaining columns. Now we study the expectation of *t*_*l*_ with respect to **g**_*l*_. Our approximation formula for the expectation of *t*_*l*_ with respect to **g**_*l*_ is:

$$\begin{array}{ccc} E_{g_{l}}\left(t_{l}\right) & \approx & \text{tr}[{\left\{E_{g_{l}}\left({\tilde{W}}_{l}^{T}Q_{\tilde{Z}}{\tilde{W}}_{l}\right)\right\}}^{-1}E_{g_{l}}\left\{{\left(Q_{\tilde{Z}}{\tilde{W}}_{l}\right)}^{T}uu^{T}\left(Q_{\tilde{Z}}{\tilde{W}}_{l}\right)\right\}]\\ & = & \text{tr}[\{\sum_{i=1}^{n}{\tilde{x}}_{i}{\tilde{x}}_{i}^{T}{\left(Q_{\tilde{Z}}\right)}_{ii}{\}}^{-1}\{\sum_{i=1}^{n}{\tilde{x}}_{i}{\tilde{x}}_{i}^{T}{\left(Q_{\tilde{Z}}u\right)}_{i}^{2}\}]\\ & = & t_{approx}, \end{array}$$ (7)

where the approximation holds by ignoring *O*(*n*^−1^) terms. The derivation is given in S1 Appendix, in which the proof is based on an induction and asymptotic expansion. In the above formula, ${\left(Q_{\tilde{Z}}\right)}_{ii}$ is 1 minus the leverage score of the *i*th datum, $1-{\left(P_{\tilde{Z}}\right)}_{ii}$, while ${\left(Q_{\tilde{Z}}u\right)}_{i}$ is the *i*th residual from a regression of **u** on $\tilde{Z}$. Notably, the formula (7) no longer contains characteristics of **g**_*l*_. As a result, (6) is approximated by (7). The formula (7) can be used to investigate the overall behavior of *t*_*l*_ without requiring a genome-wide scan. In S1 Appendix, we show that, if the null model is correct, the above *t*_*approx*_ is close to *p*, the expected value of *t*_*mean*_. A large difference between *t*_*approx*_ and *p* indicates problematic null model specification because we assume that the **g**_*l*_s do not cause a problem. Analogous to the genomic inflation factor, we consider the scaled version of *t*_*approx*_,

$$l_{approx}=t_{approx}/p,$$

and, similarly, *l*_*mean*_ = *t*_*mean*_/*p*. The approximation formula for the Gaussian linear regression model (3) is

$$T_{approx}=\frac{t_{approx}}{(∥Q_{Z}y{∥}^{2}-t_{approx})/n}.$$ (8)

Similarly, the scaled versions are *l*_*approx*_ = *T*_*approx*_/*p* and *l*_*mean*_ = *T*_*mean*_/*p*, in which $T_{mean}=\frac{1}{L}\sum_{l=1}^{L}T_{l}$.

The case where *l*_*approx*_ is close to one suggests that the null model is appropriate (or at least has no problematic behavior), and it is expected that the test statistics behave properly unless genetic variants cause problems in the test statistics. Checking systematic inflation or deflation by marginal association scan allows to check whether genetic variants cause problems. On the other hand, a large discrepancy from one suggests null model misspecification, in which case systematically inflated test statistics will be observed after genome-wide scan and the test is unreliable.

### Real data application

We illustrate the validity and usefulness of our approximation through application to a real GWAS dataset obtained from the publicly available Alzheimer’s Disease Neuroimaging Initiative (ADNI) database (adni.loni.usc.edu). The ADNI was launched in 2003 as a public-private partnership, led by Principal Investigator Michael W. Weiner, MD. The primary goal of ADNI has been to test whether serial magnetic resonance imaging (MRI), positron emission tomography (PET), other biological markers, and clinical and neuropsychological assessments can be combined to measure the progression of mild cognitive impairment and early Alzheimer’s disease. For up-to-date information, see www.adni-info.org. ADNI is an ongoing, longitudinal study with primary purpose being to explore the genetic and neuroimaging information associated with late-onset Alzheimer’s disease (LOAD). The study investigators recruited elderly subjects older than 65 years. The cohort comprised about 400 subjects with mild cognitive impairment, about 200 subjects with Alzheimer’s disease, and about 200 healthy controls. Each subject was followed for at least 3 years. During the study period, the subjects were assessed with magnetic resonance imaging (MRI) measures and psychiatric evaluation to determine cognitive status at each time point. Study subjects gave written informed consent at the time of enrollment for imaging and genetic sample collection and completed questionnaires approved by Institutional Review Board (IRB) of each participating sites (http://adni.loni.usc.edu/wp-content/uploads/how_to_apply/ADNI_Acknowledgement_List.pdf). We obtained approval from the ADNI Data Sharing and Publications Committee for use of the data and analyzed the data anonymously.

The ADNI-GWAS data were obtained from 818 DNA samples of ADNI1 participants using the Illumina Human 610-Quad genotyping array [25]. The genotype data we used is in PLINK format available from ADNI website (http://adni.loni.usc.edu/) which includes 620,901 SNPs for 757 individuals. We applied a quality control procedure by excluding SNPs with missing genotype rate > 0.05, HWE test *P* < 10^−10^, and MAF < 5%; the total number of remaining SNPs was 521,203.

The dataset with 757 samples is comprised of multiple ethnic groups. We computed principal components (PCs) using the EIGENSOFT package [26, 27]. The first and second PCs are given in S1 Fig, which indicates the presence of population stratification. Our approximation imposes a stringent assumption that all samples follow the same distribution for each locus. It does not cover the case where population stratification exists, and the approximation is not guaranteed under the presence of population stratification due to association between phenotype and genotypes. To see the performance on data without population stratification, we created another dataset by extracting 684 non-Hispanic Caucasian samples from 757 samples after excluding one individual from pairs showing cryptic relatedness (revealed by the PLINK [28] pairwise $\hat{\pi }$ statistic being greater than 0.125), and we excluded subjects whose reported sex did not match the sex inferred from X chromosome SNPs. We used two datasets, one with the 684 samples and another with the 757 samples. Since population stratification is absent in the former dataset, it is expected that *l*_*approx*_ well approximates *l*_*mean*_, while it is not guaranteed that the approximation is well for the latter dataset. To make the approximation workable, we considered adjusting for PCs as covariates [26]. Although an appropriate number of PCs for adjustment depends on the population structure and the sample size, we included top 10 PCs, which is the number generally accepted within the psychiatric genetics community [29]. We also considered top 3 and 5 PCs for covariates in order to evaluate the influence of the number of PCs for adjustment.

To check the accuracy of our approximation, we compared *l*_*approx*_ with *l*_*mean*_ computed from the genome-wide test statistics from the joint GxE interaction and marginal association analyses. We obtained environment variables, phenotypes, and covariates from the R package ADNIMERGE provided by ADNI. We chose five phenotypes: height (HEIGHT), body mass index (BMI), whole brain volume (WholeBrain), intracranial volume (ICV), and mini mental state examination (MMSE), which are all quantitative. For environment variables in GxE interaction analyses, we used 142 metabolite variables stored in the admcdukep180fia object in ADNIMERGE gathered by the Alzheimer’s disease Metabolomics Consortium. By setting values coded as “< LOD” as missing, and excluding metabolites showing missing rate greater than 20%, we had 117 metabolites for analysis. Because conducting genome-wide scans for all 117 variables is time-consuming, we used only nine variables—lysoPC.a.C16.0, PC.ae.C38.2, PC.ae.C40.3, C10, PC.aa.C40.5, PC.ae.C36.3, SM‥OH‥C14.1, SM‥OH‥C22.1, and SM.C24.0—as environment variables for genome-wide joint GxE interaction analyses using linear models for the five phenotypes. Among the nine variables, two variables were chosen based on application of the proposed approximation formula to the joint GxE interaction test with each of the five phenotypes and each of 117 metabolite variables as the environment variable. First, we computed *l*_*approx*_ for joint GxE interaction test with respect to the five phenotypes and 117 metabolites by adjusting for sex and age. We stored the results in S1 Table. The scatter plots of the phenotype-environment pairs showing *l*_*approx*_ > 1.5 are given in S2 Fig. There seemed to be roughly two groups: the first group exhibited quadratic relationship between phenotype and environment variable rather than linear, and the second group included seeming outliers. Therefore, the large discrepancy of *l*_*approx*_ from one was caused by the null model specification. For the first group, we randomly chose the BMI—PC.ae.C38.2 pair. For the second group, we randomly chose the BMI—PC.ae.C40.3 pair. The remaining seven metabolites were chosen randomly.

In the analyses for the nine metabolites and five phenotypes, we attempted to automatically resolve the systematic inflation by using a Box–Cox transformation [30] of the phenotype (actually, the transformation was applied to the phenotype subtracted the minimum value and added 1 to make the values positive). First, we applied the standard Box–Cox transformation based on normality (i.e. making the transformed phenotype distribution close to normal) using the “boxCox” function implemented in the car package in R. Next, we optimized the Box–Cox transformation in terms of the closeness of *l*_*approx*_ to one, which addresses the systematic inflation issue directly.

Our proposed approximation can also be applied to the score test for generalized linear models. We dichotomized the five quantitative phenotypes by whether the phenotype value is greater than its mean, and applied the score test for a logistic regression model to joint GxE interaction and marginal association tests.

We considered the impact on the approximation when the real genotype data is replaced by simulated genotype data. We repeated the same analyses for the nine metabolites and five phenotypes on the ADNI 684 samples described above with the simulated genotype data rather than with the real genotype data, while phenotype, environment, and covariates were fixed. In the above simulation procedure, 10,000 unlinked loci were simulated using PLINK --simulate option, where MAFs were randomly generated from a uniform distribution in [0.05,0.5]. We also repeated the above analyses using the artificial genotype data for the ADNI data with the 757 samples.

To see the impact of null model specification, we investigated the BMI—PC.ae.C38.2 and BMI—PC.ae.C40.3 pairs in detail. We considered sophisticating null modeling by applying a quadratic model rather than the linear model or removing outliers. Voorman et al. [15], Tchetgen Tchetgen and Kraft [31] and Almli et al. [16] proposed a robust test using the Huber–White robust variance for the GxE interaction test to account for null model misspecification. We applied the robust joint GxE interaction test using the Almli’s robust joint interaction program available from “http://genetics.emory.edu/labs/epstein/software/robust-joint-interaction/”. Another solution to the systematic inflation of test statistics is to use the genomic control-adjusted p-values, i.e. chi-squared test statistics divided by a constant so that the median matches the expected value of one. We applied the genomic control-adjustment for the two examples.

### Simulation studies

To study the behavior of *l*_*approx*_ for joint GxE interaction and marginal association tests, we carried out extensive simulation experiments. Simulation scenarios are described in Table 1. The aims of each scenario are as follows.

- Baseline scenario. This is a baseline for comparison. Other scenarios are a slight modification of the baseline scenario.
- Association among environment, covariate variables and/or genotypes (Scenarios 1a, 1b, 1c, and 1d). The aim is to assess the influence on the proposed approximation of association among environment, covariate variables and/or genotypes. Scenarios 1a, 1b, and 1c consider the association between covariate/environment variables and genotypes, which may arise due to population stratification or genetic architecture. Scenario 1d considers the association between environment and covariate variables.
- Misspecified null model (Scenarios 2a, 2b, 2c, 2d, and 2e). The aim is to assess the impact of the misspecified null model on the approximation. It is expected that the misspecification deviates the value of *l*_*mean*_ from one. Scenarios 2a and 2b consider that the null model misses the covariate associated with genotypes, for example, adjustment for population stratification is not applied or inadequate. Scenario 2c considers that the null model misspecifies the functional form of the environment variable. Scenarios 2d and 2e consider the presence of outlier(s).
- Environment/covariate variable distribution (Scenarios 3a, 3b, 3c, and 3d). This scenario is intended to investigate the approximation performance under several kinds of environment and covariate variables. We consider four scenarios, considering continous (e.g. age), binary categorical (e.g. sex), and ordinal categorical (e.g. questionnaire score) variables.
- Genotype distribution (Scenarios 4a, 4b, and 4c). This scenario is intended to evaluate the impact of the distribution of the genetic variants. Scenario 4a considers the correlation between genetic variants due to linkage disequilibrium, while scenarios 4b and 4c consider the different allele frequency spectrum.

**Table 1:** Description of simulation scenarios.

Scenario	Description
Baseline scenario	
Base	No association among environment, covariate variable and genotypes
	Correctly specified null model
	One covariate and environment variables are normally distributed (continuous)
	Genotypes are in linkage equilibrium with uniformly distributed minor allele frequencies
Association among environment, covariate variables and/or genotypes	
1a	Genotypes are associated with covariate
1b	Genotypes are associated with environment variable
1c	Genotypes are associated with covariate and environment variables
1d	Environment variable is associated with covariate.
Misspecified null model	
2a	Covariate associated with genotypes is missed
2b	Covariate associated with genotypes and environment variable is missed
2c	Linear null model is incorrectly specified
2d	One outlier is included
2e	Ten outliers are included
Environment/covariate variable distribution	
3a	Five covariates and environment variables are normally distributed (continuous)
3b	One covariate and environment variables are uniformly distributed (continuous)
3c	One covariate and environment variables are binary variable (binary categorical)
3d	One covariate and environment variables are ordinal variable (ordinal categorical)
Genotype distribution	
4a	Genotypes are in linkage disequilibrium with uniformly distributed minor allele frequencies
4b	Genotypes are in linkage equilibrium with Beta distributed minor allele frequencies
4c	Genotypes are in linkage disequilibrium with Beta distributed minor allele frequencies

We considered four effect size scenarios. Let *b*_*G*_, *b*_*Z*_, and *b*_*GE*_ denote the parameters of genotype, covariate and GxE interaction effects on the phenotype. Then, the four scenarios are given as triplets (*b*_*G*_, *b*_*Z*_, *b*_*GE*_):(0, 0, 0) (no effect of genotype, covariates and GxE interaction), (1, 0, 0) (genotype effect, and no covariates and GxE interaction effects), (0, 1, 0) (covariate effect, and no genotype and GxE interaction effects) and (0, 0, 1) (GxE interaction effect, and no genotype and covariate effects). For the scenarios under the presence of any genotypic effect, (*b*_*G*_, *b*_*Z*_, *b*_*GE*_) = (1, 0, 0) and (0, 0, 1), we considered three genotype codings, additive, recessive, and dominant. We repeated the simulations 200 times to compare *l*_*approx*_ with *l*_*mean*_. Two sample sizes, *n* = 1000 and 10000, were considered. To evaluate the discrepancy between *l*_*approx*_ and *l*_*mean*_, we summarized mean and standard deviation in the 200 simulation runs. In some scenarios, the magnitude of *l*_*mean*_ varied with sample size. Thus, we considered the ratio *l*_*approx*_/*l*_*mean*_, which is useful to quantify how well *l*_*approx*_ approximated *l*_*mean*_ while excluding the impact of the sample size. S1 Appendix describes the technical details of simulation studies. The program code is provided in S2 Appendix.

## Results

### Real data application: Comparison between *l*_*approx*_ and *l*_*mean*_

**Fig 1:**
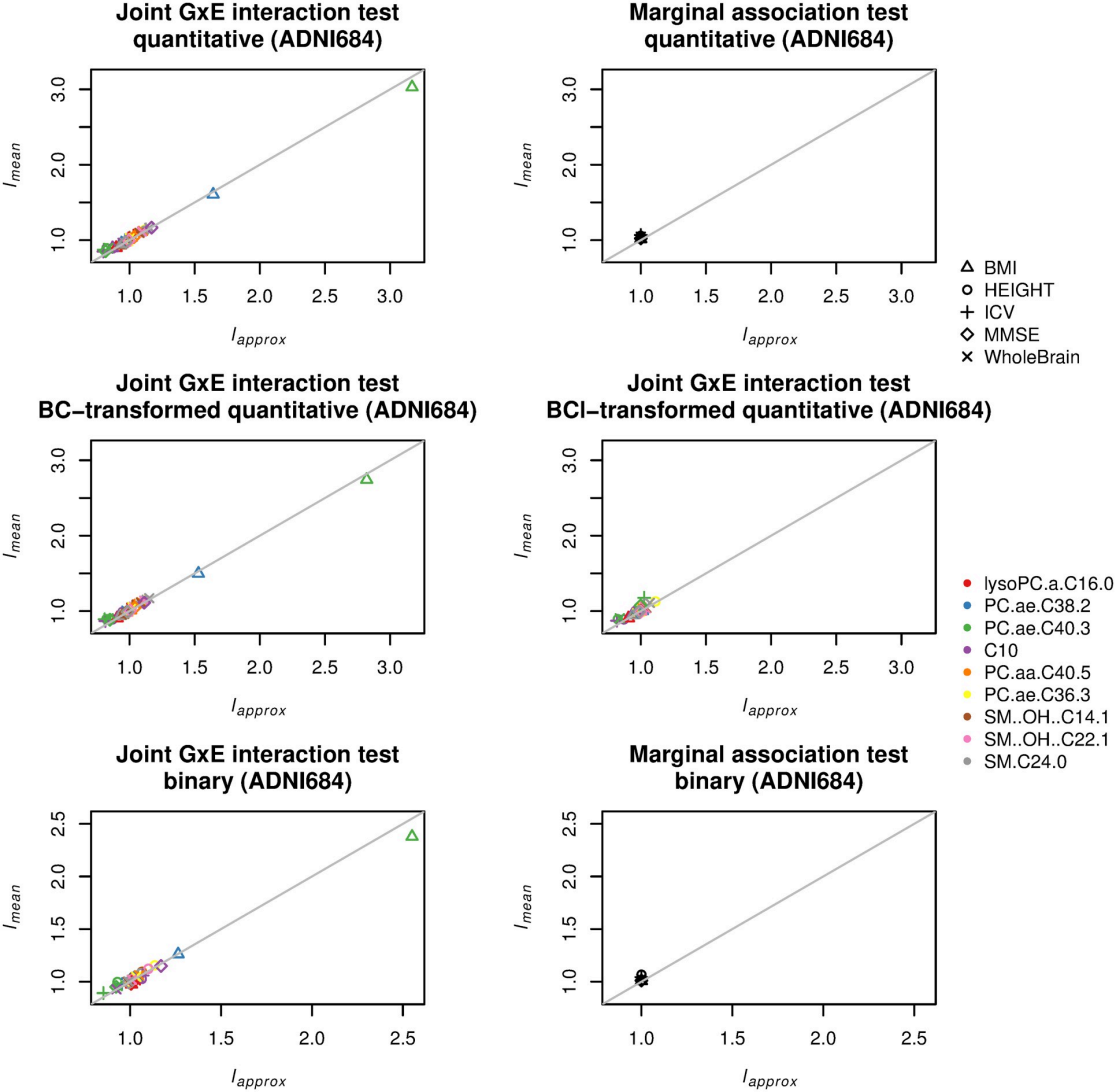
Comparison between *l*_*approx*_ and *l*_*mean*_ for joint GxE interaction and marginal association tests on ADNI-GWAS data for 684 non-Hispanic Caucasian samples (ADNI684). Five phenotypes—height (HEIGHT), body mass index (BMI), whole brain (WholeBrain), intracranial volume (ICV), and mini mental state exam (MMSE)—and nine environment variables: lysoPC.a.C16.0, PC.ae.C38.2, PC.ae.C40.3, C10, PC.aa.C40.5, PC.ae.C36.3, SM‥OH‥C14.1, SM‥OH‥C22.1, and SM.C24.0. Top left: joint GxE interaction test (quantitative phenotype). Top right: marginal association test (quantitative phenotype). Middle left: joint GxE interaction test (quantitative phenotype after Box–Cox transformation optimized in terms of normality). Middle right: joint GxE interaction test (quantitative phenotype after Box–Cox transformation optimized in terms of *l*_*approx*_). Bottom left: joint GxE interaction test (binary phenotype created by dichotomizing quantitative phenotype). Bottom right: marginal association test (binary phenotype created by dichotomizing quantitative phenotype).

Fig 1 gives comparisons between *l*_*mean*_ and *l*_*approx*_ for joint GxE interaction and marginal association tests for each of five quantitative phenotypes and nine metabolites set as an environment variable on the ADNI dataset with 684 non-Hispanic Caucasian samples showing no population stratification. The top left and right panels in Fig 1 give the results from joint GxE interaction and marginal association tests, respectively, which show that *l*_*approx*_ approximated *l*_*mean*_ well as seen by that all points were gathered around the diagonal line. In the top left panel, there were cases where *l*_*mean*_ showed a large discrepancy from one, and two of them are further investigated below. On the other hand, all points in the top right panel were concentrated around one, that is, the means of the chi-squared test statistics from marginal association test were all made closer to one. This behavior differs from that of the joint GxE interaction test, in which the target environment variable **x**_*i*_ plays an important role in determining the test statistic distribution, and dependence of *l*_*approx*_ on **x**_*i*_ cannot be ignored. In S1 Appendix, we show that *l*_*approx*_ is close to one if *n* → ∞ when **x**_*i*_ = 1 for all *i* with *p* = 1 (i.e. the model is reduced to the model for the marginal association test and the corresponding null distribution is 1df chi-squared), which in turn implies that the mean of the chi-squared statistics is approximately one irrespective of what the null model is used.

The case with large *l*_*mean*_ observed in the top left panel means the presence of systematic inflation in genome-wide test statistics. Middle left panel of Fig 1 gives the comparison between *l*_*approx*_ and *l*_*mean*_ for the phenotypes with Box–Cox transformation based on normality. However, systematic inflation still appeared. On the other hand, the Box–Cox transformation optimized in terms of the closeness of *l*_*approx*_ mitigated the systematic inflation as seen in the middle right panel of Fig 1.

The bottom left and right panels in Fig 1 give the results for binary phenotype, created by dichotomizing quantitative phenotype, from joint GxE interaction and marginal association tests for quantitative phenotypes, respectively. The approximation looks well for the 684 non-Hispanic Caucasian samples, exhibiting a similar tendency of the results for quantitative phenotypes. Note that *l*_*approx*_ will not always be close to one for models other than Gaussian unless the null model is correctly specified. Nevertheless, *l*_*approx*_ for marginal association tests was close to one in a real GWAS data application.

The proposed *l*_*approx*_ was derived under the assumption that each of genetic variants is an independently and identically distributed random variable, and also is independent of phenotype, environment, and covariates. Therefore, the scaled mean of test statistics computed from genotype data, simulated under the assumptions above, with other variables (phenotype, environment, and covariates) being fixed, must be close to *l*_*approx*_. The results with the artificial genotype data for the 684 samples are given in S3 Fig, and the plots were very similar to Fig 1, verifying our approximation.

**Fig 2:**
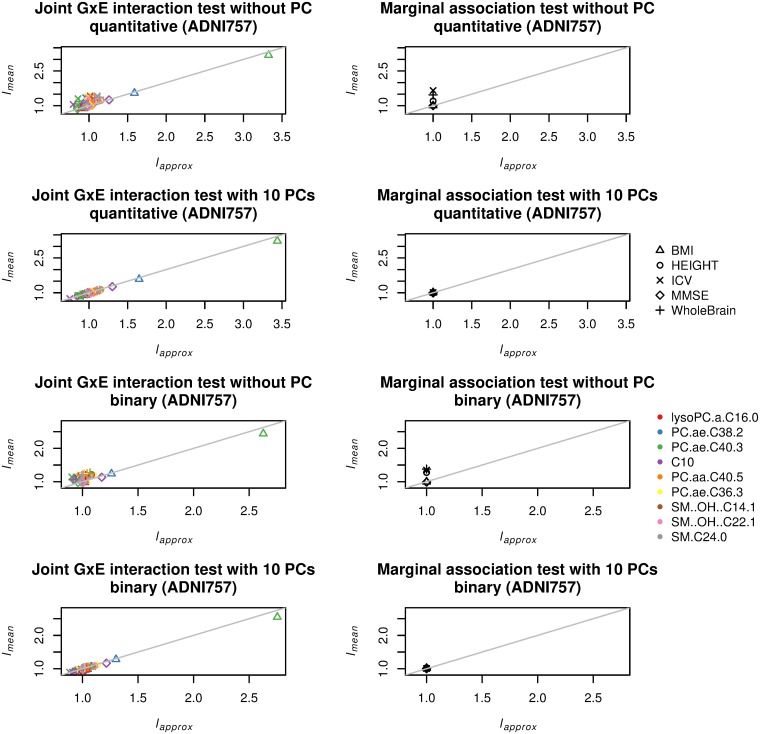
Comparison between *l*_*approx*_ and *l*_*mean*_ for joint GxE interaction and marginal association tests on ADNI-GWAS data for 757 samples which showed population stratification (ADNI757). Five phenotypes—height (HEIGHT), body mass index (BMI), whole brain (WholeBrain), intracranial volume (ICV), and mini mental state exam (MMSE)—and nine metabolite variables: lysoPC.a.C16.0, PC.ae.C38.2, PC.ae.C40.3, C10, PC.aa.C40.5, PC.ae.C36.3, SM‥OH‥C14.1, SM‥OH‥C22.1, and SM.C24.0. Results are shown without and with adjustment for top 10 principal components (PCs). Top left: joint GxE interaction test without PC adjustment (quantitative phenotype). Top right: marginal association test without PC adjustment (quantitative phenotype). Left in the second row: joint GxE interaction test with adjustment for top 10 PCs (quantitative phenotype). Right in the second row: marginal association test with adjustment for top 10 PCs (quantitative phenotype). Left in the third row: joint GxE interaction test without PC adjustment (binary phenotype created by dichotomizing quantitative phenotype). Right in the third row: marginal association test without PC adjustment (binary phenotype created by dichotomizing quantitative phenotype). Bottom left: joint GxE interaction test with adjustment for top 10 PCs (binary phenotype created by dichotomizing quantitative phenotype). Bottom right: marginal association test with adjustment for top 10 PCs (binary phenotype created by dichotomizing quantitative phenotype).

Fig 2 gives comparisons between *l*_*mean*_ and *l*_*approx*_ for joint GxE interaction and marginal association tests with the five phenotypes and nine metabolites as the environment variable on the ADNI dataset with 757 samples showing population stratification as seen in S1 Fig. The top left and right panels in Fig 2 are the results from joint GxE interaction and marginal association tests for quantitative phenotypes without PC adjustment, respectively. Unlike the case with 684 samples (the top left and right panels in Fig 1), there were points deviated from diagonal line in both panels (*l*_*approx*_ often underestimated *l*_*mean*_ for lower *l*_*mean*_). Results for binary phenotypes were in panels in the third row, and the similar deviations were observed. The left and right panels in the second row of Fig 2 are the results from joint GxE interaction and marginal association tests for quantitative phenotypes with adjustment for top 10 PCs, respectively. In this case, all points were distributed around the diagonal line, meaning that the adjustment by top 10 PCs could resolve the *l*_*approx*_’s underestimation of *l*_*mean*_. For binary phenotypes given in the bottom panels, *l*_*approx*_’s underestimation was resolved similarly. S4 Fig provides the results with adjustment for top 3 and top 5 PCs. No deviation was seen, implying that the PC adjustment was still successful.

The results with the artificial genotype data for the 757 samples are given in S5 Fig. Unlike Fig 2, there was no deviation between *l*_*mean*_ and *l*_*approx*_, which is the expected behavior since the simulated genotype data was generated under the assumption that *l*_*approx*_ is derived. It in turn implies that some of the assumptions for *l*_*approx*_ were violated in the cases of Fig 2 (the top panels and the panels in the third row) where deviation was observed, and population stratification could be one possible reason because PC adjustment resolved the deviation.

### Real data application: Detailed analysis of two datasets that showed large systematic inflation

**Fig 3:**
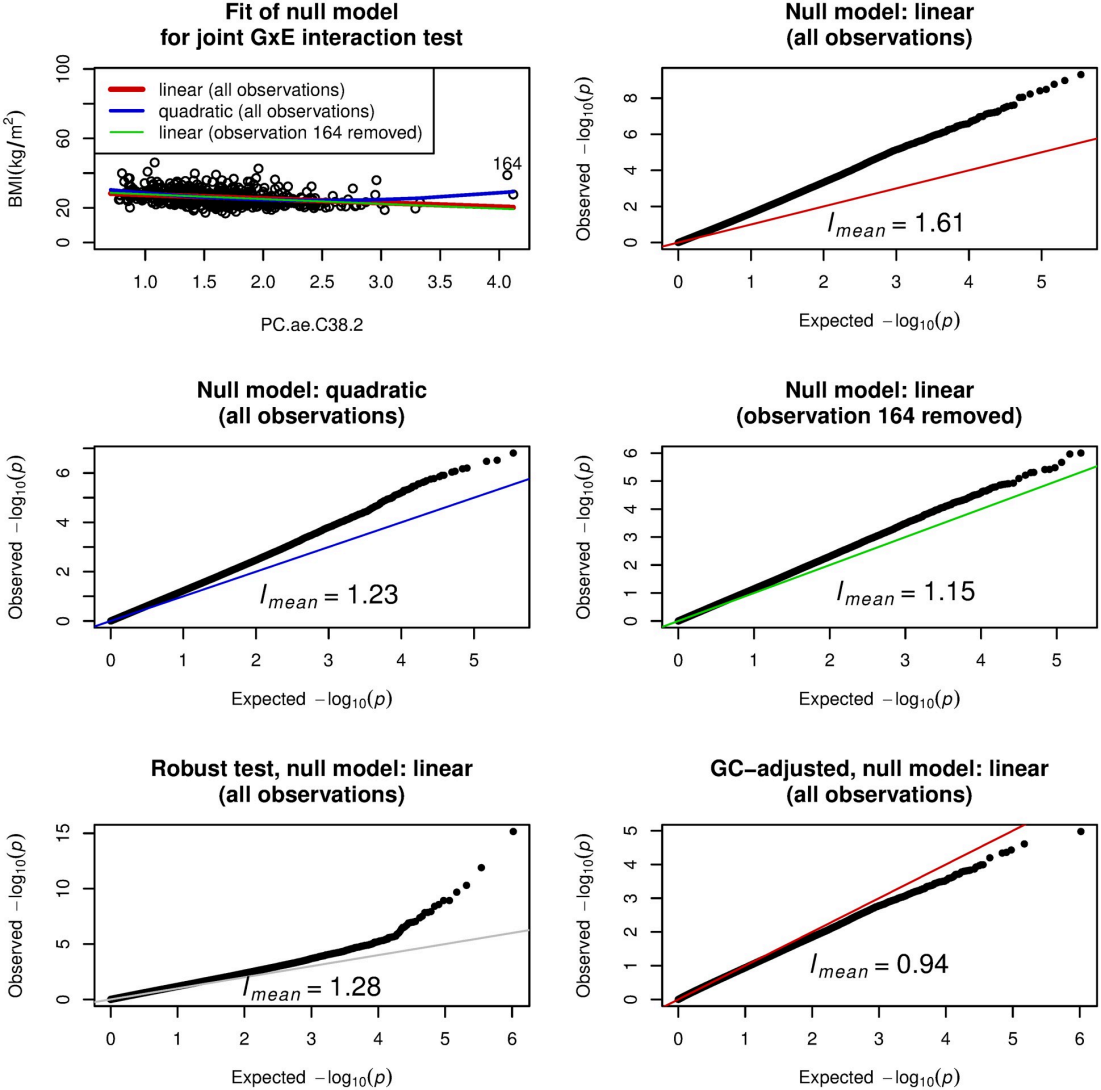
Systematic inflation in gene-PC.ae.C38.2 (a metabolite) interaction test for body mass index (BMI) on ADNI-GWAS data with 684 samples. Joint GxE interaction test applies to BMI as a quantitative phenotype and PC.ae.C38.2 as the environment variable by adjusting sex and age as covariates. Top left: scatter plot for BMI and PC.ae.C38.2 with emphasis on observation 164, which was the influential observation having Cook’s distance larger than the mean plus 4×sd (standard deviation). Three null models are given: linear (all observations), BMI = *β*_1_*G*_*l*_ + *β*_2_*G*_*l*_ × PC.ae.C38.2 + *γ*_1_ + *γ*_2_sex + *γ*_3_age + *γ*_4_PC.ae.C38.2 with *β*_1_ = *β*_2_ = 0 for *l*th genetic variant *G*_*l*_, quadratic (all observations), BMI = *β*_1_*G*_*l*_ + *β*_2_*G*_*l*_ × PC.ae.C38.2 + *γ*_1_ + *γ*_2_sex + *γ*_3_age + *γ*_4_PC.ae.C38.2 + *γ*_5_PC.ae.C38.2^2^ with *β*_1_ = *β*_2_ = 0 for *l*th genetic variant *G*_*l*_, linear (observation 164 removed), BMI = *β*_1_*G*_*l*_ + *β*_2_*G*_*l*_ × PC.ae.C38.2 + *γ*_1_ + *γ*_2_sex + *γ*_3_age + *γ*_4_PC.ae.C38.2 with *β*_1_ = *β*_2_ = 0 for *l*th genetic variant *G*_*l*_, where the influential observation (index 164) is removed. Top right: quantile-quantile (QQ) plot from joint GxE interaction test with the linear null model on all observations. Middle left: QQ plot from joint GxE interaction test with the quadratic null model for all observations. Middle right: QQ plot from joint GxE interaction test with the quadratic null model for which observation 164 is removed. Bottom left: QQ plot from the Almli’s robust joint GxE interaction test [16] with the linear null model for all observations. Bottom right: QQ plot from genomic control (GC) adjusted joint GxE interaction test with the linear null model for all observations. *l*_*mean*_, the scaled mean of genome-wide test statistics, is one if the null model is correctly specified.

Large discrepancy of *l*_*approx*_ from one suggests that the specified null model is problematic. Here, we investigated two analyses that showed large discrepancy of *l*_*approx*_ from one as described in the Materials and Methods section. Fig 3 gives the first example with PC.ae.C38.2 as the environment variable in the ADNI data with 684 samples. The value of *l*_*mean*_ was 1.61. A scatter plot of BMI versus the environment variable (Fig 3, top left) shows that a linear model for the environment variable is inadequate and suggests a quadratic model for better fit to the data. Given this consideration, we modified the null model by including a quadratic term, and indeed this mitigated the problematic behavior as shown in the middle left panel of Fig 3; *l*_*mean*_ was reduced to 1.23. Alternatively, the plot suggests that there may be outliers. We calculated the Cook’s distance [32] on linear regression model for BMI with explanatory variables, age, sex, and PC.ae.C38.2, and a single observation of index 164 had Cook’s distance larger than the mean plus 4×sd (standard deviation). See also S6 Fig for regression diagnostics plot. We emphasized the influential observation in the panel by showing the index 164. When this sample was removed, the null model with linear effect mitigated the systematic inflation behavior as shown in the middle right panel of Fig 3; *l*_*mean*_ was reduced to 1.15.

The result of the robust joint GxE interaction test using the Almli’s program is given in the bottom left panel of Fig 3. The systematic inflation still remained even when the robust test was applied. The result of the genomic control adjustment is given in the bottom right panel of Fig 3. The severe problematic behavior could not be resolved.

**Fig 4:**
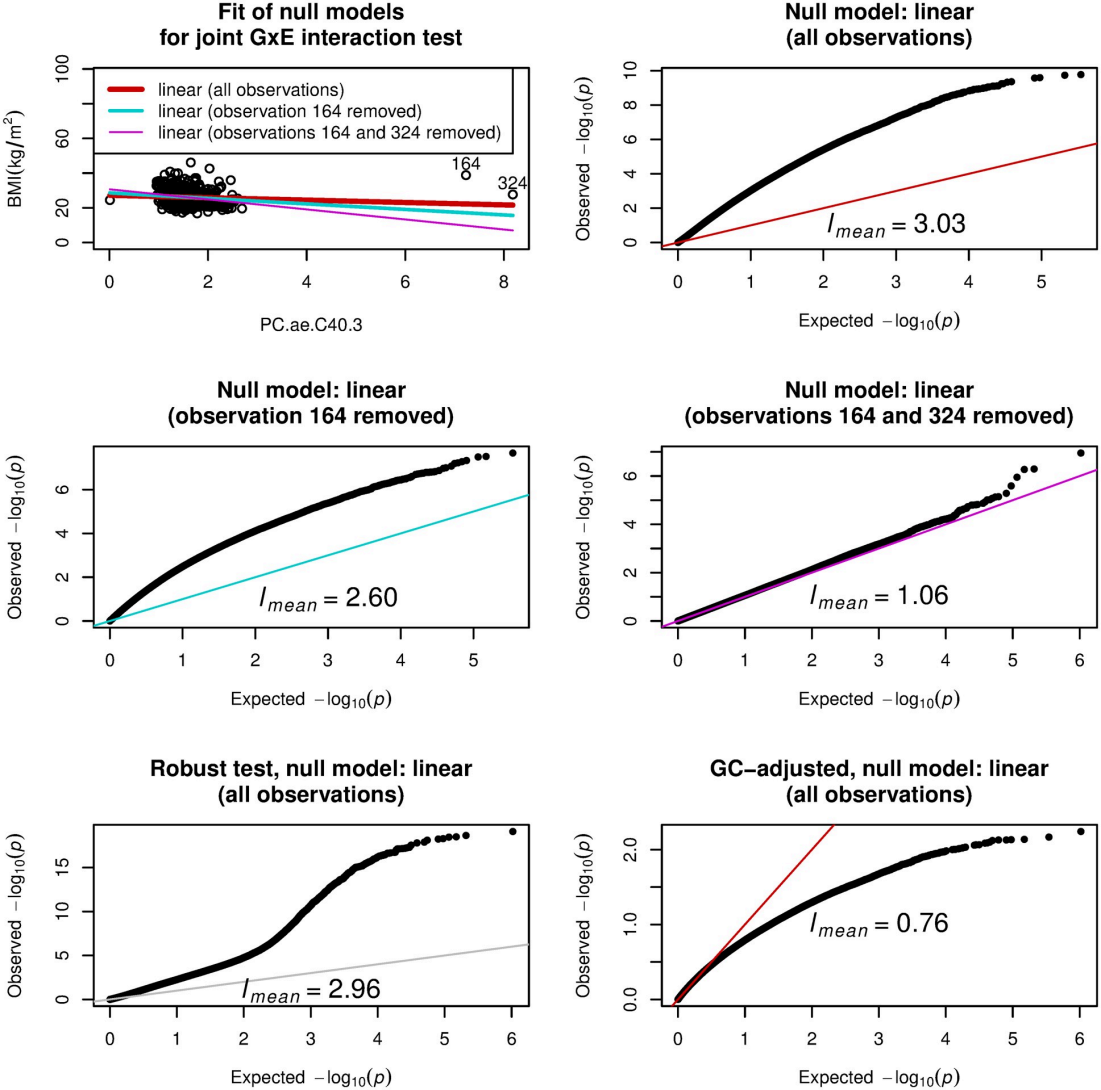
Systematic inflation in gene-PC.ae.C40.3 (a metabolite) interaction test for body mass index (BMI) on ADNI-GWAS data with 684 samples. Joint GxE interaction test is applied with BMI as a quantitative phenotype and PC.ae.C40.3 as the environment variable wit sex and age are adjusted. Top left: scatter plot for BMI and PC.ae.C40.3 with emphasis on observations 164 and 324, which were the influential observations having Cook’s distance larger than the mean plus 4×sd (standard deviation), where observation 164 has larger Cook’s distance than observation 324. Three null models are given: linear (all observations), BMI = *β*_1_*G*_*l*_ + *β*_2_*G*_*l*_ × PC.ae.C40.3 + *γ*_1_ + *γ*_2_sex + *γ*_3_age + *γ*_4_PC.ae.C40.3 with *β*_1_ = *β*_2_ = 0 for *l*th genetic variant *G*_*l*_, linear (observation 164 removed), BMI = *β*_1_*G*_*l*_ + *β*_2_*G*_*l*_ × PC.ae.C40.3 + *γ*_1_ + *γ*_2_sex + *γ*_3_age + *γ*_4_PC.ae.C40.3 with *β*_1_ = *β*_2_ = 0 for *l*th genetic variant *G*_*l*_, where the most influential observation (index 164) is removed, linear (observations 164 and 324 removed), BMI = *β*_1_*G*_*l*_ + *β*_2_*G*_*l*_ × PC.ae.C40.3 + *γ*_1_ + *γ*_2_sex + *γ*_3_age + *γ*_4_PC.ae.C40.3 with *β*_1_ = *β*_2_ = 0 for *l*th genetic variant *G*_*l*_, where two most influential observations (164 and 324) are removed. Top right: quantile-quantile (QQ) plot from joint GxE interaction test with the linear null model for all observations. Middle left: quantile-quantile (QQ) plot from joint GxE interaction test with the linear null model for which observation 164 is removed. Middle right: quantile-quantile (QQ) plot from joint GxE interaction test with the linear null model for which observations 164 and 324 are removed. Bottom left: QQ plot from the Almli’s robust joint GxE interaction test [16] with the linear null model for all observations. Bottom right: QQ plot from genomic control (GC) adjusted joint GxE interaction test with the linear null model for all observations. *l*_*mean*_, the scaled mean of genome-wide test statistics, is one if the null model is correctly specified.

Fig 4 gives the second example that uses PC.ae.C40.3 as the environment variable for BMI as phenotype in the ADNI data with 684 samples. The value of *l*_*mean*_ was 3.03. A scatter plot (Fig 4, top left) of BMI versus the environment variable suggests that two points labeled as 164 and 324 on the right side could be outliers. We calculated the Cook’s distance on linear regression model for BMI with explanatory variables, age, sex, and PC.ae.C40.3, and observations 164 and 324 had Cook’s distance larger than the mean plus 4×sd (standard deviation), where observation 164 had larger Cook’s distance than observation 324. See also S7 Fig for regression diagnostics plot. When observation 164 was removed, the null model with linear effect mitigated the systematic inflation behavior as shown in the middle left panel of Fig 3; *l*_*mean*_ was reduced to 2.6. When observations 164 and 324 were removed, the null model with linear effect further mitigated the systematic inflation behavior as shown in the middle right panel of Fig 3; *l*_*mean*_ was reduced to 1.06.

**Fig 5:**
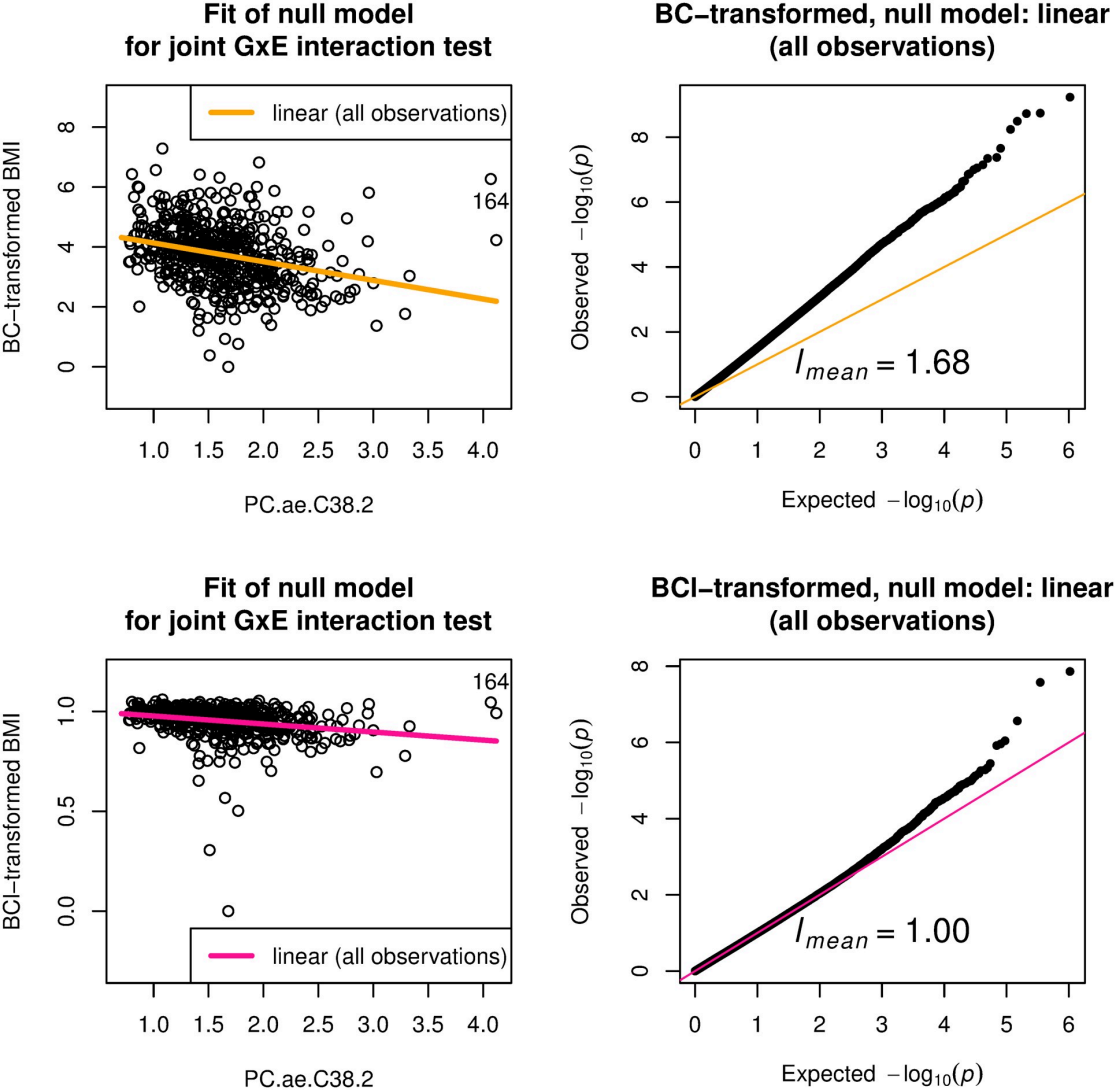
Gene-PC.ae.C38.2 (a metabolite) interaction test for Box–Cox transformed body mass index (BMI) on ADNI-GWAS data with 684 samples. Joint GxE interaction test applies to BMI as a quantitative phenotype and PC.ae.C38.2 as the environment variable with adjustment for sex and age. Top left: scatter plot for Box–Cox transformed BMI (BMI subtracted the minimum and then added 1) optimized in terms of normality (BC-transformed) and PC.ae.C38.2 with emphasis on observation 164, which was the influential observation having Cook’s distance (based on BMI without transformation) larger than the mean plus 4×sd (standard deviation). Linear null model is given, *BC*_normality_(BMI − min BMI + 1) = *γ*_1_ + *γ*_2_sex + *γ*_3_age + *γ*_4_PC.ae.C38.2, where *BC*_normality_ denotes the Box–Cox transformation with the optimal parameter determined by the closeness to normality of the transformed BMI. Top right: quantile-quantile (QQ) plot from joint GxE interaction test for BC-transformed BMI (BMI subtracted the minimum and then added 1) with the linear null model on all observations. Bottom left: scatter plot for Box–Cox transformed BMI (BMI subtracted the minimum and then added 1) optimized in terms of *l*_*approx*_ (BCl-transformed) and PC.ae.C38.2 with emphasis on observation 164, which was the influential observation having Cook’s distance (based on BMI without transformation) larger than the mean plus 4×sd (standard deviation). Linear null model is given, $BC_{l_{approx}}\left(\text{BMI}-\text{min}\;\text{BMI}+1\right)=\gamma_{1}+\gamma_{2}\text{sex}+\gamma_{3}\text{age}+\gamma_{4}\text{PC}.\text{ae}.C38.2$, where $BC_{l_{approx}}$ denotes the Box–Cox transformation with the optimal parameter determined by the closeness of *l*_*approx*_ to one. Bottom right: QQ plot from joint GxE interaction test for BCl-transformed BMI with the linear null model on all observations. *l*_*mean*_, the scaled mean of genome-wide test statistics, is one if the null model is correctly specified.

**Fig 6:**
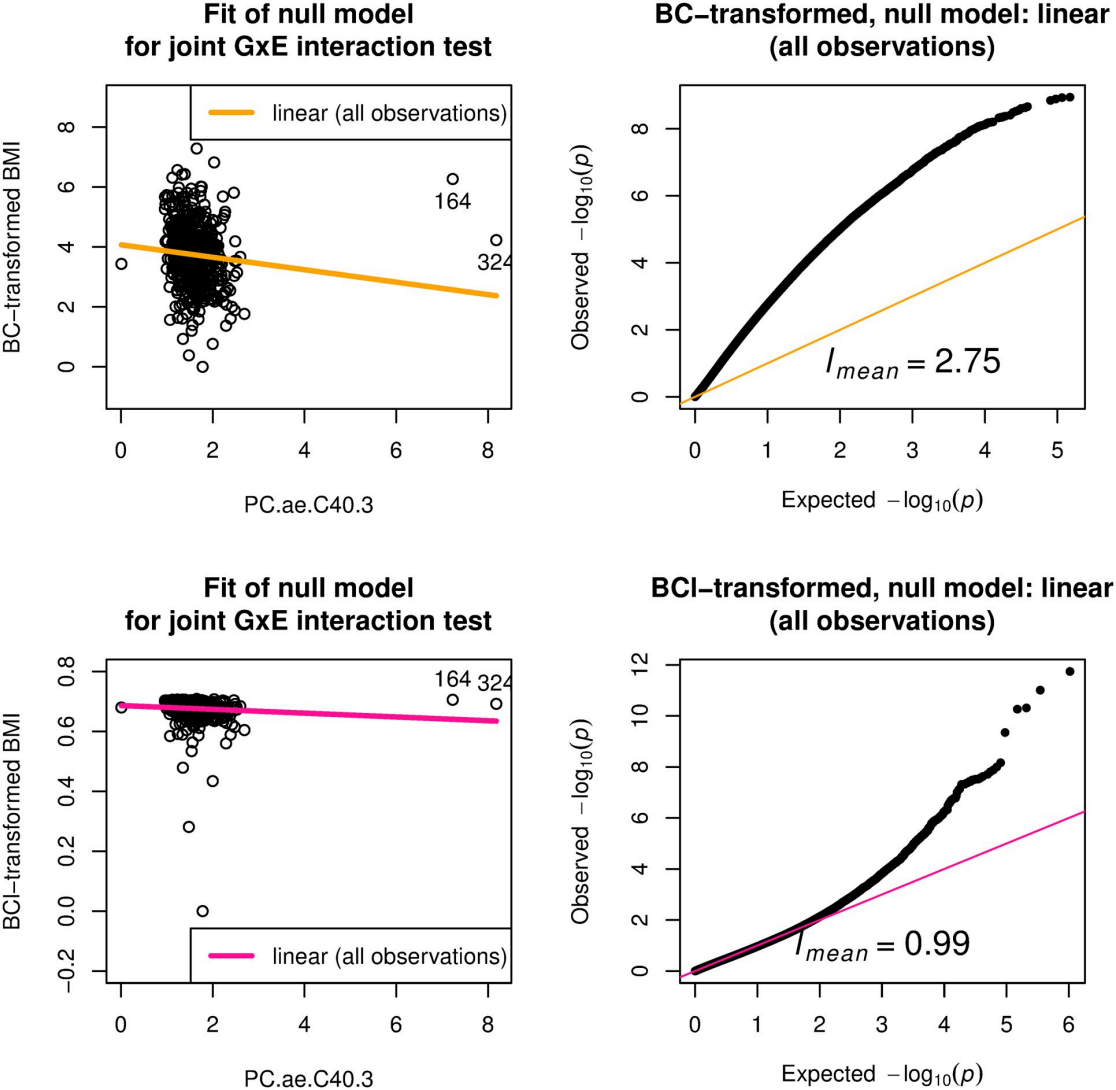
Gene-PC.ae.C40.3 (a metabolite) interaction test for Box–Cox transformed body mass index (BMI) on ADNI-GWAS data with 684 samples. Joint GxE interaction test applies to BMI as a quantitative phenotype and PC.ae.C40.3 as the environment variable with adjustment for sex and age. Top left: scatter plot for Box–Cox transformed BMI (BMI subtracted the minimum and then added 1) optimized in terms of normality (BC-transformed) and PC.ae.C40.3 with emphasis on observations 164 and 324, which were the influential observations having Cook’s distance (based on BMI without transformation) larger than the mean plus 4×sd (standard deviation). Linear null model is given, *BC*_normality_(BMI − min BMI + 1) = *γ*_1_ + *γ*_2_sex + *γ*_3_age + *γ*_4_PC.ae.C40.3, where *BC*_normality_ denotes the Box–Cox transformation with the optimal parameter determined by the closeness to normality of the transformed BMI. Top right: quantile-quantile (QQ) plot from joint GxE interaction test for BC-transformed BMI with the linear null model on all observations. Bottom left: scatter plot for Box–Cox transformed BMI (BMI subtracted the minimum and then added 1) optimized in terms of *l*_*approx*_ (BCl-transformed) and PC.ae.C40.3 with emphasis on observations 164 and 324, which were the influential observations having Cook’s distance (based on BMI without transformation) larger than the mean plus 4×sd (standard deviation). Linear null model is given, $BC_{l_{approx}}\left(\text{BMI}-\text{min}\;\text{BMI}+1\right)=\gamma_{1}+\gamma_{2}\text{sex}+\gamma_{3}\text{age}+\gamma_{4}\text{PC}.\text{ae}.C40.3$, where $BC_{l_{approx}}$ denotes the Box–Cox transformation with the optimal parameter determined by the closeness of *l*_*approx*_ to one. Bottom right: QQ plot from joint GxE interaction test for BCl-transformed BMI with the linear null model on all observations. *l*_*mean*_, the scaled mean of genome-wide test statistics, is one if the null model is correctly specified.

Figs 5 and 6 provide results of the BMI after Box–Cox transformation for the data with the 684 and 757 samples, respectively. The Box–Cox transformation of BMI based on normality was insufficient to reduce the systematic inflation, as seen in the top right panels of Figs 5 and 6. On the other hand, the Box–Cox transformation of BMI based on *l*_*mean*_ reduced the systematic inflation, as seen in the bottom right panels of Figs 5 and 6.

### Simulation studies

Here, we provide the results of joint GxE interaction and marginal association tests from various simulation studies. Tables 2 and 3 include particularly noteworthy results while S2 and S3 Tables include the remainings. In derivation of *l*_*approx*_, we have assumed that genotypes are not associated with phenotypes, environment and covariate variables. Simulation scenarios (*b*_*G*_, *b*_*Z*_, *b*_*GE*_) = (0, 0, 0) and (0, 1, 0) satisfy the above assumptions except in scenarios 1a, 1b, 1c, 2a, and 2b. In such cases, the approximation of *l*_*approx*_ to *l*_*mean*_ was well as the ratio *l*_*approx*_/*l*_*mean*_ was close to one. On the other hand, in scenarios (*b*_*G*_, *b*_*Z*_, *b*_*GE*_) = (1, 0, 0) and (0, 0, 1), phenotypes are associated with genotypes, and hence the assumptions are not satisfied. In such cases, there is no guarantee that *l*_*approx*_ approaches to *l*_*mean*_ even if *n* is increased. Actually, there were several scenarios showing the ratio *l*_*approx*_/*l*_*mean*_ for *n* = 10000 that was more different from one than that for *n* = 1000. We provide brief summaries not mentioned above.

- Association among environment, covariate variables and/or genotypes (Scenarios 1a, 1b, 1c and 1d). In scenarios (*b*_*G*_, *b*_*Z*_, *b*_*GE*_) = (0, 0, 0) and (0, 1, 0) under the presence of the association between covariate and environment variables, the assumption for the derivation of *l*_*approx*_ holds, that is, genotypes are not associated with phenotype, covariate/environment variables. Indeed, *l*_*approx*_ approximated *l*_*mean*_ well. On the other hand, in scenario (*b*_*G*_, *b*_*Z*_, *b*_*GE*_) = (0, 1, 0) under the presence of the association between genotype and covariate/environment variables, the assumption for the derivation of *l*_*approx*_ does not hold. Nevertheless, *l*_*approx*_ well approximated *l*_*mean*_. Scenarios (*b*_*G*_, *b*_*Z*_, *b*_*GE*_) = (1, 0, 0) and (0, 0, 1) correspond to the presence of the genetic effect, meaning that the approximation is not guaranteed. Actually, *l*_*approx*_ was deviated from *l*_*mean*_ in many cases. The magnitude of deviation differed depending on the setup of environment/covariate variables. The results of scenario 1d in S2 and S3 Tables showed that the presence of the association between the covariate and environment variables did not make much difference from the baseline scenario.
- Misspecified null model (Scenarios 2a, 2b, 2c, 2d, and 2e). In some scenarios, *l*_*mean*_ took an extremely large value, and then, *l*_*approx*_ had a large value correspondingly. In scenarios 2a and 2b (i.e. covariate associated with genotypes was missed in the null model), no deviation between *l*_*approx*_ and *l*_*mean*_ was observed when no genotypic effect exist (i.e. (*b*_*G*_, *b*_*Z*_, *b*_*GE*_) = (0, 0, 0) and (0, 1, 0)), but *l*_*approx*_ was close to one while *l*_*mean*_ deviated from one in other cases (i.e. (*b*_*G*_, *b*_*Z*_, *b*_*GE*_) = (1, 0, 0) and (0, 0, 1)). The misspecified functional form of the null model (i.e. scenario 2c) gave larger *l*_*mean*_, and *l*_*approx*_ could approximate the inflated *l*_*mean*_. The existence of outlier(s) tended to give large *l*_*mean*_ just as in Figs 3 and 4. *l*_*mean*_ in scenario 2d (i.e. one outlier) was larger than that in scenario 2e (i.e. ten outliers).
- Environment/covariate variable distribution (Scenarios 3a, 3b, 3c, and 3d). Under the absence of the genetic effect (i.e. (*b*_*G*_, *b*_*Z*_, *b*_*GE*_) = (0, 0, 0) and (0, 1, 0)), the approximation of *l*_*approx*_ to *l*_*mean*_ looked well. Under the presence of the genetic effect (i.e. (*b*_*G*_, *b*_*Z*_, *b*_*GE*_) = (1, 0, 0) and (0, 0, 1)), where the approximation is not guaranteed, *l*_*approx*_ was deviated from *l*_*mean*_ as expected. The magnitude of deviation differed depending on the setup of environment/covariate variables.
- Genotype distribution (Scenarios 4a, 4b, and 4c). The difference in MAF distribution gave no much difference in the approximation as our derivation does not require specific MAF distribution. Correlation between genotypes did not alter the approximation in terms of mean values of *l*_*approx*_ and *l*_*mean*_, but the result under the presence of correlation was more variable than the result under the absence of correlation. This is perhaps due to that the correlation between genetic variants reduced the effective number of independent loci.

**Table 2:** Comparison between *l*_*approx*_ and *l*_*mean*_ in quantitative phenotype simulations.

Scenario		GxE (*n* = 1000)			Marginal (*n* = 1000)			GxE (*n* = 10000)			Marginal (*n* = 10000)		
(*b*_*G*_, *b*_*Z*_, *b*_*GE*_)		*l*_*approx*_	*l*_*mean*_	$\frac{l_{approx}}{l_{mean}}$	*l*_*approx*_	*l*_*mean*_	$\frac{l_{approx}}{l_{mean}}$	*l*_*approx*_	*l*_*mean*_	$\frac{l_{approx}}{l_{mean}}$	*l*_*approx*_	*l*_*mean*_	$\frac{l_{approx}}{l_{mean}}$
Base	(0,0,0)	1.00	1.01	1.00	1.00	1.01	1.00	1.00	1.00	1.00	1.00	1.00	1.00
		(0.03)	(0.04)	(0.02)	(0.00)	(0.03)	(0.03)	(0.01)	(0.02)	(0.02)	(0.00)	(0.03)	(0.03)
Base	(1,0,0)	1.01	1.12	0.90	1.00	1.23	0.82	1.00	2.02	0.49	1.00	3.04	0.33
		(0.03)	(0.04)	(0.02)	(0.00)	(0.04)	(0.03)	(0.01)	(0.04)	(0.01)	(0.00)	(0.08)	(0.01)
Base	(0,1,0)	1.01	1.01	1.00	1.00	1.01	1.00	1.00	1.00	1.00	1.00	1.00	1.00
		(0.04)	(0.04)	(0.02)	(0.00)	(0.03)	(0.03)	(0.01)	(0.02)	(0.02)	(0.00)	(0.03)	(0.03)
Base	(0,0,1)	1.05	1.06	0.99	1.00	1.01	0.99	1.31	2.41	0.54	1.00	1.00	1.00
		(0.04)	(0.05)	(0.02)	(0.00)	(0.03)	(0.03)	(0.02)	(0.07)	(0.01)	(0.00)	(0.03)	(0.03)
1a	(0,0,0)	1.01	1.01	1.00	1.00	1.01	1.00	1.00	1.00	1.00	1.00	1.00	1.00
		(0.03)	(0.04)	(0.02)	(0.00)	(0.03)	(0.03)	(0.01)	(0.02)	(0.02)	(0.00)	(0.03)	(0.03)
1a	(1,0,0)	1.01	1.07	0.94	1.00	1.13	0.89	1.00	1.92	0.52	1.00	2.84	0.35
		(0.03)	(0.04)	(0.02)	(0.00)	(0.04)	(0.03)	(0.01)	(0.04)	(0.01)	(0.00)	(0.07)	0.01)
1a	(0,1,0)	1.00	1.01	1.00	1.00	1.01	1.00	1.00	1.00	1.00	1.00	1.00	1.00
		(0.03)	(0.04)	(0.02)	(0.00)	(0.03)	(0.03)	(0.01)	(0.02)	(0.02)	(0.00)	(0.03)	(0.03)
1a	(0,0,1)	1.29	1.39	0.93	1.00	1.01	0.99	1.32	2.47	0.53	1.00	1.00	1.00
		(0.08)	(0.09)	(0.02)	(0.00)	(0.03)	(0.03)	(0.02)	(0.07)	(0.01)	(0.00)	(0.03)	(0.03)
2a	(0,0,0)	1.01	1.01	1.00	1.00	1.00	1.00	1.00	1.00	1.00	1.00	1.00	1.00
		(0.03)	(0.04)	(0.02)	(0.00)	(0.03)	(0.03)	(0.01)	(0.02)	(0.02)	(0.00)	(0.03)	(0.03)
2a	(1,0,0)	1.00	1.13	0.89	1.00	1.25	0.80	1.00	2.26	0.44	1.00	3.51	0.29
		(0.03)	(0.04)	(0.02)	(0.00)	(0.05)	(0.03)	(0.01)	(0.05)	(0.01)	(0.00)	(0.09)	(0.01)
2a	(0,1,0)	1.00	1.00	1.00	1.00	1.00	1.00	1.00	1.00	1.00	1.00	1.00	1.00
		(0.03)	(0.04)	(0.02)	(0.00)	(0.03)	(0.03)	(0.01)	(0.02)	(0.02)	(0.00)	(0.03)	(0.03)
2a	(0,0,1)	1.34	1.46	0.92	1.00	1.00	1.00	1.23	1.99	0.62	1.00	1.00	1.00
		(0.07)	(0.08)	(0.02)	(0.00)	(0.03)	(0.03)	(0.02)	(0.06)	(0.01)	(0.00)	(0.03)	(0.03)
2c	(0,0,0)	2.25	2.24	1.01	1.00	1.00	1.00	2.34	2.34	1.00	1.00	1.00	1.00
		(0.24)	(0.24)	(0.03)	(0.00)	(0.03)	(0.03)	(0.08)	(0.11)	(0.03)	(0.00)	(0.03)	(0.03)
2c	(1,0,0)	2.16	2.20	0.98	1.00	1.09	0.93	2.27	2.39	0.95	1.00	1.23	0.81
		(0.25)	(0.26)	(0.03)	(0.00)	(0.04)	(0.03)	(0.08)	(0.11)	(0.02)	(0.00)	(0.04)	(0.02)
2c	(0,1,0)	2.30	2.29	1.00	1.00	1.00	1.00	2.33	2.34	1.00	1.00	1.00	1.00
		(0.23)	(0.23)	(0.03)	(0.00)	(0.03)	(0.03)	(0.09)	(0.11)	(0.03)	(0.00)	(0.03)	(0.03)
2c	(0,0,1)	2.30	2.33	0.99	1.00	1.00	1.00	2.31	2.63	0.88	1.00	1.00	1.00
		(0.23)	(0.23)	(0.03)	(0.00)	(0.03)	(0.03)	(0.10)	(0.13)	(0.02)	(0.00)	(0.03)	(0.03)
2d	(0,0,0)	1.00	1.00	0.99	1.00	1.00	1.00	0.99	1.00	0.99	1.00	1.00	1.00
		(0.30)	(0.22)	(0.07)	(0.00)	(0.03)	(0.03)	(0.23)	(0.17)	(0.05)	(0.00)	(0.03)	(0.03)
2d	(1,0,0)	0.99	1.11	0.87	1.00	1.24	0.81	0.98	1.29	0.76	1.00	1.60	0.63
		(0.36)	(0.26)	(0.08)	(0.00)	(0.04)	(0.03)	(0.20)	(0.16)	(0.06)	(0.00)	(0.05)	(0.02)
2d	(0,1,0)	49.69	35.91	1.38	1.00	1.00	1.00	190.26	139.98	1.36	1.00	1.00	1.00
		(6.77)	(4.93)	(0.02)	(0.00)	(0.03)	(0.03)	(10.68)	(8.48)	(0.03)	(0.00)	(0.03)	(0.03)
2d	(0,0,1)	13.70	10.09	1.36	1.00	1.01	1.00	24.05	18.15	1.32	1.00	1.00	1.00
		(3.75)	(2.68)	(0.03)	(0.00)	(0.03)	(0.03)	(4.29)	(3.10)	(0.03)	(0.00)	(0.03)	(0.03)

Simulation results from 200 replicates under scenarios Base, 1a, 2a, 2c, and 2d with four effect size scenarios given as triplets (*b*_*G*_, *b*_*Z*_, *b*_*GE*_):(0, 0, 0), (1, 0, 0), (0, 1, 0), and (0, 0, 1) with additive genotype coding. The values are the means and standard errors (in parentheses) of the proposed approximation (*l*_*approx*_), the scaled mean test statistics (*l*_*mean*_) and the ratio (*l*_*approx*_/*l*_*mean*_) for joint GxE interaction and marginal association tests. *b*_*G*_, *b*_*Z*_, and *b*_*GE*_ are parameters of genotype, covariate and GxE interaction effects, respectively (0 corresponds to no effect); *n* denotes the sample size.

**Table 3:** Comparison between *l*_*approx*_ and *l*_*mean*_ in binary phenotype simulations.

Scenario		GxE (*n* = 1000)			Marginal (*n* = 1000)			GxE (*n* = 10000)			Marginal (*n* = 10000)		
(*b*_*G*_, *b*_*Z*_, *b*_*GE*_)		*l*_*approx*_	*l*_*mean*_	$\frac{l_{approx}}{l_{mean}}$	*l*_*approx*_	*l*_*mean*_	$\frac{l_{approx}}{l_{mean}}$	*l*_*approx*_	*l*_*mean*_	$\frac{l_{approx}}{l_{mean}}$	*l*_*approx*_	*l*_*mean*_	$\frac{l_{approx}}{l_{mean}}$
Base	(0,0,0)	1.00	1.00	1.00	1.00	1.00	1.00	1.00	1.00	1.00	1.00	1.00	1.00
		(0.00)	(0.02)	(0.02)	(0.00)	(0.03)	(0.03)	(0.00)	(0.02)	(0.02)	(0.00)	(0.03)	(0.03)
Base	(1,0,0)	1.00	1.02	0.99	1.00	1.03	0.98	1.00	1.00	1.00	1.00	1.00	1.00
		(0.01)	(0.02)	(0.02)	(0.00)	(0.03)	(0.03)	(0.00)	(0.02)	(0.02)	(0.00)	(0.03)	(0.03)
Base	(0,1,0)	1.00	1.00	1.00	1.00	1.00	1.00	1.00	1.00	1.00	1.00	1.00	1.00
		(0.02)	(0.03)	(0.02)	(0.00)	(0.03)	(0.03)	(0.01)	(0.02)	(0.02)	(0.00)	(0.03)	(0.03)
Base	(0,0,1)	1.03	1.05	0.98	1.00	1.00	1.00	1.01	1.01	1.00	1.00	1.00	1.00
		(0.02)	(0.03)	(0.02)	(0.00)	(0.03)	(0.03)	(0.00)	(0.02)	(0.02)	(0.00)	(0.03)	(0.03)
1a	(0,0,0)	1.00	1.00	1.00	1.00	1.00	1.00	1.00	1.00	1.00	1.00	1.00	1.00
		(0.00)	(0.02)	(0.02)	(0.00)	(0.03)	(0.03)	(0.00)	(0.02)	(0.02)	(0.00)	(0.03)	(0.03)
1a	(1,0,0)	1.00	1.01	0.99	1.00	1.02	0.99	1.00	1.00	1.00	1.00	1.00	1.00
		(0.00)	(0.02)	(0.02)	(0.00)	(0.03)	(0.03)	(0.00)	(0.02)	(0.02)	(0.00)	(0.03)	(0.03)
1a	(0,1,0)	1.00	1.01	1.00	1.00	1.00	1.00	1.00	1.00	1.00	1.00	1.00	1.00
		(0.02)	(0.03)	(0.02)	(0.00)	(0.04)	(0.04)	(0.01)	(0.02)	(0.02)	(0.00)	(0.03)	(0.03)
1a	(0,0,1)	1.03	1.05	0.98	1.00	1.00	1.00	1.01	1.01	1.00	1.00	1.00	1.00
		(0.02)	(0.03)	(0.02)	(0.00)	(0.03)	(0.03)	(0.00)	(0.02)	(0.02)	(0.00)	(0.03)	(0.03)
2a	(0,0,0)	1.00	1.00	1.00	1.00	1.00	1.00	1.00	1.00	1.00	1.00	1.00	1.00
		(0.00)	(0.02)	(0.02)	(0.00)	(0.03)	(0.03)	(0.00)	(0.02)	(0.02)	(0.00)	(0.03)	(0.03)
2a	(1,0,0)	1.00	1.02	0.98	1.00	1.04	0.97	1.00	1.00	1.00	1.00	1.00	1.00
		(0.01)	(0.03)	(0.02)	(0.00)	(0.04)	(0.04)	(0.00)	(0.02)	(0.02)	(0.00)	(0.03)	(0.03)
2a	(0,1,0)	1.00	1.00	1.00	1.00	1.00	1.00	1.00	1.00	1.00	1.00	1.00	1.00
		(0.02)	(0.03)	(0.02)	(0.00)	(0.03)	(0.03)	(0.00)	(0.02)	(0.02)	(0.00)	(0.03)	(0.03)
2a	(0,0,1)	1.03	1.05	0.98	1.00	1.00	1.00	1.01	1.01	1.00	1.00	1.00	1.00
		(0.03)	(0.04)	(0.02)	(0.00)	(0.03)	(0.03)	(0.00)	(0.02)	(0.02)	(0.00)	(0.03)	(0.04)
2c	(0,0,0)	0.86	0.86	1.00	1.00	1.00	1.00	0.85	0.85	1.00	1.00	1.00	1.00
		(0.01)	(0.02)	(0.02)	(0.00)	(0.03)	(0.03)	(0.00)	(0.02)	(0.02)	(0.00)	(0.03)	(0.03)
2c	(1,0,0)	0.92	0.94	0.98	1.00	1.03	0.97	0.88	0.89	1.00	1.00	1.00	1.00
		(0.02)	(0.02)	(0.02)	(0.00)	(0.03)	(0.03)	(0.00)	(0.02)	(0.02)	(0.00)	(0.03)	(0.03)
2c	(0,1,0)	0.89	0.90	1.00	1.01	1.00	1.00	0.89	0.89	1.00	1.00	1.00	1.00
		(0.02)	(0.03)	(0.02)	(0.00)	(0.03)	(0.03)	(0.01)	(0.02)	(0.02)	(0.00)	(0.03)	(0.03)
2c	(0,0,1)	0.86	0.86	1.00	1.00	1.01	1.00	0.95	0.95	1.00	1.01	1.03	0.98
		(0.01)	(0.03)	(0.02)	(0.00)	(0.03)	(0.03)	(0.01)	(0.02)	(0.02)	(0.00)	(0.03)	(0.03)
2d	(0,0,0)	0.99	1.00	0.99	1.00	1.01	1.00	1.02	1.01	1.00	1.00	1.00	1.01
		(0.19)	(0.14)	(0.05)	(0.00)	(0.03)	(0.03)	(0.16)	(0.12)	(0.04)	(0.00)	(0.03)	(0.03)
2d	(1,0,0)	1.03	1.03	0.99	1.00	1.03	0.97	1.03	1.02	1.01	1.00	1.00	1.00
		(0.26)	(0.18)	(0.06)	(0.00)	(0.03)	(0.03)	(0.17)	(0.13)	(0.04)	(0.00)	(0.03)	(0.03)
2d	(0,1,0)	9.15	5.86	1.30	1.00	1.00	1.00	2.15	2.13	1.01	1.00	1.00	1.00
		(8.73)	(4.97)	(0.34)	(0.01)	(0.03)	(0.03)	(1.17)	(1.15)	(0.03)	(0.00)	(0.03)	(0.03)
2d	(0,0,1)	11.34	6.65	1.41	0.99	0.99	1.00	2.11	2.08	1.01	0.99	0.99	1.00
		(9.50)	(5.09)	(0.41)	(0.01)	(0.03)	(0.03)	(1.18)	(1.15)	(0.03)	(0.01)	(0.03)	(0.03)

Simulation results from 200 replicates under scenarios Base, 1a, 2a, 2c, and 2d with four effect size scenarios given as triplets (*b*_*G*_, *b*_*Z*_, *b*_*GE*_):(0, 0, 0), (1, 0, 0), (0, 1, 0), and (0, 0, 1) with additive genotype coding. The values are the means and standard errors (in parentheses) of the proposed approximation (*l*_*approx*_), the scaled mean test statistics (*l*_*mean*_) and the ratio (*l*_*approx*_/*l*_*mean*_) for joint GxE interaction and marginal association tests. *b*_*G*_, *b*_*Z*_, and *b*_*GE*_ are parameters of genotype, covariate and GxE interaction effects, respectively (0 corresponds to no effect); *n* denotes the sample size.

For binary phenotype simulations (i.e. Table 3 and S3 Table), *l*_*mean*_s were less deviated from one compared with the results on quantitative phenotypes (i.e. Table 2 and S2 Table), probably due to the fact that binary phenotype has lower variation than quantitative phenotype, but the overall tendency was similar to the quantitative simulations. Regarding genotype coding, the additive coding gave larger discrepancy of *l*_*approx*_ from *l*_*mean*_ than recessive and dominant codings, and the recessive coding resulted in smallest discrepancy, which is due to the assignment of effect size 1 regardless of genotype coding.

## Discussion

In this paper, we presented a novel closed-form approximation to the mean of the chi-squared statistics for genome-wide joint GxE interaction tests by assuming that the null model is well specified. Interestingly, characteristics of genetic loci no longer appear in the approximation formula. It allows quick assessment of systematic inflation/deflation due to null model misspecification without requiring a genome-wide scan. To the best of our knowledge, there are no approach comparable to our proposed method. The approximation formula is particularly useful when many null models for GxE interaction analyses must be handled, e.g. with hundreds of environment variables such as the metabolites considered in this paper. For example, our examples in Figs 3 and 4 showing systematic inflation were identified by applying the proposed statistic; it would have been laborious work if all null models with 117 metabolites for large number of phenotypes were exhaustively investigated by genome-wide scan as well as by detailed examination of null models, because the procedure involves various visual inspections.

Once problematic null models have been detected, detailed investigation of adequacy of the null model must be made, for example by standard regression diagnostics, e.g. modeling of covariate effects, presence of outliers and influential samples, or heteroscedasticity. We also showed that existing methods—the robust test and genomic control-adjustment—may not always work. Note that the robust test differs from regression methods in robust statistics [33, 34] in which the focus is on issues owing to outliers. The null model misspecification may arise not only by outliers but also by misspecification of functional form or statistical model. The robust test only accounts for the impact of misspecification on the variance, whereas no correction is made for bias, which in general depends on the true model and thus is difficult to correct without additional modeling assumption. If bias is not negligible, the robust test may fail to resolve the problematic behavior. Rendering the null models more sophisticated, such as by better specifying the environment variable effect or by removing outliers, could reduce systematic inflation or deflation. Manual removal of outliers is not straightforward, in particular, when a large number of covariates are used. In this case, robust linear [33, 34] or logistic regression [35] are attractive approaches. If the misspecification of the functional form of the environment variable is the main concern, it is worth to apply the method recently developed by [36].

Theoretically, the test statistic for the marginal association test is inflated from $\chi_{1}^{2}$ to $\lambda \chi_{1}^{2}$ under the presence of population stratification [17, 24, 37, 38]. In this situation, the test statistics divided by the inflation factor λ follow $\chi_{1}^{2}$ distribution, which is the key idea of the genomic control method [17]. In contrast, in the case of problematic behavior for the joint GxE interaction tests due to null model misspecification, it is unclear what the distribution of test statistics is. The examples above demonstrate that genomic control-adjusted p-values do not always work. The above additional analyses also suggest that a more sophisticated null model or suitable statistical method could resolve the problematic behavior.

We also showed that the standard Box–Cox transformation based on normality may not always resolve the problematic behavior, whereas the Box–Cox transformation based on *l*_*approx*_ can directly resolve systematic inflation. Although we recommend detailed investigation of problematic null models, the Box–Cox transformation based on *l*_*approx*_ can provide a quick solution.

The proposed approximation is derived by assuming that the genotype data at each locus are independently and identically distributed across subjects (but the distribution across loci can differ and be correlated due to linkage disequilibrium). This simplification enabled us to derive a closed-form formula. On the one hand, our theory does not cover the presence of population stratification, which is the limitation of our approach. Indeed, we observed situations where the approximation failed in the presence of population stratification (Fig 2). Interestingly, the failure disappeared by adjusting for PCs estimated from genome-wide SNP data. The extensibility of the phenomenon to other datasets is an interesting future topic.

The statistic *l*_*approx*_ quantifies problematic behavior based on the extent of the discrepancy from one, as with the genomic inflation factor. For example, we can roughly judge that null models having *l*_*approx*_ within the range [0.9, 1.1] or [0.95, 1.05] are not a problem, so that a genome-wide scan can be safely conducted.

In derivation of the approximation formula, we imposed the technical condition of no genetic effect on phenotype, covariates, and environment variables. This assumption may not necessarily hold in real GWAS data. However, as seen in the real ADNI data application, the approximation worked well in most of the cases. The simulation studies also showed that the approximation worked under the scenarios with no genetic effect on phenotype (i.e. (*b*_*G*_, *b*_*Z*_, *b*_*GE*_) = (0, 0, 0) and (0, 1, 0)). In real GWAS, we sometimes encounter the situation where the marginal association test gives a very limited number of loci being genome-wide significant while other loci follow the expected null distribution (as checked by the quantile–quantile plot of genome-wide p-values), implying that the majority of loci have approximately no genetic effect on phenotype. In such cases, we expect that the approximation works well.

In contrast, if many loci have effect on phenotype, covariates, and environment variables, the above assumption does not hold, e.g. under the polygenic architecture [39] or in the candidate gene studies. As seen in the simulation studies as well as in the ADNI data application with 757 samples (c.f. Fig 2), the presence of genetic effect on phenotype yielded discrepancy between *l*_*approx*_ and *l*_*mean*_. In the cases with *l*_*approx*_ far from one, *l*_*mean*_ was also far from one, meaning that *l*_*approx*_ far from one indicates systematic inflation or deflation. In contrast, *l*_*approx*_ close to one does not guarantee the absence of systematic inflation or deflation. Simulation scenarios 2a and 2b correspond to the above phenomenon. Specifically, ignorance of covariates associated with genotypes and phenotype made a deviation of *l*_*mean*_ from one but *l*_*approx*_ was still close to one. In this case, *l*_*approx*_ was unable to detect the systematic inflation, which means that the proposed approximation fails if confounding factors (or the surrogate variables) are unavailable and the genetic variants caused the problem, e.g. due to population stratification as in the ADNI data with 757 samples (c.f. Fig 2).

One might consider that traditional goodness-of-fit tests may be used just like *l*_*approx*_. However, this would reject most of the null models when the sample size is very large because the test requires correct specification of the true model, which is rarely achieved with real data [40]. On the other hand, the criterion based on *l*_*approx*_ allows one to explore null models that give acceptably less systematic departure of the test statistics distribution. The requirement is less strict than the correct model specification needed for goodness-of-fit tests. Our approximation is for the score statistics. For the Wald and likelihood ratio tests, *l*_*approx*_ is still useful, at least for the purpose of identifying null model misspecification, because the Wald and likelihood ratio tests are asymptotically the same as the score test.

We conclude that our proposed approximation is useful to quickly assess systematic inflation/deflation due to null model misspecification without requiring a genome-wide scan. It helps researchers to reconsider and improve null model specification. The benefit should be great when many covariates and environment variables are considered.

## Supporting information

S1 Fig 1st and 2nd PCs (principal components) plot for 757 individuals in ADNI data. PCs were computed by the EIGENSOFT package using GWAS data with 757 ADNI samples. The first and second PCs for the 757 samples were provided. Ethnic group label for each individual is taken from PTRACCAT object in ADNIMERGE package: American Indian or Alaskan Native (Am Indian/Alaskan), Asian (Asian), Hawaiian/Other PI (Hawaiian or Other Pacific Islander), Black or African American (Black), White (White), More than One Reported (More than one), Unknown or Not Reported (Unknown).
(EPS)

S2 Fig Scatter plots of the phenotype–environment pairs showing *l*_*approx*_ > 1.5 from five phenotypes and 117 metabolite variables. *l*_*approx*_ > 1.5 was computed for the ADNI 684 non-Hispanic Caucasian samples. Phenotypes: HEIGHT (height, cm), BMI (body mass index, *kg*/*m*^2^), WholeBrain (whole-brain volume, *cm*^3^), and MMSE (mini mental state examination, score); Metabolites as environment variables: C10.2, C10.2, C4, C5, PC.aa.C42.6, PC.ae.C38.2, PC.ae.C40.3, PC.ae.C44.3, C10.2, C5.DC‥C6.OH., C5.DC‥C6.OH.
(EPS)

S3 Fig Comparison between *l*_*approx*_ and *l*_*mean*_ for joint GxE interaction and marginal association tests on ADNI-GWAS data for 684 non-Hispanic Caucasian samples with simulated genotype data. Joint GxE interaction and marginal association tests are carried out on ADNI-GWAS data for 684 non-Hispanic Caucasian samples where only the real genotype data was replaced by simulation with 10000 loci independently generated under linkage equilibrium, where MAFs are set by uniform distribution on [0.05, 0.5] (ADNI684sim) Phenotypes, covariates and environment variables in real data are fixed. Five phenotypes—height (HEIGHT), body mass index (BMI), whole brain (WholeBrain), intracranial volume (ICV), and mini mental state exam (MMSE)—and nine environment variables: lysoPC.a.C16.0, PC.ae.C38.2, PC.ae.C40.3, C10, PC.aa.C40.5, PC.ae.C36.3, SM‥OH‥C14.1, SM‥OH‥C22.1, and SM.C24.0. Top left: joint GxE interaction test (quantitative phenotype). Top right: marginal association test (quantitative phenotype). Middle left: joint GxE interaction test (quantitative phenotype after Box–Cox transformation optimized in terms of normality). Middle right: joint GxE interaction test (quantitative phenotype after Box–Cox transformation optimized in terms of *l*_*approx*_). Bottom left: joint GxE interaction test (binary phenotype created by dichotomizing quantitative phenotype). Bottom right: marginal association test (binary phenotype created by dichotomizing quantitative phenotype).
(EPS)

S4 Fig Comparison between *l*_*approx*_ and *l*_*mean*_ for joint GxE interaction and marginal association tests on ADNI-GWAS data for 757 samples showing population stratification with adjustment for top 3 and 5 principal components. Comparison between *l*_*approx*_ and *l*_*mean*_, for which population stratification exists, for joint GxE interaction and marginal association tests on ADNI-GWAS data for 757 samples showing population stratification (ADNI757). Five phenotypes—height (HEIGHT), body mass index (BMI), whole brain (WholeBrain), intracranial volume (ICV), and mini mental state exam (MMSE)—and nine metabolite variables: lysoPC.a.C16.0, PC.ae.C38.2, PC.ae.C40.3, C10, PC.aa.C40.5, PC.ae.C36.3, SM‥OH‥C14.1, SM‥OH‥C22.1, and SM.C24.0. Results are shown without and with adjustment for top 10 principal components (PCs). Top left: joint GxE interaction test with adjustment for top 3 PCs (quantitative phenotype). Top right: marginal association test with adjustment for top 3 PCs (quantitative phenotype). Left in the second row: joint GxE interaction test with adjustment for top 5 PCs (quantitative phenotype). Right in the second row: marginal association test with adjustment for top 5 PCs (quantitative phenotype). Left in the third row: joint GxE interaction test with adjustment for top 3 PCs (binary phenotype created by dichotomizing quantitative phenotype). Right in the second row: marginal association test with adjustment for top 3 PCs (binary phenotype created by dichotomizing quantitative phenotype). Bottom left: joint GxE interaction test with adjustment for top 5 PCs (binary phenotype created by dichotomizing quantitative phenotype). Bottom right: marginal association test with adjustment for top 5 PCs (binary phenotype created by dichotomizing quantitative phenotype).
(EPS)

S5 Fig Comparison between *l*_*approx*_ and *l*_*mean*_ for joint GxE interaction and marginal association tests on ADNI-GWAS data for 757 samples with simulated genotype data. Joint GxE interaction and marginal association tests are carried out on ADNI-GWAS data for 757 samples where only the real genotype data, which showed population stratification, was replaced by simulation with 10000 loci independently generated under linkage equilibrium, where MAFs are set by uniform distribution on [0.05, 0.5] (ADNI757sim). Phenotypes, covariates, and environment variables in real data are fixed. Five phenotypes—height (HEIGHT), body mass index (BMI), whole brain (WholeBrain), intracranial volume (ICV), and mini mental state exam (MMSE)—and nine metabolite variables: lysoPC.a.C16.0, PC.ae.C38.2, PC.ae.C40.3, C10, PC.aa.C40.5, PC.ae.C36.3, SM‥OH‥C14.1, SM‥OH‥C22.1, and SM.C24.0. Results are shown with adjustment for top 3 and 5 principal components (PCs). Top left: joint GxE interaction test without PC adjustment (quantitative phenotype). Top right: joint GxE interaction test with adjustment for top 10 PCs (quantitative phenotype). Left in the second row: marginal association test without PC adjustment (quantitative phenotype). Right in the second row: marginal association test with adjustment for top 10 PCs (quantitative phenotype). Left in the third row: joint GxE interaction test without PC adjustment (binary phenotype created by dichotomizing quantitative phenotype). Right in the third row: joint GxE interaction test with adjustment for top 10 PCs (binary phenotype created by dichotomizing quantitative phenotype). Bottom left: marginal association test without PC adjustment (binary phenotype created by dichotomizing quantitative phenotype). Bottom right: marginal association test with adjustment for top 10 PCs (binary phenotype created by dichotomizing quantitative phenotype).
(EPS)

S6 Fig Regression diagnostics plots from linear model fit of BMI on PC.ae.C38.2 in the ADNI data with 684 samples generated by “plot” for “lm” result. (EPS)

S7 Fig Regression diagnostics plots from quadratic model fit of BMI on PC.ae.C40.3 in the ADNI data with 684 samples generated by “plot” for “lm” result. (EPS)

S1 Table *l*_*approx*_ on ADNI-GWAS data for 684 non-Hispanic Caucasian samples. *l*_*approx*_ computed for joint GxE interaction on ADNI-GWAS data for 684 non-Hispanic Caucasian samples, where sex and age are adjusted for, with respect to five quantitative phenotypes, height (HEIGHT), body mass index (BMI), whole brain (WholeBrain), intracranial volume (ICV), and mini mental state exam (MMSE), and 117 metabolite variables as environment variable.
(CSV)

S2 Table Comparison between *l*_*approx*_ and *l*_*mean*_ in quantitative phenotype simulations. Additional quantitative phenotype simulation results.
(PDF)

S3 Table Comparison between *l*_*approx*_ and *l*_*mean*_ in binary phenotype simulations. Additional binary phenotype simulation results.
(PDF)

S1 Appendix Technical details. Details of theoretical results and simulation studies.
(PDF)

S2 Appendix Program code for simulation studies. R code for simulation studies, including a function lapprox to compute *l*_*approx*_ using phenotype, environment and covariate variables as input.
(R) Click here for additional data file.
