# Supplementary material for: Quick assessment for systematic test statistic inflation/deflation due to null model misspecifications in genome-wide environment interaction studies
Source: PLoS One. 2019 Jul 18;14(7):e0219825. doi: 10.1371/journal.pone.0219825 (PMC6638962; doi:10.1371/journal.pone.0219825)
Supplement: S2 Table — Additional quantitative phenotype simulation results. (PDF) [file pone.0219825.s009.pdf]

S2 Table. Comparison between l_{approx} and l_{mean} in quantitative phenotype simulations

Scenario		GxE ($n = 1000$)			Marginal ($n = 1000$)			GxE ($n = 10000$)			Marginal ($n = 10000$)		
(b_G, b_{GE}, b_Z)	M	l_{approx}	l_{mean}	$\frac{l_{approx}}{l_{mean}}$	l_{approx}	l_{mean}	$\frac{l_{approx}}{l_{mean}}$	l_{approx}	l_{mean}	$\frac{l_{approx}}{l_{mean}}$	l_{approx}	l_{mean}	$\frac{l_{approx}}{l_{mean}}$
Base (1,0,0)	r	1.01	1.01	1.00	1.00	1.01	1.00	1.00	1.05	0.95	1.00	1.10	0.91
		(0.03)	(0.04)	(0.02)	(0.00)	(0.03)	(0.03)	(0.01)	(0.03)	(0.02)	(0.00)	(0.04)	(0.03)
Base (1,0,0)	d	1.00	1.04	0.97	1.00	1.07	0.94	1.00	1.21	0.83	1.00	1.42	0.71
		(0.03)	(0.04)	(0.02)	(0.00)	(0.03)	(0.03)	(0.01)	(0.03)	(0.02)	(0.00)	(0.05)	(0.02)
Base (0,0,1)	r	1.15	1.18	0.98	1.00	1.01	1.00	1.03	1.05	0.98	1.00	1.00	1.00
		(0.05)	(0.06)	(0.02)	(0.00)	(0.03)	(0.03)	(0.01)	(0.03)	(0.02)	(0.00)	(0.03)	(0.03)
Base (0,0,1)	d	1.20	1.25	0.96	1.00	1.00	1.00	1.16	1.45	0.80	1.00	1.00	1.00
		(0.05)	(0.06)	(0.02)	(0.00)	(0.03)	(0.03)	(0.02)	(0.04)	(0.02)	(0.00)	(0.03)	(0.03)
1a (1,0,0)	r	1.01	1.02	0.99	1.00	1.02	0.98	1.00	1.16	0.86	1.00	1.32	0.76
		(0.03)	(0.04)	(0.02)	(0.00)	(0.03)	(0.03)	(0.01)	(0.03)	(0.02)	(0.00)	(0.04)	(0.03)
1a (1,0,0)	d	1.01	1.06	0.95	1.00	1.11	0.90	1.00	1.32	0.76	1.00	1.64	0.61
		(0.03)	(0.04)	(0.02)	(0.00)	(0.03)	(0.03)	(0.01)	(0.03)	(0.02)	(0.00)	(0.05)	(0.02)
1a (0,0,1)	r	1.03	1.03	1.00	1.00	1.01	1.00	1.02	1.03	0.99	1.00	1.00	1.00
		(0.04)	(0.04)	(0.02)	(0.00)	(0.03)	(0.03)	(0.01)	(0.03)	(0.02)	(0.00)	(0.03)	(0.03)
1a (0,0,1)	d	1.16	1.19	0.97	1.00	1.00	1.00	1.19	1.70	0.70	1.00	1.00	1.00
		(0.05)	(0.06)	(0.02)	(0.00)	(0.03)	(0.03)	(0.02)	(0.04)	(0.01)	(0.00)	(0.03)	(0.03)
1b (0,0,0)	a	1.00	1.01	1.00	1.00	1.01	1.00	1.00	1.00	1.00	1.00	1.00	1.00
		(0.03)	(0.04)	(0.02)	(0.00)	(0.03)	(0.03)	(0.01)	(0.02)	(0.02)	(0.00)	(0.03)	(0.03)
1b (1,0,0)	a	1.01	1.03	0.98	1.00	1.05	0.96	1.01	1.38	0.73	1.00	1.74	0.57
		(0.03)	(0.04)	(0.02)	(0.00)	(0.03)	(0.03)	(0.01)	(0.03)	(0.02)	(0.00)	(0.06)	(0.02)
1b (1,0,0)	r	1.00	1.03	0.97	1.00	1.06	0.95	1.00	1.01	0.99	1.00	1.02	0.98
		(0.02)	(0.03)	(0.02)	(0.00)	(0.04)	(0.03)	(0.01)	(0.03)	(0.02)	(0.00)	(0.03)	(0.03)
1b (1,0,0)	d	1.00	1.04	0.96	1.00	1.09	0.93	0.99	1.46	0.68	1.00	1.93	0.52
		(0.03)	(0.04)	(0.02)	(0.00)	(0.03)	(0.03)	(0.01)	(0.03)	(0.01)	(0.00)	(0.05)	(0.01)
1b (0,1,0)	a	1.01	1.01	1.00	1.00	1.00	1.00	1.00	1.00	1.00	1.00	1.00	1.00
		(0.03)	(0.04)	(0.02)	(0.00)	(0.03)	(0.03)	(0.01)	(0.02)	(0.02)	(0.00)	(0.03)	(0.03)
1b (0,0,1)	a	1.02	1.05	0.97	1.00	1.05	0.95	1.01	1.13	0.90	1.00	1.21	0.83
		(0.03)	(0.04)	(0.02)	(0.00)	(0.04)	(0.03)	(0.01)	(0.03)	(0.02)	(0.00)	(0.04)	(0.03)
1b (0,0,1)	r	1.01	1.02	1.00	1.00	1.02	0.99	1.01	1.04	0.97	1.00	1.05	0.96
		(0.03)	(0.04)	(0.02)	(0.00)	(0.03)	(0.03)	(0.01)	(0.02)	(0.02)	(0.00)	(0.03)	(0.03)
1b (0,0,1)	d	1.00	1.02	0.99	1.00	1.03	0.98	1.00	1.12	0.90	1.00	1.23	0.82
		(0.03)	(0.04)	(0.02)	(0.00)	(0.03)	(0.03)	(0.01)	(0.02)	(0.02)	(0.00)	(0.04)	(0.03)
1c (0,0,0)	a	1.00	1.01	1.00	1.00	1.01	1.00	1.00	1.00	1.00	1.00	1.00	1.00
		(0.03)	(0.04)	(0.02)	(0.00)	(0.03)	(0.03)	(0.01)	(0.03)	(0.02)	(0.00)	(0.03)	(0.03)
1c (1,0,0)	a	1.01	1.13	0.89	1.00	1.25	0.80	1.00	2.18	0.46	1.00	3.35	0.30
		(0.03)	(0.04)	(0.02)	(0.00)	(0.04)	(0.03)	(0.01)	(0.05)	(0.01)	(0.00)	(0.09)	(0.01)
1c (1,0,0)	r	1.00	1.01	1.00	1.00	1.00	1.00	1.00	1.11	0.90	1.00	1.21	0.82
		(0.03)	(0.04)	(0.02)	(0.00)	(0.03)	(0.03)	(0.01)	(0.03)	(0.02)	(0.00)	(0.04)	(0.03)
1c (1,0,0)	d	1.00	1.05	0.96	1.00	1.10	0.91	1.00	1.32	0.76	1.00	1.64	0.61
		(0.03)	(0.04)	(0.02)	(0.00)	(0.03)	(0.03)	(0.01)	(0.03)	(0.02)	(0.00)	(0.05)	(0.02)
1c (0,1,0)	a	1.00	1.00	1.00	1.00	1.00	1.00	1.00	1.00	1.00	1.00	1.00	1.00
		(0.03)	(0.04)	(0.02)	(0.00)	(0.03)	(0.03)	(0.01)	(0.02)	(0.02)	(0.00)	(0.03)	(0.03)
1c (0,0,1)	a	1.01	1.03	0.98	1.00	1.05	0.96	1.00	1.14	0.88	1.00	1.29	0.78
		(0.03)	(0.04)	(0.02)	(0.00)	(0.03)	(0.03)	(0.01)	(0.03)	(0.02)	(0.00)	(0.04)	(0.02)
1c (0,0,1)	r	1.01	1.01	1.00	1.00	1.01	1.00	1.00	1.00	1.00	1.00	1.00	1.00
		(0.03)	(0.04)	(0.02)	(0.00)	(0.03)	(0.03)	(0.01)	(0.03)	(0.02)	(0.00)	(0.03)	(0.03)
1c (0,0,1)	d	1.01	1.02	0.99	1.00	1.02	0.98	1.00	1.14	0.87	1.00	1.29	0.78
		(0.03)	(0.04)	(0.02)	(0.00)	(0.03)	(0.03)	(0.01)	(0.02)	(0.02)	(0.00)	(0.04)	(0.02)

Mean and standard deviation (SD) for l_{approx}, l_{mean} and the ratio l_{approx}/l_{mean} are shown over 200 simulation runs for gene-environment (GxE) interaction and marginal association analyses for two sample sizes, $n = 1000$ and 10000. M, genotype model (a: additive, r: recessive, d: dominant). b_G, b_{GE} and b_Z are effect size parameters of genotype, gene-environment interaction and covariate, respectively; 0 and 1 denote absence and presence of effect.

S2 Table (continued). Comparison between l_{approx} and l_{mean} in quantitative phenotype simulations

Scenario			GxE ($n = 1000$)			Marginal ($n = 1000$)			GxE ($n = 10000$)			Marginal ($n = 10000$)		
	(b_G, b_{GE}, b_Z)	M	l_{approx}	l_{mean}	$\frac{l_{approx}}{l_{mean}}$	l_{approx}	l_{mean}	$\frac{l_{approx}}{l_{mean}}$	l_{approx}	l_{mean}	$\frac{l_{approx}}{l_{mean}}$	l_{approx}	l_{mean}	$\frac{l_{approx}}{l_{mean}}$
1d	(0,0,0)	a	1.00	1.00	1.00	1.00	1.01	1.00	1.00	1.00	1.00	1.00	1.00	1.00
			(0.03)	(0.04)	(0.02)	(0.00)	(0.03)	(0.03)	(0.01)	(0.02)	(0.02)	(0.00)	(0.03)	(0.03)
1d	(1,0,0)	a	1.01	1.10	0.92	1.00	1.18	0.85	1.00	2.24	0.45	1.00	3.48	0.29
			(0.03)	(0.05)	(0.02)	(0.00)	(0.04)	(0.03)	(0.01)	(0.05)	(0.01)	(0.00)	(0.09)	(0.01)
1d	(1,0,0)	r	1.01	1.02	0.99	1.00	1.02	0.98	1.00	1.25	0.80	1.00	1.49	0.67
			(0.03)	(0.03)	(0.02)	(0.00)	(0.03)	(0.03)	(0.01)	(0.03)	(0.02)	(0.00)	(0.05)	(0.02)
1d	(1,0,0)	d	1.01	1.04	0.96	1.00	1.08	0.93	1.00	1.51	0.66	1.00	2.01	0.50
			(0.03)	(0.04)	(0.02)	(0.00)	(0.03)	(0.03)	(0.01)	(0.03)	(0.01)	(0.00)	(0.06)	(0.01)
1d	(0,1,0)	a	1.01	1.01	1.00	1.00	1.01	1.00	1.00	1.00	1.00	1.00	1.00	1.00
			(0.03)	(0.04)	(0.02)	(0.00)	(0.03)	(0.03)	(0.01)	(0.02)	(0.02)	(0.00)	(0.03)	(0.03)
1d	(0,0,1)	a	1.05	1.07	0.99	1.00	1.01	1.00	1.33	2.54	0.52	1.00	1.00	1.00
			(0.04)	(0.05)	(0.02)	(0.00)	(0.03)	(0.03)	(0.02)	(0.06)	(0.01)	(0.00)	(0.03)	(0.03)
1d	(0,0,1)	r	1.16	1.18	0.98	1.00	1.01	1.00	1.00	1.00	1.00	1.00	1.00	1.00
			(0.05)	(0.06)	(0.02)	(0.00)	(0.04)	(0.03)	(0.01)	(0.02)	(0.02)	(0.00)	(0.03)	(0.03)
1d	(0,0,1)	d	1.21	1.26	0.96	1.00	1.01	1.00	1.20	1.69	0.71	1.00	1.00	1.00
			(0.05)	(0.06)	(0.02)	(0.00)	(0.03)	(0.03)	(0.02)	(0.04)	(0.01)	(0.00)	(0.03)	(0.03)
2a	(1,0,0)	r	1.01	1.04	0.97	1.00	1.06	0.95	1.00	1.19	0.84	1.00	1.38	0.73
			(0.03)	(0.04)	(0.02)	(0.00)	(0.03)	(0.03)	(0.01)	(0.03)	(0.02)	(0.00)	(0.04)	(0.02)
2a	(1,0,0)	d	1.00	1.05	0.96	1.00	1.10	0.92	1.00	1.49	0.67	1.00	1.99	0.50
			(0.03)	(0.04)	(0.02)	(0.00)	(0.04)	(0.03)	(0.01)	(0.03)	(0.01)	(0.00)	(0.06)	(0.01)
2a	(0,0,1)	r	1.11	1.12	0.99	1.00	1.00	1.00	1.01	1.02	1.00	1.00	1.00	1.00
			(0.05)	(0.06)	(0.02)	(0.00)	(0.03)	(0.03)	(0.01)	(0.03)	(0.02)	(0.00)	(0.03)	(0.03)
2a	(0,0,1)	d	1.09	1.12	0.98	1.00	1.00	1.00	1.20	1.71	0.70	1.00	1.00	1.01
			(0.05)	(0.06)	(0.02)	(0.00)	(0.03)	(0.03)	(0.01)	(0.04)	(0.01)	(0.00)	(0.03)	(0.03)
2b	(0,0,0)	a	1.01	1.01	1.00	1.00	1.00	1.00	1.00	1.00	1.00	1.00	1.01	1.00
			(0.03)	(0.04)	(0.02)	(0.00)	(0.03)	(0.03)	(0.01)	(0.03)	(0.02)	(0.00)	(0.03)	(0.03)
2b	(1,0,0)	a	1.00	1.03	0.97	1.00	1.05	0.95	1.00	1.11	0.90	1.00	1.23	0.82
			(0.03)	(0.04)	(0.02)	(0.00)	(0.03)	(0.03)	(0.01)	(0.03)	(0.02)	(0.00)	(0.04)	(0.02)
2b	(1,0,0)	r	1.01	1.01	0.99	1.00	1.01	0.99	1.00	1.00	1.00	1.00	1.00	1.00
			(0.03)	(0.04)	(0.02)	(0.00)	(0.03)	(0.03)	(0.01)	(0.02)	(0.02)	(0.00)	(0.03)	(0.03)
2b	(1,0,0)	d	1.00	1.05	0.96	1.00	1.08	0.93	1.00	1.32	0.76	1.00	1.64	0.61
			(0.03)	(0.04)	(0.02)	(0.00)	(0.04)	(0.03)	(0.01)	(0.03)	(0.02)	(0.00)	(0.05)	(0.02)
2b	(0,1,0)	a	1.00	1.01	1.00	1.00	1.01	0.99	1.00	1.00	1.00	1.00	1.00	1.00
			(0.03)	(0.04)	(0.02)	(0.00)	(0.03)	(0.03)	(0.01)	(0.03)	(0.02)	(0.00)	(0.03)	(0.03)
2b	(0,0,1)	a	1.00	1.04	0.97	1.00	1.07	0.94	1.00	1.34	0.74	1.00	1.69	0.59
			(0.03)	(0.03)	(0.02)	(0.00)	(0.03)	(0.03)	(0.01)	(0.03)	(0.02)	(0.00)	(0.05)	(0.02)
2b	(0,0,1)	r	1.01	1.01	1.00	1.00	1.01	1.00	1.00	1.05	0.95	1.00	1.09	0.92
			(0.03)	(0.04)	(0.02)	(0.00)	(0.03)	(0.03)	(0.01)	(0.02)	(0.02)	(0.00)	(0.04)	(0.03)
2b	(0,0,1)	d	1.00	1.02	0.98	1.00	1.04	0.97	1.00	1.11	0.90	1.00	1.22	0.82
			(0.03)	(0.04)	(0.02)	(0.00)	(0.03)	(0.03)	(0.01)	(0.03)	(0.02)	(0.00)	(0.04)	(0.03)
2c	(1,0,0)	r	2.28	2.28	1.00	1.00	1.01	0.99	2.31	2.33	0.99	1.00	1.04	0.96
			(0.28)	(0.27)	(0.02)	(0.00)	(0.03)	(0.03)	(0.08)	(0.10)	(0.02)	(0.00)	(0.03)	(0.03)
2c	(1,0,0)	d	2.24	2.26	0.99	1.00	1.03	0.98	2.22	2.39	0.93	1.00	1.34	0.75
			(0.30)	(0.30)	(0.03)	(0.00)	(0.03)	(0.03)	(0.08)	(0.10)	(0.02)	(0.00)	(0.04)	(0.02)
2c	(0,0,1)	r	2.31	2.32	1.00	1.00	1.00	1.00	2.32	2.33	1.00	1.00	1.00	1.00
			(0.24)	(0.25)	(0.02)	(0.00)	(0.03)	(0.03)	(0.08)	(0.10)	(0.03)	(0.00)	(0.03)	(0.03)
2c	(0,0,1)	d	2.33	2.33	1.00	1.00	1.01	1.00	2.31	2.49	0.93	1.00	1.00	1.00
			(0.28)	(0.27)	(0.03)	(0.00)	(0.03)	(0.03)	(0.10)	(0.12)	(0.02)	(0.00)	(0.03)	(0.03)

Mean and standard deviation (SD) for l_{approx}, l_{mean} and the ratio l_{approx}/l_{mean} are shown over 200 simulation runs for gene-environment (GxE) interaction and marginal association analyses for two sample sizes, $n = 1000$ and 10000. M, genotype model (a: additive, r: recessive, d: dominant). b_G, b_{GE} and b_Z are effect size parameters of genotype, gene-environment interaction and covariate, respectively; 0 and 1 denote absence and presence of effect.

S2 Table (continued). Comparison between l_{approx} and l_{mean} in quantitative phenotype simulations

Scenario			GxE ($n = 1000$)			Marginal ($n = 1000$)			GxE ($n = 10000$)			Marginal ($n = 10000$)		
	(b_G, b_{GE}, b_Z)	M	l_{approx}	l_{mean}	$\frac{l_{approx}}{l_{mean}}$	l_{approx}	l_{mean}	$\frac{l_{approx}}{l_{mean}}$	l_{approx}	l_{mean}	$\frac{l_{approx}}{l_{mean}}$	l_{approx}	l_{mean}	$\frac{l_{approx}}{l_{mean}}$
2d	(1,0,0)	r	0.94	0.98	0.95	1.00	1.04	0.96	0.99	1.01	0.97	1.00	1.03	0.97
			(0.26)	(0.18)	(0.07)	(0.00)	(0.03)	(0.03)	(0.21)	(0.16)	(0.05)	(0.00)	(0.03)	(0.03)
2d	(1,0,0)	d	1.00	1.05	0.94	1.00	1.08	0.93	0.99	1.49	0.66	1.00	1.99	0.50
			(0.33)	(0.24)	(0.08)	(0.00)	(0.03)	(0.03)	(0.22)	(0.16)	(0.06)	(0.00)	(0.06)	(0.01)
2d	(0,0,1)	r	1.45	1.33	1.05	1.00	1.00	1.00	10.77	8.19	1.31	1.00	1.00	1.00
			(0.79)	(0.56)	(0.11)	(0.00)	(0.03)	(0.03)	(2.81)	(2.07)	(0.03)	(0.00)	(0.03)	(0.03)
2d	(0,0,1)	d	13.70	10.08	1.36	1.00	1.01	1.00	105.30	77.70	1.36	1.00	1.00	1.00
			(3.61)	(2.55)	(0.03)	(0.00)	(0.03)	(0.03)	(8.54)	(6.50)	(0.03)	(0.00)	(0.03)	(0.03)
2e	(0,0,0)	a	1.01	1.01	1.00	1.00	1.01	1.00	1.02	1.02	1.00	1.00	1.00	1.00
			(0.21)	(0.20)	(0.02)	(0.00)	(0.03)	(0.03)	(0.23)	(0.21)	(0.03)	(0.00)	(0.03)	(0.03)
2e	(1,0,0)	a	0.98	1.05	0.93	1.00	1.13	0.89	1.01	2.02	0.50	1.00	3.02	0.33
			(0.22)	(0.21)	(0.04)	(0.00)	(0.04)	(0.03)	(0.20)	(0.20)	(0.05)	(0.00)	(0.09)	(0.01)
2e	(1,0,0)	r	1.02	1.04	0.97	1.00	1.05	0.96	0.99	1.22	0.80	1.00	1.47	0.68
			(0.25)	(0.24)	(0.03)	(0.00)	(0.03)	(0.03)	(0.21)	(0.19)	(0.05)	(0.00)	(0.04)	(0.02)
2e	(1,0,0)	d	1.03	1.06	0.97	1.00	1.07	0.94	0.99	1.38	0.71	1.00	1.79	0.56
			(0.25)	(0.23)	(0.03)	(0.00)	(0.03)	(0.03)	(0.21)	(0.19)	(0.05)	(0.00)	(0.05)	(0.02)
2e	(0,1,0)	a	1.12	1.91	0.58	1.00	1.01	1.00	9.48	16.61	0.57	1.00	1.00	1.00
			(0.25)	(0.27)	(0.06)	(0.00)	(0.03)	(0.03)	(0.43)	(0.96)	(0.02)	(0.00)	(0.03)	(0.03)
2e	(0,0,1)	a	1.19	2.59	0.46	1.00	1.00	1.00	2.65	4.10	0.65	1.00	1.00	1.00
			(0.26)	(0.32)	(0.06)	(0.00)	(0.03)	(0.03)	(0.29)	(0.47)	(0.04)	(0.00)	(0.03)	(0.03)
2e	(0,0,1)	r	1.00	1.01	0.99	1.00	1.01	1.00	1.01	1.02	0.99	1.00	1.00	1.00
			(0.25)	(0.23)	(0.03)	(0.00)	(0.03)	(0.03)	(0.20)	(0.19)	(0.03)	(0.00)	(0.03)	(0.03)
2e	(0,0,1)	d	1.04	1.07	0.97	1.00	1.01	1.00	4.78	7.86	0.61	1.00	1.00	1.00
			(0.25)	(0.23)	(0.03)	(0.00)	(0.03)	(0.03)	(0.37)	(0.59)	(0.03)	(0.00)	(0.03)	(0.03)
3a	(0,0,0)	a	1.01	1.01	1.00	1.01	1.01	1.00	1.00	1.00	1.00	1.00	1.00	1.00
			(0.03)	(0.03)	(0.02)	(0.00)	(0.03)	(0.03)	(0.01)	(0.02)	(0.02)	(0.00)	(0.03)	(0.03)
3a	(1,0,0)	a	1.01	1.09	0.92	1.01	1.17	0.86	1.00	1.92	0.52	1.00	2.83	0.35
			(0.04)	(0.04)	(0.02)	(0.00)	(0.04)	(0.03)	(0.01)	(0.04)	(0.01)	(0.00)	(0.08)	(0.01)
3a	(1,0,0)	r	1.01	1.01	1.00	1.01	1.01	1.00	1.00	1.01	0.99	1.00	1.01	0.99
			(0.03)	(0.04)	(0.02)	(0.00)	(0.03)	(0.03)	(0.01)	(0.02)	(0.02)	(0.00)	(0.03)	(0.03)
3a	(1,0,0)	d	1.01	1.03	0.98	1.01	1.05	0.96	1.00	1.50	0.67	1.00	2.00	0.50
			(0.03)	(0.04)	(0.02)	(0.00)	(0.03)	(0.03)	(0.01)	(0.03)	(0.01)	(0.00)	(0.06)	(0.01)
3a	(0,1,0)	a	1.01	1.01	1.00	1.01	1.01	1.00	1.00	1.00	1.00	1.00	1.00	1.00
			(0.03)	(0.04)	(0.02)	(0.00)	(0.03)	(0.03)	(0.01)	(0.02)	(0.02)	(0.00)	(0.03)	(0.03)
3a	(0,0,1)	a	1.21	1.27	0.95	1.01	1.01	1.00	1.11	1.41	0.79	1.00	1.00	1.00
			(0.07)	(0.08)	(0.02)	(0.00)	(0.03)	(0.03)	(0.02)	(0.04)	(0.02)	(0.00)	(0.03)	(0.03)
3a	(0,0,1)	r	1.04	1.04	1.00	1.01	1.01	1.00	1.01	1.01	1.00	1.00	1.00	1.00
			(0.04)	(0.04)	(0.02)	(0.00)	(0.03)	(0.03)	(0.01)	(0.03)	(0.02)	(0.00)	(0.03)	(0.03)
3a	(0,0,1)	d	1.21	1.26	0.96	1.01	1.01	1.00	1.18	1.56	0.76	1.00	1.00	1.00
			(0.05)	(0.06)	(0.02)	(0.00)	(0.03)	(0.03)	(0.02)	(0.04)	(0.02)	(0.00)	(0.03)	(0.03)

Mean and standard deviation (SD) for l_{approx}, l_{mean} and the ratio l_{approx}/l_{mean} are shown over 200 simulation runs for gene-environment (GxE) interaction and marginal association analyses for two sample sizes, $n = 1000$ and 10000. M, genotype model (a: additive, r: recessive, d: dominant). b_G, b_{GE} and b_Z are effect size parameters of genotype, gene-environment interaction and covariate, respectively; 0 and 1 denote absence and presence of effect.

S2 Table (continued). Comparison between l_{approx} and l_{mean} in quantitative phenotype simulations

Scenario			GxE ($n = 1000$)			Marginal ($n = 1000$)			GxE ($n = 10000$)			Marginal ($n = 10000$)		
	(b_G, b_{GE}, b_Z)	M	l_{approx}	l_{mean}	$\frac{l_{approx}}{l_{mean}}$	l_{approx}	l_{mean}	$\frac{l_{approx}}{l_{mean}}$	l_{approx}	l_{mean}	$\frac{l_{approx}}{l_{mean}}$	l_{approx}	l_{mean}	$\frac{l_{approx}}{l_{mean}}$
3b	(0,0,0)	a	1.00	1.01	1.00	1.00	1.00	1.00	1.00	1.00	1.00	1.00	1.00	1.00
			(0.02)	(0.03)	(0.02)	(0.00)	(0.03)	(0.03)	(0.01)	(0.02)	(0.02)	(0.00)	(0.03)	(0.03)
3b	(1,0,0)	a	1.00	1.12	0.90	1.00	1.23	0.81	1.00	2.22	0.45	1.00	3.44	0.29
			(0.02)	(0.03)	(0.02)	(0.00)	(0.04)	(0.03)	(0.01)	(0.05)	(0.01)	(0.00)	(0.09)	(0.01)
3b	(1,0,0)	r	1.00	1.00	1.00	1.00	1.01	1.00	1.00	1.10	0.91	1.00	1.20	0.83
			(0.02)	(0.03)	(0.02)	(0.00)	(0.03)	(0.03)	(0.01)	(0.02)	(0.02)	(0.00)	(0.04)	(0.03)
3b	(1,0,0)	d	1.00	1.04	0.96	1.00	1.08	0.93	1.00	1.42	0.70	1.00	1.84	0.54
			(0.02)	(0.03)	(0.02)	(0.00)	(0.03)	(0.03)	(0.01)	(0.03)	(0.02)	(0.00)	(0.06)	(0.02)
3b	(0,1,0)	a	1.00	1.01	1.00	1.00	1.01	1.00	1.00	1.00	1.00	1.00	1.00	1.00
			(0.02)	(0.03)	(0.02)	(0.00)	(0.03)	(0.03)	(0.01)	(0.02)	(0.02)	(0.00)	(0.03)	(0.03)
3b	(0,0,1)	a	1.08	1.81	0.60	1.00	1.63	0.62	1.04	2.96	0.35	1.00	3.41	0.29
			(0.03)	(0.08)	(0.02)	(0.00)	(0.06)	(0.02)	(0.01)	(0.11)	(0.01)	(0.00)	(0.14)	(0.01)
3b	(0,0,1)	r	1.01	1.02	0.99	1.00	1.01	0.99	1.05	2.04	0.52	1.00	2.35	0.43
			(0.02)	(0.03)	(0.02)	(0.00)	(0.03)	(0.03)	(0.01)	(0.06)	(0.01)	(0.00)	(0.07)	(0.01)
3b	(0,0,1)	d	1.07	1.27	0.84	1.00	1.26	0.80	1.06	3.36	0.32	1.00	3.80	0.26
			(0.02)	(0.04)	(0.02)	(0.00)	(0.04)	(0.03)	(0.01)	(0.06)	(0.01)	(0.00)	(0.09)	(0.01)
3c	(0,0,0)	a	1.01	1.01	1.00	1.00	1.00	1.00	1.00	1.00	1.00	1.00	0.99	1.01
			(0.00)	(0.02)	(0.02)	(0.00)	(0.03)	(0.03)	(0.00)	(0.02)	(0.02)	(0.00)	(0.03)	(0.03)
3c	(1,0,0)	a	1.01	1.13	0.89	1.00	1.25	0.80	1.00	1.54	0.65	1.00	2.09	0.48
			(0.00)	(0.02)	(0.02)	(0.00)	(0.04)	(0.03)	(0.00)	(0.04)	(0.02)	(0.00)	(0.06)	(0.01)
3c	(1,0,0)	r	1.01	1.01	1.00	1.00	1.01	1.00	1.00	1.00	1.00	1.00	1.00	1.00
			(0.00)	(0.02)	(0.02)	(0.00)	(0.03)	(0.03)	(0.00)	(0.02)	(0.02)	(0.00)	(0.03)	(0.03)
3c	(1,0,0)	d	1.01	1.03	0.98	1.00	1.05	0.96	1.00	1.48	0.68	1.00	1.96	0.51
			(0.00)	(0.02)	(0.02)	(0.00)	(0.03)	(0.03)	(0.00)	(0.03)	(0.01)	(0.00)	(0.06)	(0.02)
3c	(0,1,0)	a	1.01	1.01	1.00	1.00	1.01	1.00	1.00	1.00	1.00	1.00	1.00	1.00
			(0.00)	(0.02)	(0.02)	(0.00)	(0.03)	(0.03)	(0.00)	(0.03)	(0.03)	(0.00)	(0.03)	(0.03)
3c	(0,0,1)	a	1.01	1.07	0.94	1.00	1.06	0.95	1.00	1.62	0.62	1.00	1.55	0.64
			(0.01)	(0.03)	(0.02)	(0.00)	(0.04)	(0.03)	(0.00)	(0.04)	(0.01)	(0.00)	(0.05)	(0.02)
3c	(0,0,1)	r	1.01	1.01	0.99	1.00	1.01	1.00	1.00	1.01	1.00	1.00	1.00	1.00
			(0.00)	(0.02)	(0.02)	(0.00)	(0.03)	(0.03)	(0.00)	(0.02)	(0.02)	(0.00)	(0.03)	(0.03)
3c	(0,0,1)	d	1.01	1.03	0.98	1.00	1.03	0.98	1.00	1.23	0.82	1.00	1.22	0.82
			(0.00)	(0.02)	(0.02)	(0.00)	(0.03)	(0.03)	(0.00)	(0.03)	(0.02)	(0.00)	(0.04)	(0.03)
3d	(0,0,0)	a	1.01	1.01	1.00	1.00	1.01	1.00	1.00	1.00	1.00	1.00	1.00	1.00
			(0.02)	(0.03)	(0.02)	(0.00)	(0.03)	(0.03)	(0.01)	(0.02)	(0.02)	(0.00)	(0.03)	(0.03)
3d	(1,0,0)	a	1.01	1.05	0.96	1.00	1.09	0.92	1.00	2.16	0.46	1.00	3.33	0.30
			(0.03)	(0.04)	(0.02)	(0.00)	(0.03)	(0.03)	(0.01)	(0.05)	(0.01)	(0.00)	(0.09)	(0.01)
3d	(1,0,0)	r	1.00	1.03	0.98	1.00	1.05	0.96	1.00	1.15	0.87	1.00	1.29	0.78
			(0.03)	(0.04)	(0.02)	(0.00)	(0.03)	(0.03)	(0.01)	(0.03)	(0.02)	(0.00)	(0.04)	(0.02)
3d	(1,0,0)	d	1.00	1.03	0.98	1.00	1.05	0.95	1.00	1.50	0.67	1.00	2.00	0.50
			(0.02)	(0.03)	(0.02)	(0.00)	(0.03)	(0.03)	(0.01)	(0.03)	(0.01)	(0.00)	(0.05)	(0.01)
3d	(0,1,0)	a	1.00	1.01	1.00	1.00	1.01	1.00	1.00	1.00	1.00	1.00	1.00	1.01
			(0.03)	(0.03)	(0.02)	(0.00)	(0.03)	(0.03)	(0.01)	(0.03)	(0.02)	(0.00)	(0.03)	(0.03)
3d	(0,0,1)	a	1.10	1.42	0.77	1.00	1.37	0.74	1.09	4.20	0.26	1.00	4.55	0.22
			(0.03)	(0.06)	(0.02)	(0.00)	(0.05)	(0.03)	(0.01)	(0.10)	(0.01)	(0.00)	(0.12)	(0.01)
3d	(0,0,1)	r	1.06	1.14	0.94	1.00	1.11	0.91	1.01	1.04	0.97	1.00	1.04	0.96
			(0.03)	(0.04)	(0.02)	(0.00)	(0.03)	(0.03)	(0.01)	(0.02)	(0.02)	(0.00)	(0.03)	(0.03)
3d	(0,0,1)	d	1.07	1.18	0.91	1.00	1.15	0.88	1.07	2.23	0.48	1.00	2.57	0.39
			(0.03)	(0.04)	(0.02)	(0.00)	(0.04)	(0.03)	(0.01)	(0.05)	(0.01)	(0.00)	(0.08)	(0.01)

Mean and standard deviation (SD) for l_{approx}, l_{mean} and the ratio l_{approx}/l_{mean} are shown over 200 simulation runs for gene-environment (GxE) interaction and marginal association analyses for two sample sizes, $n = 1000$ and 10000. M, genotype model (a: additive, r: recessive, d: dominant). b_G, b_{GE} and b_Z are effect size parameters of genotype, gene-environment interaction and covariate, respectively; 0 and 1 denote absence and presence of effect.

S2 Table (continued). Comparison between l_{approx} and l_{mean} in quantitative phenotype simulations

Scenario			GxE ($n = 1000$)			Marginal ($n = 1000$)			GxE ($n = 10000$)			Marginal ($n = 10000$)		
(b_G, b_{GE}, b_Z)		M	l_{approx}	l_{mean}	$\frac{l_{approx}}{l_{mean}}$	l_{approx}	l_{mean}	$\frac{l_{approx}}{l_{mean}}$	l_{approx}	l_{mean}	$\frac{l_{approx}}{l_{mean}}$	l_{approx}	l_{mean}	$\frac{l_{approx}}{l_{mean}}$
4a	(0,0,0)	a	1.01 (0.03)	1.02 (0.07)	0.99 (0.06)	1.00 (0.00)	1.02 (0.09)	0.99 (0.09)	1.00 (0.01)	1.02 (0.06)	0.99 (0.06)	1.00 (0.00)	1.02 (0.09)	0.99 (0.09)
4a	(1,0,0)	a	1.01 (0.03)	1.48 (0.13)	0.69 (0.06)	1.00 (0.00)	1.92 (0.22)	0.53 (0.06)	1.00 (0.01)	5.04 (0.34)	0.20 (0.01)	1.00 (0.00)	9.08 (0.68)	0.11 (0.01)
4a	(1,0,0)	r	1.00 (0.03)	1.08 (0.09)	0.93 (0.07)	1.00 (0.00)	1.16 (0.13)	0.88 (0.10)	1.00 (0.01)	1.02 (0.08)	0.99 (0.07)	1.00 (0.00)	1.05 (0.12)	0.97 (0.10)
4a	(1,0,0)	d	1.01 (0.03)	1.43 (0.12)	0.71 (0.06)	1.00 (0.00)	1.84 (0.23)	0.55 (0.07)	1.00 (0.01)	4.29 (0.32)	0.23 (0.02)	1.00 (0.00)	7.57 (0.63)	0.13 (0.01)
4a	(0,1,0)	a	1.01 (0.03)	1.02 (0.08)	1.00 (0.07)	1.00 (0.00)	1.02 (0.10)	0.99 (0.09)	1.00 (0.01)	1.00 (0.06)	1.01 (0.06)	1.00 (0.00)	1.00 (0.09)	1.01 (0.09)
4a	(0,0,1)	a	1.06 (0.05)	1.13 (0.10)	0.94 (0.07)	1.00 (0.00)	1.00 (0.09)	1.01 (0.09)	1.17 (0.02)	4.42 (0.34)	0.27 (0.02)	1.00 (0.00)	1.00 (0.09)	1.01 (0.09)
4a	(0,0,1)	r	1.12 (0.05)	1.28 (0.12)	0.88 (0.06)	1.00 (0.00)	1.02 (0.09)	1.00 (0.09)	1.05 (0.01)	1.46 (0.13)	0.72 (0.07)	1.00 (0.00)	1.01 (0.10)	1.00 (0.09)
4a	(0,0,1)	d	1.16 (0.05)	1.48 (0.15)	0.80 (0.07)	1.00 (0.00)	1.01 (0.09)	1.00 (0.09)	1.18 (0.02)	4.57 (0.34)	0.26 (0.02)	1.00 (0.00)	1.00 (0.09)	1.00 (0.09)
4b	(0,0,0)	a	1.00 (0.03)	1.00 (0.04)	1.00 (0.02)	1.00 (0.00)	1.00 (0.03)	1.00 (0.03)	1.00 (0.01)	1.00 (0.02)	1.00 (0.02)	1.00 (0.00)	1.00 (0.03)	1.00 (0.03)
4b	(1,0,0)	a	1.00 (0.03)	1.03 (0.04)	0.98 (0.02)	1.00 (0.00)	1.05 (0.03)	0.96 (0.03)	1.00 (0.01)	2.24 (0.05)	0.45 (0.01)	1.00 (0.00)	3.49 (0.08)	0.29 (0.01)
4b	(1,0,0)	r	1.00 (0.03)	1.00 (0.04)	1.00 (0.02)	1.00 (0.00)	1.00 (0.03)	1.00 (0.03)	1.00 (0.01)	1.00 (0.02)	1.00 (0.02)	1.00 (0.00)	1.00 (0.03)	1.00 (0.03)
4b	(1,0,0)	d	1.00 (0.03)	1.04 (0.04)	0.97 (0.02)	1.00 (0.00)	1.06 (0.03)	0.94 (0.03)	1.00 (0.01)	1.10 (0.03)	0.91 (0.02)	1.00 (0.00)	1.18 (0.04)	0.85 (0.03)
4b	(0,1,0)	a	1.01 (0.03)	1.01 (0.04)	1.00 (0.02)	1.00 (0.00)	1.01 (0.03)	1.00 (0.03)	1.00 (0.01)	1.00 (0.03)	1.00 (0.02)	1.00 (0.00)	1.00 (0.03)	1.00 (0.03)
4b	(0,0,1)	a	1.04 (0.04)	1.05 (0.05)	0.99 (0.02)	1.00 (0.00)	1.01 (0.03)	1.00 (0.03)	1.26 (0.02)	2.12 (0.06)	0.59 (0.01)	1.00 (0.00)	1.00 (0.03)	1.00 (0.03)
4b	(0,0,1)	r	1.10 (0.05)	1.11 (0.06)	0.99 (0.02)	1.00 (0.00)	1.01 (0.03)	0.99 (0.03)	1.01 (0.01)	1.02 (0.03)	0.99 (0.02)	1.00 (0.00)	1.00 (0.03)	1.00 (0.03)
4b	(0,0,1)	d	1.04 (0.04)	1.05 (0.04)	0.99 (0.02)	1.00 (0.00)	1.01 (0.03)	1.00 (0.03)	1.12 (0.02)	1.44 (0.04)	0.78 (0.02)	1.00 (0.00)	1.00 (0.03)	1.00 (0.03)
4c	(0,0,0)	a	1.00 (0.03)	1.00 (0.07)	1.01 (0.06)	1.00 (0.00)	1.00 (0.08)	1.01 (0.08)	1.00 (0.01)	1.00 (0.06)	1.00 (0.05)	1.00 (0.00)	1.00 (0.08)	1.00 (0.08)
4c	(1,0,0)	a	1.01 (0.03)	1.47 (0.12)	0.69 (0.05)	1.00 (0.00)	1.92 (0.21)	0.53 (0.06)	1.00 (0.01)	2.09 (0.17)	0.48 (0.04)	1.00 (0.00)	3.18 (0.33)	0.32 (0.03)
4c	(1,0,0)	r	1.01 (0.03)	1.20 (0.09)	0.84 (0.05)	1.00 (0.00)	1.40 (0.14)	0.73 (0.07)	1.00 (0.01)	1.00 (0.06)	1.00 (0.06)	1.00 (0.00)	1.00 (0.08)	1.01 (0.08)
4c	(1,0,0)	d	1.01 (0.03)	1.21 (0.09)	0.83 (0.06)	1.00 (0.00)	1.42 (0.15)	0.72 (0.07)	1.00 (0.01)	3.66 (0.24)	0.27 (0.02)	1.00 (0.00)	6.32 (0.48)	0.16 (0.01)
4c	(0,1,0)	a	1.00 (0.03)	1.00 (0.07)	1.00 (0.06)	1.00 (0.00)	1.01 (0.08)	1.00 (0.08)	1.00 (0.01)	1.00 (0.06)	1.00 (0.06)	1.00 (0.00)	1.00 (0.08)	1.01 (0.08)
4c	(0,0,1)	a	1.04 (0.04)	1.09 (0.08)	0.96 (0.06)	1.00 (0.00)	1.02 (0.08)	0.99 (0.07)	1.33 (0.02)	8.01 (0.46)	0.17 (0.01)	1.00 (0.00)	1.00 (0.08)	1.01 (0.08)
4c	(0,0,1)	r	1.03 (0.04)	1.05 (0.08)	0.99 (0.06)	1.00 (0.00)	1.01 (0.08)	1.00 (0.08)	1.00 (0.01)	1.00 (0.06)	1.00 (0.06)	1.00 (0.00)	1.00 (0.08)	1.01 (0.08)
4c	(0,0,1)	d	1.09 (0.05)	1.19 (0.10)	0.92 (0.06)	1.00 (0.00)	1.02 (0.08)	1.00 (0.08)	1.07 (0.02)	1.80 (0.15)	0.60 (0.05)	1.00 (0.00)	1.01 (0.08)	1.00 (0.07)

Mean and standard deviation (SD) for l_{approx}, l_{mean} and the ratio l_{approx}/l_{mean} are shown over 200 simulation runs for gene-environment (GxE) interaction and marginal association analyses for two sample sizes, $n = 1000$ and 10000. M, genotype model (a: additive, r: recessive, d: dominant). b_G, b_{GE} and b_Z are effect size parameters of genotype, gene-environment interaction and covariate, respectively; 0 and 1 denote absence and presence of effect.
