# Supplementary material for: Quick assessment for systematic test statistic inflation/deflation due to null model misspecifications in genome-wide environment interaction studies
Source: PLoS One. 2019 Jul 18;14(7):e0219825. doi: 10.1371/journal.pone.0219825 (PMC6638962; doi:10.1371/journal.pone.0219825)
Supplement: S3 Table — Additional binary phenotype simulation results. (PDF) [file pone.0219825.s010.pdf]

S3 Table. Comparison between l_{approx} and l_{mean} in binary phenotype simulations

Scenario			GxE ($n = 1000$)			Marginal ($n = 1000$)			GxE ($n = 10000$)			Marginal ($n = 10000$)		
	(b_G, b_{GE}, b_Z)	M	l_{approx}	l_{mean}	$\frac{l_{approx}}{l_{mean}}$	l_{approx}	l_{mean}	$\frac{l_{approx}}{l_{mean}}$	l_{approx}	l_{mean}	$\frac{l_{approx}}{l_{mean}}$	l_{approx}	l_{mean}	$\frac{l_{approx}}{l_{mean}}$
Base	(1,0,0)	r	1.00	1.00	1.00	1.00	1.00	1.00	1.00	1.00	1.00	1.00	1.00	1.00
			(0.00)	(0.02)	(0.02)	(0.00)	(0.03)	(0.03)	(0.00)	(0.02)	(0.02)	(0.00)	(0.03)	(0.03)
Base	(1,0,0)	d	1.00	1.01	0.99	1.00	1.02	0.98	1.00	1.00	1.00	1.00	1.00	1.00
			(0.02)	(0.03)	(0.02)	(0.00)	(0.03)	(0.03)	(0.00)	(0.02)	(0.02)	(0.00)	(0.03)	(0.03)
Base	(0,0,1)	r	1.00	1.01	1.00	1.00	1.01	1.00	1.00	1.00	1.00	1.00	1.00	1.00
			(0.00)	(0.02)	(0.02)	(0.00)	(0.03)	(0.03)	(0.00)	(0.02)	(0.02)	(0.00)	(0.03)	(0.03)
Base	(0,0,1)	d	1.01	1.02	0.99	1.00	1.00	1.00	1.01	1.01	1.00	1.00	1.00	1.00
			(0.01)	(0.02)	(0.02)	(0.00)	(0.03)	(0.03)	(0.00)	(0.02)	(0.02)	(0.00)	(0.03)	(0.03)
1a	(1,0,0)	r	1.00	1.01	1.00	1.00	1.01	1.00	1.00	1.00	1.00	1.00	1.01	0.99
			(0.00)	(0.03)	(0.02)	(0.00)	(0.04)	(0.04)	(0.00)	(0.02)	(0.02)	(0.00)	(0.04)	(0.04)
1a	(1,0,0)	d	1.01	1.02	0.99	1.00	1.02	0.98	1.00	1.00	1.00	1.00	1.00	1.00
			(0.01)	(0.03)	(0.02)	(0.00)	(0.03)	(0.03)	(0.00)	(0.02)	(0.02)	(0.00)	(0.03)	(0.03)
1a	(0,0,1)	r	1.01	1.01	1.00	1.00	1.00	1.00	1.00	1.00	1.00	1.00	1.00	1.00
			(0.01)	(0.02)	(0.02)	(0.00)	(0.03)	(0.03)	(0.00)	(0.02)	(0.02)	(0.00)	(0.03)	(0.03)
1a	(0,0,1)	d	1.02	1.02	0.99	1.00	1.00	1.00	1.00	1.00	1.00	1.00	1.00	1.00
			(0.02)	(0.03)	(0.02)	(0.00)	(0.03)	(0.03)	(0.00)	(0.02)	(0.02)	(0.00)	(0.03)	(0.03)
1b	(0,0,0)	a	1.00	1.00	1.00	1.00	1.00	1.00	1.00	1.00	1.00	1.00	1.00	1.00
			(0.00)	(0.02)	(0.02)	(0.00)	(0.03)	(0.03)	(0.00)	(0.02)	(0.02)	(0.00)	(0.03)	(0.03)
1b	(1,0,0)	a	1.00	1.02	0.98	1.00	1.03	0.97	1.00	1.00	0.99	1.00	1.01	0.99
			(0.01)	(0.03)	(0.02)	(0.00)	(0.04)	(0.03)	(0.00)	(0.02)	(0.02)	(0.00)	(0.03)	(0.03)
1b	(1,0,0)	r	1.00	1.01	1.00	1.00	1.01	0.99	1.00	1.00	1.00	1.00	1.00	1.00
			(0.01)	(0.02)	(0.02)	(0.00)	(0.03)	(0.03)	(0.00)	(0.02)	(0.02)	(0.00)	(0.03)	(0.03)
1b	(1,0,0)	d	1.01	1.02	0.99	1.00	1.02	0.98	1.00	1.01	0.99	1.00	1.01	0.99
			(0.02)	(0.02)	(0.02)	(0.00)	(0.03)	(0.03)	(0.00)	(0.02)	(0.02)	(0.00)	(0.04)	(0.03)
1b	(0,1,0)	a	1.00	1.01	1.00	1.00	1.00	1.00	1.00	1.00	1.00	1.00	1.00	1.00
			(0.01)	(0.02)	(0.02)	(0.00)	(0.03)	(0.03)	(0.00)	(0.02)	(0.02)	(0.00)	(0.03)	(0.03)
1b	(0,0,1)	a	1.00	1.01	0.99	1.00	1.02	0.98	1.00	1.01	0.99	1.00	1.02	0.99
			(0.01)	(0.03)	(0.02)	(0.00)	(0.03)	(0.03)	(0.00)	(0.02)	(0.02)	(0.00)	(0.03)	(0.03)
1b	(0,0,1)	r	1.00	1.01	1.00	1.00	1.01	1.00	1.00	1.01	0.99	1.00	1.01	0.99
			(0.00)	(0.02)	(0.02)	(0.00)	(0.03)	(0.03)	(0.00)	(0.02)	(0.02)	(0.00)	(0.03)	(0.03)
1b	(0,0,1)	d	1.00	1.01	1.00	1.00	1.01	0.99	1.00	1.00	1.00	1.00	1.01	0.99
			(0.01)	(0.03)	(0.02)	(0.00)	(0.03)	(0.03)	(0.00)	(0.02)	(0.02)	(0.00)	(0.04)	(0.04)
1c	(0,0,0)	a	1.00	1.01	1.00	1.00	1.00	1.00	1.00	1.00	1.00	1.00	1.00	1.00
			(0.00)	(0.02)	(0.02)	(0.00)	(0.03)	(0.03)	(0.00)	(0.02)	(0.02)	(0.00)	(0.03)	(0.03)
1c	(1,0,0)	a	1.00	1.02	0.99	1.00	1.02	0.98	1.00	1.00	1.00	1.00	1.00	1.00
			(0.01)	(0.02)	(0.02)	(0.00)	(0.03)	(0.03)	(0.00)	(0.02)	(0.02)	(0.00)	(0.03)	(0.03)
1c	(1,0,0)	r	1.00	1.00	1.00	1.00	1.00	1.00	1.00	1.00	1.00	1.00	1.00	1.00
			(0.00)	(0.02)	(0.02)	(0.00)	(0.03)	(0.03)	(0.00)	(0.02)	(0.02)	(0.00)	(0.03)	(0.03)
1c	(1,0,0)	d	1.00	1.01	0.99	1.00	1.02	0.98	1.00	1.00	1.00	1.00	1.00	1.00
			(0.01)	(0.03)	(0.02)	(0.00)	(0.03)	(0.03)	(0.01)	(0.02)	(0.02)	(0.00)	(0.03)	(0.03)
1c	(0,1,0)	a	1.00	1.00	1.00	1.00	1.00	1.00	1.00	1.00	1.00	1.00	1.00	1.00
			(0.01)	(0.03)	(0.02)	(0.00)	(0.03)	(0.03)	(0.00)	(0.02)	(0.02)	(0.00)	(0.03)	(0.03)
1c	(0,0,1)	a	1.00	1.01	0.99	1.00	1.02	0.99	1.00	1.00	1.00	1.00	1.00	1.00
			(0.01)	(0.03)	(0.02)	(0.00)	(0.03)	(0.03)	(0.00)	(0.02)	(0.02)	(0.00)	(0.03)	(0.03)
1c	(0,0,1)	r	1.00	1.00	1.00	1.00	1.01	1.00	1.00	1.00	1.00	1.00	1.00	1.00
			(0.00)	(0.02)	(0.02)	(0.00)	(0.03)	(0.03)	(0.00)	(0.02)	(0.02)	(0.00)	(0.03)	(0.03)
1c	(0,0,1)	d	1.00	1.01	1.00	1.00	1.01	0.99	1.00	1.00	1.00	1.00	1.01	0.99
			(0.01)	(0.03)	(0.03)	(0.00)	(0.03)	(0.03)	(0.00)	(0.02)	(0.02)	(0.00)	(0.03)	(0.03)

Mean and standard deviation (SD) for l_{approx}, l_{mean} and the ratio l_{approx}/l_{mean} are shown over 200 simulation runs for gene-environment (GxE) interaction and marginal association analyses for two sample sizes, $n = 1000$ and 10000. M, genotype model (a: additive, r: recessive, d: dominant). b_G, b_{GE} and b_Z are effect size parameters of genotype, gene-environment interaction and covariate, respectively; 0 and 1 denote absence and presence of effect.

S3 Table (continued). Comparison between l_{approx} and l_{mean} in binary phenotype simulations

Scenario			GxE ($n = 1000$)			Marginal ($n = 1000$)			GxE ($n = 10000$)			Marginal ($n = 10000$)		
	(b_G, b_{GE}, b_Z)	M	l_{approx}	l_{mean}	$\frac{l_{approx}}{l_{mean}}$	l_{approx}	l_{mean}	$\frac{l_{approx}}{l_{mean}}$	l_{approx}	l_{mean}	$\frac{l_{approx}}{l_{mean}}$	l_{approx}	l_{mean}	$\frac{l_{approx}}{l_{mean}}$
1d	(0,0,0)	a	1.00	1.01	1.00	1.00	1.01	1.00	1.00	1.00	1.00	1.00	1.00	1.00
			(0.00)	(0.02)	(0.02)	(0.00)	(0.03)	(0.03)	(0.00)	(0.02)	(0.02)	(0.00)	(0.03)	(0.03)
1d	(1,0,0)	a	1.00	1.01	1.00	1.00	1.01	0.99	1.00	1.00	1.00	1.00	1.00	1.00
			(0.00)	(0.02)	(0.02)	(0.00)	(0.03)	(0.03)	(0.00)	(0.02)	(0.02)	(0.00)	(0.03)	(0.03)
1d	(1,0,0)	r	1.00	1.00	1.00	1.00	1.00	1.00	1.00	1.00	1.00	1.00	1.00	1.00
			(0.00)	(0.02)	(0.02)	(0.00)	(0.03)	(0.03)	(0.00)	(0.02)	(0.02)	(0.00)	(0.03)	(0.03)
1d	(1,0,0)	d	1.00	1.01	0.99	1.00	1.02	0.98	1.00	1.00	1.00	1.00	1.00	1.00
			(0.02)	(0.03)	(0.02)	(0.00)	(0.03)	(0.03)	(0.01)	(0.02)	(0.02)	(0.00)	(0.03)	(0.03)
1d	(0,1,0)	a	1.00	1.00	1.00	1.00	0.99	1.01	1.00	1.00	1.00	1.00	1.00	1.00
			(0.02)	(0.03)	(0.02)	(0.00)	(0.03)	(0.03)	(0.01)	(0.02)	(0.02)	(0.00)	(0.03)	(0.03)
1d	(0,0,1)	a	1.02	1.03	0.99	1.00	1.00	1.00	1.01	1.01	1.00	1.00	1.00	1.00
			(0.02)	(0.03)	(0.02)	(0.00)	(0.03)	(0.04)	(0.00)	(0.02)	(0.02)	(0.00)	(0.03)	(0.03)
1d	(0,0,1)	r	1.00	1.00	1.00	1.00	1.00	1.00	1.00	1.00	1.00	1.00	1.00	1.00
			(0.00)	(0.02)	(0.02)	(0.00)	(0.03)	(0.03)	(0.00)	(0.02)	(0.02)	(0.00)	(0.03)	(0.03)
1d	(0,0,1)	d	1.01	1.03	0.99	1.00	1.00	1.00	1.01	1.01	1.00	1.00	1.00	1.00
			(0.02)	(0.03)	(0.02)	(0.00)	(0.03)	(0.03)	(0.01)	(0.02)	(0.02)	(0.00)	(0.03)	(0.03)
2a	(1,0,0)	r	1.00	1.01	1.00	1.00	1.01	1.00	1.00	1.01	0.99	1.00	1.03	0.97
			(0.00)	(0.02)	(0.02)	(0.00)	(0.03)	(0.03)	(0.00)	(0.02)	(0.02)	(0.00)	(0.03)	(0.03)
2a	(1,0,0)	d	1.00	1.01	1.00	1.00	1.01	0.99	1.00	1.00	1.00	1.00	1.00	1.00
			(0.00)	(0.02)	(0.02)	(0.00)	(0.03)	(0.03)	(0.00)	(0.02)	(0.02)	(0.00)	(0.03)	(0.03)
2a	(0,0,1)	r	1.00	1.01	1.00	1.00	1.01	1.00	1.00	1.00	1.00	1.00	1.00	1.00
			(0.00)	(0.02)	(0.02)	(0.00)	(0.03)	(0.03)	(0.00)	(0.02)	(0.02)	(0.00)	(0.03)	(0.03)
2a	(0,0,1)	d	1.00	1.01	1.00	1.00	1.00	1.00	1.01	1.01	1.00	1.00	1.00	1.00
			(0.00)	(0.02)	(0.02)	(0.00)	(0.03)	(0.03)	(0.01)	(0.02)	(0.02)	(0.00)	(0.03)	(0.03)
2b	(0,0,0)	a	1.00	1.00	1.00	1.00	1.00	1.00	1.00	1.00	1.00	1.00	1.00	1.00
			(0.00)	(0.02)	(0.02)	(0.00)	(0.03)	(0.03)	(0.00)	(0.02)	(0.02)	(0.00)	(0.03)	(0.03)
2b	(1,0,0)	a	1.00	1.02	0.99	1.00	1.03	0.97	1.00	1.00	1.00	1.00	1.00	1.00
			(0.02)	(0.03)	(0.02)	(0.00)	(0.04)	(0.04)	(0.01)	(0.02)	(0.02)	(0.00)	(0.03)	(0.03)
2b	(1,0,0)	r	1.00	1.00	1.00	1.00	1.01	0.99	1.00	1.00	1.00	1.00	1.00	1.00
			(0.00)	(0.02)	(0.02)	(0.00)	(0.03)	(0.03)	(0.00)	(0.02)	(0.02)	(0.00)	(0.03)	(0.03)
2b	(1,0,0)	d	1.00	1.02	0.99	1.00	1.03	0.98	1.00	1.00	1.00	1.00	1.01	1.00
			(0.01)	(0.03)	(0.02)	(0.00)	(0.03)	(0.03)	(0.00)	(0.02)	(0.02)	(0.00)	(0.03)	(0.03)
2b	(0,1,0)	a	1.00	1.00	1.00	1.00	1.00	1.01	1.00	1.00	1.00	1.00	1.00	1.00
			(0.01)	(0.02)	(0.02)	(0.00)	(0.03)	(0.03)	(0.00)	(0.02)	(0.02)	(0.00)	(0.03)	(0.03)
2b	(0,0,1)	a	1.00	1.01	1.00	1.00	1.01	0.99	1.00	1.00	1.00	1.00	1.00	1.00
			(0.01)	(0.02)	(0.02)	(0.00)	(0.03)	(0.03)	(0.00)	(0.02)	(0.02)	(0.00)	(0.03)	(0.03)
2b	(0,0,1)	r	1.00	1.00	1.00	1.00	1.01	0.99	1.00	1.00	1.00	1.00	1.00	1.00
			(0.00)	(0.02)	(0.02)	(0.00)	(0.03)	(0.03)	(0.00)	(0.02)	(0.02)	(0.00)	(0.03)	(0.03)
2b	(0,0,1)	d	1.00	1.01	1.00	1.00	1.01	1.00	1.00	1.00	1.00	1.00	1.00	1.00
			(0.01)	(0.02)	(0.02)	(0.00)	(0.03)	(0.03)	(0.00)	(0.02)	(0.02)	(0.00)	(0.03)	(0.03)
2c	(1,0,0)	r	0.89	0.90	0.99	1.00	1.01	0.99	0.85	0.86	1.00	1.00	1.00	1.00
			(0.02)	(0.02)	(0.02)	(0.00)	(0.03)	(0.03)	(0.00)	(0.02)	(0.02)	(0.00)	(0.03)	(0.03)
2c	(1,0,0)	d	0.97	0.98	0.99	1.00	1.01	0.99	0.91	0.91	1.00	1.00	1.00	1.00
			(0.02)	(0.02)	(0.02)	(0.00)	(0.03)	(0.03)	(0.00)	(0.02)	(0.02)	(0.00)	(0.03)	(0.03)
2c	(0,0,1)	r	0.86	0.86	1.00	1.00	1.00	1.00	0.85	0.85	1.00	1.00	1.00	1.00
			(0.01)	(0.02)	(0.02)	(0.00)	(0.03)	(0.03)	(0.00)	(0.02)	(0.02)	(0.00)	(0.03)	(0.03)
2c	(0,0,1)	d	0.87	0.88	0.99	1.00	1.01	1.00	0.88	0.89	0.99	1.00	1.01	1.00
			(0.02)	(0.03)	(0.02)	(0.00)	(0.03)	(0.03)	(0.00)	(0.02)	(0.03)	(0.00)	(0.03)	(0.03)

Mean and standard deviation (SD) for l_{approx}, l_{mean} and the ratio l_{approx}/l_{mean} are shown over 200 simulation runs for gene-environment (GxE) interaction and marginal association analyses for two sample sizes, $n = 1000$ and 10000. M, genotype model (a: additive, r: recessive, d: dominant). b_G, b_{GE} and b_Z are effect size parameters of genotype, gene-environment interaction and covariate, respectively; 0 and 1 denote absence and presence of effect.

S3 Table (continued). Comparison between l_{approx} and l_{mean} in binary phenotype simulations

Scenario			GxE ($n = 1000$)			Marginal ($n = 1000$)			GxE ($n = 10000$)			Marginal ($n = 10000$)		
	(b_G, b_{GE}, b_Z)	M	l_{approx}	l_{mean}	$\frac{l_{approx}}{l_{mean}}$	l_{approx}	l_{mean}	$\frac{l_{approx}}{l_{mean}}$	l_{approx}	l_{mean}	$\frac{l_{approx}}{l_{mean}}$	l_{approx}	l_{mean}	$\frac{l_{approx}}{l_{mean}}$
2d	(1,0,0)	r	1.02	1.01	1.00	1.00	1.00	1.00	1.02	1.02	1.00	1.00	1.00	1.00
			(0.32)	(0.23)	(0.06)	(0.00)	(0.03)	(0.03)	(0.16)	(0.12)	(0.04)	(0.00)	(0.03)	(0.03)
2d	(1,0,0)	d	1.03	1.03	0.99	1.00	1.01	0.99	1.03	1.02	1.01	1.00	1.00	1.00
			(0.29)	(0.21)	(0.06)	(0.00)	(0.03)	(0.03)	(0.18)	(0.14)	(0.04)	(0.00)	(0.03)	(0.03)
2d	(0,0,1)	r	1.74	1.52	1.09	1.00	1.01	1.00	2.00	1.98	1.01	1.00	1.00	1.00
			(1.09)	(0.76)	(0.12)	(0.00)	(0.03)	(0.03)	(0.97)	(0.95)	(0.03)	(0.00)	(0.03)	(0.03)
2d	(0,0,1)	d	9.68	5.68	1.35	0.99	0.99	1.00	1.97	1.93	1.02	1.00	1.00	1.00
			(9.66)	(5.14)	(0.41)	(0.01)	(0.03)	(0.03)	(0.95)	(0.92)	(0.03)	(0.00)	(0.03)	(0.03)
2e	(0,0,0)	a	1.05	1.05	1.01	1.00	1.00	1.01	1.05	1.04	1.00	1.00	1.00	1.00
			(0.02)	(0.03)	(0.02)	(0.00)	(0.03)	(0.03)	(0.04)	(0.04)	(0.02)	(0.00)	(0.03)	(0.03)
2e	(1,0,0)	a	1.06	1.07	0.99	1.00	1.03	0.97	1.04	1.03	1.00	1.00	1.00	1.00
			(0.06)	(0.06)	(0.02)	(0.00)	(0.03)	(0.03)	(0.05)	(0.05)	(0.02)	(0.00)	(0.03)	(0.03)
2e	(1,0,0)	r	1.06	1.06	1.00	1.00	1.01	0.99	1.04	1.03	1.00	1.00	1.00	1.00
			(0.02)	(0.03)	(0.02)	(0.00)	(0.03)	(0.03)	(0.03)	(0.04)	(0.02)	(0.00)	(0.03)	(0.03)
2e	(1,0,0)	d	1.06	1.06	0.99	1.00	1.02	0.98	1.05	1.04	1.00	1.00	1.00	1.00
			(0.03)	(0.03)	(0.02)	(0.00)	(0.03)	(0.03)	(0.07)	(0.07)	(0.02)	(0.00)	(0.04)	(0.04)
2e	(0,1,0)	a	1.33	1.42	0.87	1.00	1.00	1.00	11.60	8.88	1.29	0.98	0.98	1.00
			(1.91)	(1.04)	(0.10)	(0.00)	(0.03)	(0.03)	(3.00)	(1.38)	(0.15)	(0.00)	(0.03)	(0.03)
2e	(0,0,1)	a	1.06	1.06	1.00	1.00	1.00	1.00	4.20	4.14	0.97	1.00	1.00	1.00
			(0.02)	(0.03)	(0.02)	(0.00)	(0.03)	(0.03)	(2.32)	(1.92)	(0.10)	(0.00)	(0.03)	(0.03)
2e	(0,0,1)	r	1.06	1.07	0.99	1.00	1.00	1.00	1.04	1.04	1.00	1.00	1.00	1.00
			(0.04)	(0.04)	(0.03)	(0.00)	(0.03)	(0.03)	(0.04)	(0.05)	(0.02)	(0.00)	(0.03)	(0.03)
2e	(0,0,1)	d	1.25	1.32	0.90	1.00	1.00	1.00	9.76	8.06	1.19	0.99	0.99	1.00
			(1.41)	(0.78)	(0.08)	(0.00)	(0.03)	(0.03)	(2.62)	(1.62)	(0.11)	(0.00)	(0.03)	(0.03)
3a	(0,0,0)	a	1.01	1.01	1.00	1.01	1.01	1.00	1.00	1.00	1.00	1.00	1.00	1.00
			(0.00)	(0.02)	(0.02)	(0.00)	(0.03)	(0.03)	(0.00)	(0.02)	(0.02)	(0.00)	(0.03)	(0.03)
3a	(1,0,0)	a	1.01	1.02	0.99	1.01	1.04	0.97	1.00	1.00	1.00	1.00	1.00	1.00
			(0.02)	(0.03)	(0.02)	(0.00)	(0.03)	(0.03)	(0.01)	(0.02)	(0.02)	(0.00)	(0.03)	(0.03)
3a	(1,0,0)	r	1.01	1.01	1.00	1.01	1.02	0.99	1.00	1.01	0.99	1.00	1.02	0.98
			(0.01)	(0.03)	(0.03)	(0.00)	(0.03)	(0.03)	(0.00)	(0.02)	(0.02)	(0.00)	(0.03)	(0.03)
3a	(1,0,0)	d	1.01	1.02	0.99	1.01	1.02	0.99	1.00	1.00	1.00	1.00	1.00	1.01
			(0.01)	(0.02)	(0.02)	(0.00)	(0.03)	(0.03)	(0.00)	(0.02)	(0.02)	(0.00)	(0.03)	(0.03)
3a	(0,1,0)	a	1.01	1.01	1.00	1.01	1.01	1.00	1.00	1.00	1.00	1.00	1.00	1.00
			(0.01)	(0.02)	(0.02)	(0.00)	(0.03)	(0.03)	(0.00)	(0.02)	(0.02)	(0.00)	(0.03)	(0.03)
3a	(0,0,1)	a	1.03	1.04	0.99	1.01	1.01	1.00	1.01	1.01	1.00	1.00	1.00	1.00
			(0.02)	(0.03)	(0.02)	(0.00)	(0.03)	(0.03)	(0.00)	(0.02)	(0.02)	(0.00)	(0.03)	(0.03)
3a	(0,0,1)	r	1.01	1.01	1.00	1.01	1.01	1.00	1.00	1.00	1.00	1.00	1.00	1.00
			(0.01)	(0.02)	(0.02)	(0.00)	(0.03)	(0.03)	(0.00)	(0.02)	(0.02)	(0.00)	(0.03)	(0.03)
3a	(0,0,1)	d	1.01	1.02	0.99	1.01	1.01	1.00	1.00	1.00	1.00	1.00	1.00	1.00
			(0.01)	(0.03)	(0.03)	(0.00)	(0.03)	(0.03)	(0.00)	(0.02)	(0.02)	(0.00)	(0.03)	(0.03)

Mean and standard deviation (SD) for l_{approx}, l_{mean} and the ratio l_{approx}/l_{mean} are shown over 200 simulation runs for gene-environment (GxE) interaction and marginal association analyses for two sample sizes, $n = 1000$ and 10000. M, genotype model (a: additive, r: recessive, d: dominant). b_G, b_{GE} and b_Z are effect size parameters of genotype, gene-environment interaction and covariate, respectively; 0 and 1 denote absence and presence of effect.

S3 Table (continued). Comparison between l_{approx} and l_{mean} in binary phenotype simulations

Scenario			GxE ($n = 1000$)			Marginal ($n = 1000$)			GxE ($n = 10000$)			Marginal ($n = 10000$)		
	(b_G, b_{GE}, b_Z)	M	l_{approx}	l_{mean}	$\frac{l_{approx}}{l_{mean}}$	l_{approx}	l_{mean}	$\frac{l_{approx}}{l_{mean}}$	l_{approx}	l_{mean}	$\frac{l_{approx}}{l_{mean}}$	l_{approx}	l_{mean}	$\frac{l_{approx}}{l_{mean}}$
3b	(0,0,0)	a	1.00 (0.00)	1.00 (0.02)	1.00 (0.02)	1.00 (0.00)	1.01 (0.03)	1.00 (0.03)	1.00 (0.00)	1.00 (0.02)	1.00 (0.02)	1.00 (0.00)	1.00 (0.03)	1.00 (0.03)
3b	(1,0,0)	a	1.00 (0.01)	1.02 (0.03)	0.98 (0.02)	1.00 (0.00)	1.03 (0.04)	0.97 (0.03)	1.00 (0.00)	1.00 (0.02)	1.00 (0.02)	1.00 (0.00)	1.00 (0.03)	1.00 (0.03)
3b	(1,0,0)	r	1.00 (0.00)	1.01 (0.02)	1.00 (0.02)	1.00 (0.00)	1.01 (0.03)	0.99 (0.03)	1.00 (0.00)	1.00 (0.02)	1.00 (0.02)	1.00 (0.00)	1.00 (0.03)	1.00 (0.03)
3b	(1,0,0)	d	1.00 (0.01)	1.01 (0.02)	0.99 (0.02)	1.00 (0.00)	1.02 (0.03)	0.98 (0.03)	1.00 (0.00)	1.00 (0.02)	1.00 (0.02)	1.00 (0.00)	1.00 (0.03)	1.00 (0.03)
3b	(0,1,0)	a	1.00 (0.04)	1.00 (0.05)	1.00 (0.02)	1.00 (0.01)	1.01 (0.03)	1.00 (0.03)	1.00 (0.01)	1.00 (0.02)	1.00 (0.02)	1.00 (0.00)	1.00 (0.03)	1.00 (0.03)
3b	(0,0,1)	a	1.03 (0.01)	1.02 (0.02)	1.00 (0.02)	1.00 (0.00)	1.00 (0.03)	1.01 (0.03)	1.00 (0.00)	1.00 (0.02)	1.00 (0.02)	1.00 (0.00)	1.00 (0.03)	1.00 (0.03)
3b	(0,0,1)	r	1.00 (0.00)	1.00 (0.02)	1.00 (0.02)	1.00 (0.00)	1.00 (0.03)	1.00 (0.03)	1.00 (0.00)	1.01 (0.02)	0.99 (0.02)	1.00 (0.00)	1.02 (0.03)	0.98 (0.03)
3b	(0,0,1)	d	1.01 (0.01)	1.01 (0.02)	1.00 (0.02)	1.00 (0.00)	1.00 (0.04)	1.00 (0.04)	1.01 (0.00)	1.01 (0.02)	1.00 (0.02)	1.00 (0.00)	1.00 (0.03)	1.00 (0.03)
3c	(0,0,0)	a	1.00 (0.00)	1.00 (0.02)	1.00 (0.02)	1.00 (0.00)	1.00 (0.03)	1.00 (0.03)	1.00 (0.00)	1.00 (0.02)	1.00 (0.02)	1.00 (0.00)	1.00 (0.03)	1.00 (0.03)
3c	(1,0,0)	a	1.00 (0.00)	1.01 (0.02)	0.99 (0.02)	1.00 (0.00)	1.01 (0.03)	0.99 (0.03)	1.00 (0.00)	1.00 (0.02)	1.00 (0.02)	1.00 (0.00)	1.00 (0.03)	1.00 (0.03)
3c	(1,0,0)	r	1.00 (0.00)	1.01 (0.02)	0.99 (0.02)	1.00 (0.00)	1.02 (0.03)	0.99 (0.03)	1.00 (0.00)	1.00 (0.02)	1.00 (0.02)	1.00 (0.00)	1.00 (0.03)	1.00 (0.03)
3c	(1,0,0)	d	1.00 (0.00)	1.02 (0.02)	0.99 (0.02)	1.00 (0.00)	1.02 (0.03)	0.98 (0.03)	1.00 (0.00)	1.00 (0.02)	1.00 (0.02)	1.00 (0.00)	1.00 (0.03)	1.00 (0.03)
3c	(0,1,0)	a	1.00 (0.00)	1.00 (0.02)	1.00 (0.03)	1.00 (0.00)	1.00 (0.04)	1.00 (0.03)	1.00 (0.00)	1.00 (0.02)	1.00 (0.02)	1.00 (0.00)	1.00 (0.03)	1.00 (0.03)
3c	(0,0,1)	a	1.00 (0.00)	1.01 (0.02)	0.99 (0.02)	1.00 (0.00)	1.01 (0.03)	1.00 (0.03)	1.00 (0.00)	1.00 (0.02)	1.00 (0.02)	1.00 (0.00)	1.00 (0.03)	1.00 (0.03)
3c	(0,0,1)	r	1.00 (0.00)	1.01 (0.02)	1.00 (0.02)	1.00 (0.00)	1.01 (0.03)	1.00 (0.03)	1.00 (0.00)	1.01 (0.02)	0.99 (0.02)	1.00 (0.00)	1.03 (0.03)	0.98 (0.03)
3c	(0,0,1)	d	1.00 (0.00)	1.01 (0.02)	1.00 (0.02)	1.00 (0.00)	1.01 (0.03)	1.00 (0.03)	1.00 (0.00)	1.00 (0.02)	1.00 (0.02)	1.00 (0.00)	1.03 (0.04)	0.97 (0.04)
3d	(0,0,0)	a	1.00 (0.00)	1.01 (0.02)	1.00 (0.02)	1.00 (0.00)	1.01 (0.03)	0.99 (0.03)	1.00 (0.00)	1.00 (0.02)	1.00 (0.02)	1.00 (0.00)	1.00 (0.03)	1.00 (0.03)
3d	(1,0,0)	a	1.00 (0.00)	1.01 (0.02)	0.99 (0.02)	1.00 (0.00)	1.02 (0.03)	0.98 (0.03)	1.00 (0.00)	1.00 (0.02)	1.00 (0.02)	1.00 (0.00)	1.00 (0.03)	1.00 (0.03)
3d	(1,0,0)	r	1.00 (0.00)	1.01 (0.02)	1.00 (0.02)	1.00 (0.00)	1.01 (0.03)	1.00 (0.03)	1.00 (0.00)	1.01 (0.02)	0.99 (0.02)	1.00 (0.00)	1.02 (0.03)	0.98 (0.03)
3d	(1,0,0)	d	1.00 (0.01)	1.02 (0.03)	0.99 (0.02)	1.00 (0.00)	1.02 (0.03)	0.98 (0.03)	1.00 (0.00)	1.00 (0.02)	1.00 (0.02)	1.00 (0.00)	1.00 (0.03)	1.00 (0.03)
3d	(0,1,0)	a	1.00 (0.03)	1.00 (0.04)	1.00 (0.02)	1.00 (0.00)	1.00 (0.03)	1.00 (0.03)	1.00 (0.01)	1.00 (0.02)	1.00 (0.02)	1.00 (0.00)	1.00 (0.03)	1.00 (0.03)
3d	(0,0,1)	a	1.01 (0.01)	1.01 (0.03)	1.00 (0.02)	1.00 (0.00)	1.03 (0.04)	0.97 (0.03)	1.03 (0.01)	1.03 (0.02)	1.00 (0.02)	1.00 (0.00)	0.99 (0.03)	1.00 (0.03)
3d	(0,0,1)	r	1.00 (0.00)	1.00 (0.02)	1.00 (0.02)	1.00 (0.00)	1.00 (0.03)	1.00 (0.03)	1.00 (0.00)	1.00 (0.02)	1.00 (0.02)	1.00 (0.00)	1.00 (0.03)	1.00 (0.03)
3d	(0,0,1)	d	1.01 (0.01)	1.02 (0.02)	0.99 (0.02)	1.00 (0.00)	1.03 (0.03)	0.97 (0.03)	1.02 (0.01)	1.02 (0.02)	1.00 (0.02)	1.00 (0.00)	1.00 (0.03)	1.00 (0.03)

Mean and standard deviation (SD) for l_{approx}, l_{mean} and the ratio l_{approx}/l_{mean} are shown over 200 simulation runs for gene-environment (GxE) interaction and marginal association analyses for two sample sizes, $n = 1000$ and 10000. M, genotype model (a: additive, r: recessive, d: dominant). b_G, b_{GE} and b_Z are effect size parameters of genotype, gene-environment interaction and covariate, respectively; 0 and 1 denote absence and presence of effect.

S3 Table (continued). Comparison between l_{approx} and l_{mean} in binary phenotype simulations

Scenario			GxE ($n = 1000$)			Marginal ($n = 1000$)			GxE ($n = 10000$)			Marginal ($n = 10000$)		
	(b_G, b_{GE}, b_Z)	M	l_{approx}	l_{mean}	$\frac{l_{approx}}{l_{mean}}$	l_{approx}	l_{mean}	$\frac{l_{approx}}{l_{mean}}$	l_{approx}	l_{mean}	$\frac{l_{approx}}{l_{mean}}$	l_{approx}	l_{mean}	$\frac{l_{approx}}{l_{mean}}$
4a	(0,0,0)	a	1.00	1.00	1.01	1.00	1.00	1.01	1.00	1.01	1.00	1.00	1.01	1.00
			(0.00)	(0.07)	(0.07)	(0.00)	(0.10)	(0.09)	(0.00)	(0.07)	(0.07)	(0.00)	(0.09)	(0.09)
4a	(1,0,0)	a	1.00	1.19	0.84	1.00	1.39	0.73	1.00	2.25	0.45	1.00	3.58	0.28
			(0.02)	(0.09)	(0.06)	(0.00)	(0.17)	(0.09)	(0.01)	(0.22)	(0.05)	(0.00)	(0.42)	(0.04)
4a	(1,0,0)	r	1.00	1.00	1.01	1.00	1.01	1.00	1.00	1.01	1.00	1.00	1.01	1.00
			(0.00)	(0.06)	(0.06)	(0.00)	(0.09)	(0.08)	(0.00)	(0.06)	(0.06)	(0.00)	(0.09)	(0.09)
4a	(1,0,0)	d	1.00	1.05	0.96	1.00	1.10	0.92	1.00	1.59	0.63	1.00	2.17	0.47
			(0.01)	(0.07)	(0.07)	(0.00)	(0.12)	(0.10)	(0.00)	(0.14)	(0.06)	(0.00)	(0.27)	(0.06)
4a	(0,1,0)	a	1.00	1.00	1.01	1.00	1.00	1.01	1.00	1.01	1.00	1.00	1.01	1.00
			(0.02)	(0.07)	(0.07)	(0.00)	(0.09)	(0.09)	(0.01)	(0.07)	(0.07)	(0.00)	(0.09)	(0.09)
4a	(0,0,1)	a	1.01	1.12	0.91	1.00	1.00	1.01	1.02	2.28	0.45	1.00	0.99	1.01
			(0.01)	(0.09)	(0.07)	(0.00)	(0.09)	(0.09)	(0.01)	(0.16)	(0.03)	(0.00)	(0.09)	(0.09)
4a	(0,0,1)	r	1.00	1.02	0.99	1.00	1.01	1.00	1.00	1.17	0.86	1.00	1.00	1.01
			(0.00)	(0.07)	(0.06)	(0.00)	(0.10)	(0.09)	(0.00)	(0.10)	(0.07)	(0.00)	(0.08)	(0.08)
4a	(0,0,1)	d	1.02	1.09	0.94	1.00	1.01	1.00	1.01	1.85	0.55	1.00	1.00	1.01
			(0.02)	(0.09)	(0.07)	(0.00)	(0.09)	(0.09)	(0.01)	(0.16)	(0.05)	(0.00)	(0.09)	(0.09)
4b	(0,0,0)	a	1.00	1.00	1.00	1.00	1.01	1.00	1.00	1.00	1.00	1.00	1.00	1.00
			(0.00)	(0.02)	(0.02)	(0.00)	(0.04)	(0.04)	(0.00)	(0.02)	(0.02)	(0.00)	(0.03)	(0.03)
4b	(1,0,0)	a	1.00	1.01	0.99	1.00	1.02	0.99	1.00	1.00	1.00	1.00	1.00	1.00
			(0.00)	(0.02)	(0.02)	(0.00)	(0.03)	(0.03)	(0.00)	(0.02)	(0.02)	(0.00)	(0.03)	(0.03)
4b	(1,0,0)	r	1.00	1.01	1.00	1.00	1.00	1.00	1.00	1.01	0.99	1.00	1.02	0.98
			(0.00)	(0.02)	(0.02)	(0.00)	(0.03)	(0.03)	(0.00)	(0.02)	(0.02)	(0.00)	(0.03)	(0.03)
4b	(1,0,0)	d	1.00	1.01	0.99	1.00	1.02	0.98	1.00	1.00	1.00	1.00	1.00	1.00
			(0.01)	(0.02)	(0.02)	(0.00)	(0.03)	(0.03)	(0.00)	(0.02)	(0.02)	(0.00)	(0.03)	(0.03)
4b	(0,1,0)	a	1.00	1.00	1.00	1.00	1.00	1.00	1.00	1.00	1.00	1.00	1.00	1.00
			(0.02)	(0.03)	(0.02)	(0.00)	(0.03)	(0.03)	(0.00)	(0.02)	(0.02)	(0.00)	(0.03)	(0.03)
4b	(0,0,1)	a	1.02	1.03	0.99	1.00	1.00	1.00	1.02	1.02	1.00	1.00	1.00	1.00
			(0.02)	(0.03)	(0.02)	(0.00)	(0.03)	(0.03)	(0.01)	(0.02)	(0.02)	(0.00)	(0.03)	(0.03)
4b	(0,0,1)	r	1.00	1.00	1.00	1.00	1.00	1.00	1.00	1.00	1.00	1.00	1.00	1.00
			(0.00)	(0.02)	(0.02)	(0.00)	(0.03)	(0.03)	(0.00)	(0.02)	(0.02)	(0.00)	(0.03)	(0.03)
4b	(0,0,1)	d	1.01	1.02	0.99	1.00	1.00	1.00	1.00	1.02	0.98	1.00	1.00	1.00
			(0.01)	(0.02)	(0.02)	(0.00)	(0.03)	(0.03)	(0.00)	(0.02)	(0.02)	(0.00)	(0.03)	(0.03)
4c	(0,0,0)	a	1.00	1.00	1.00	1.00	1.00	1.01	1.00	1.00	1.00	1.00	1.00	1.00
			(0.00)	(0.05)	(0.05)	(0.00)	(0.07)	(0.07)	(0.00)	(0.06)	(0.06)	(0.00)	(0.09)	(0.08)
4c	(1,0,0)	a	1.00	1.11	0.91	1.00	1.23	0.83	1.00	1.07	0.94	1.00	1.14	0.89
			(0.01)	(0.08)	(0.06)	(0.00)	(0.13)	(0.09)	(0.00)	(0.06)	(0.06)	(0.00)	(0.11)	(0.09)
4c	(1,0,0)	r	1.00	1.01	1.00	1.00	1.01	1.00	1.00	1.27	0.79	1.00	1.55	0.65
			(0.00)	(0.05)	(0.05)	(0.00)	(0.07)	(0.07)	(0.00)	(0.09)	(0.06)	(0.00)	(0.17)	(0.07)
4c	(1,0,0)	d	1.00	1.09	0.92	1.00	1.18	0.86	1.00	1.25	0.80	1.00	1.50	0.68
			(0.01)	(0.06)	(0.06)	(0.00)	(0.11)	(0.08)	(0.00)	(0.10)	(0.06)	(0.00)	(0.17)	(0.07)
4c	(0,1,0)	a	1.00	1.00	1.01	1.00	1.00	1.01	1.00	1.00	1.00	1.00	1.01	1.00
			(0.02)	(0.05)	(0.05)	(0.00)	(0.08)	(0.08)	(0.01)	(0.05)	(0.05)	(0.00)	(0.09)	(0.08)
4c	(0,0,1)	a	1.03	1.14	0.91	1.00	0.99	1.02	1.00	1.06	0.95	1.00	1.00	1.01
			(0.02)	(0.08)	(0.06)	(0.00)	(0.07)	(0.07)	(0.00)	(0.06)	(0.05)	(0.00)	(0.08)	(0.08)
4c	(0,0,1)	r	1.00	1.01	1.00	1.00	1.00	1.01	1.00	1.02	0.98	1.00	1.00	1.01
			(0.00)	(0.05)	(0.05)	(0.00)	(0.08)	(0.08)	(0.00)	(0.06)	(0.06)	(0.00)	(0.08)	(0.08)
4c	(0,0,1)	d	1.01	1.06	0.95	1.00	1.01	0.99	1.01	1.69	0.60	1.00	1.00	1.00
			(0.01)	(0.06)	(0.06)	(0.00)	(0.08)	(0.08)	(0.00)	(0.14)	(0.05)	(0.00)	(0.08)	(0.08)

Mean and standard deviation (SD) for l_{approx}, l_{mean} and the ratio l_{approx}/l_{mean} are shown over 200 simulation runs for gene-environment (GxE) interaction and marginal association analyses for two sample sizes, $n = 1000$ and 10000. M, genotype model (a: additive, r: recessive, d: dominant). b_G, b_{GE} and b_Z are effect size parameters of genotype, gene-environment interaction and covariate, respectively; 0 and 1 denote absence and presence of effect.
