# Supplementary material for: Quick assessment for systematic test statistic inflation/deflation due to null model misspecifications in genome-wide environment interaction studies
Source: PLoS One. 2019 Jul 18;14(7):e0219825. doi: 10.1371/journal.pone.0219825 (PMC6638962; doi:10.1371/journal.pone.0219825)
Supplement: S1 Appendix — Details of theoretical results and simulation studies. (PDF) [file pone.0219825.s011.pdf]

S1 Appendix: Technical details

Derivation of approximation formula (7) in the main text

Preliminary

Here we derive the approximation formula (7) in the main text. We have n samples with phenotypic value (binary, numeric value, or a factor) denoted by $y_1, \ldots, y_n$, and L genetic variants, $\mathbf{g}_l = (g_{l,1}, \ldots, g_{l,n})^T$ for $l = 1, \ldots, L$, which are to be tested for association with the phenotype. The tested variables at the lth locus are generically denoted as $\mathbf{w}_{l,i}^T = (w_{l,i1}, \ldots, w_{l,ip})$, with p variables including the effect of $\mathbf{g}_l$ itself or an interaction between $\mathbf{g}_l$ and an environment variable. We also have q covariates (e.g. sex or age) $\mathbf{z}_i^T = (z_{i1}, \ldots, z_{iq})$ to be adjusted in common for all L tests. We consider L hypothesis tests of the null hypothesis $H_{0l} : \beta_l = \mathbf{0}$ under the following regression model for the conditional mean of y_i with transformation,

$$\eta_i = \eta\{E(y_i|\mathbf{w}_{l,i}^T, \mathbf{z}_i^T)\} = \mathbf{w}_{l,i}^T\beta_l + \mathbf{z}_i^T\gamma_l, \tag{S1}$$

for $i = 1, \ldots, n$, where η is a monotone increasing function, and $\beta_l^T = (\beta_{l,1}, \ldots, \beta_{l,p})$ and $\gamma_l^T = (\gamma_{l,1}, \ldots, \gamma_{l,q})$ are the regression coefficients. The above model reduces to the ordinary linear regression model if η is the identity function and y_i follows a Gaussian distribution. The model reduces to the logistic regression model if η is the logit function and y_i follows a Bernoulli distribution.

We consider the lth genetic variant $\mathbf{g}_l$ separately for $l = 1, \ldots, L$, where n is the sample size. Let $E_{\mathbf{g}_l}$ denote the expectation with respect to the marginal distribution of $\mathbf{g}_l$. The assumption is that, for a given l, genotypes $g_{l,1}, \ldots, g_{l,n}$ identically and independently follow a distribution whose all moments are finite, where the jth moment is denoted by $\mu_{l,j} = E_{\mathbf{g}_l}(g_{l,i}^j)$.

As shown in section "Influence of centering $g_{l,i}$ and coding of $\mathbf{x}_i$" of this S1 Appendix, substracting any constant from $g_{l,i}$ does not change the score test for testing $\beta_l = 0$. Thus, without loss of generality, we can assume that $\mu_{l,1} = E_{\mathbf{g}_l}(g_{l,i}) = 0$ by subtracting the mean. We also denote the variance by $\mu_{l,2} = \sigma_l^2$. Let $\mathbf{u} = (u_i)$, $\mathbf{W}_l = (w_{l,ia})$ with $w_{l,ia} = g_{l,i}x_{ia}$ $(a = 1, \ldots, p)$, and $\mathbf{Z} = (z_{ic})$ $(c = 1, \ldots, q)$, in which $i = 1, \ldots, n$, where $\mathbf{u}$ depends on phenotype $y_1, \ldots, y_n$, x_{ia} is the ath environment variable for ith subject, and z_{ic} is the cth covariate for ith subject. We denote $\widetilde{\mathbf{W}}_l = \mathbf{\Omega}^{1/2}\mathbf{W}_l$, $\widetilde{\mathbf{Z}} = \mathbf{\Omega}^{1/2}\mathbf{Z}$, $\widetilde{\mathbf{X}} = \mathbf{\Omega}^{1/2}\mathbf{X}$, $\mathbf{\Omega} = diag(\omega_1, \ldots, \omega_n)$, the ω_is are positive values specific to the regression model. Then, $\widetilde{w}_{l,ia} = g_{l,i}\widetilde{x}_{ia}$ $(a = 1, \ldots, p)$. Let $\mathbf{Q}_{\widetilde{\mathbf{Z}}} = \mathbf{I} - \mathbf{P}_{\widetilde{\mathbf{Z}}}$, where $\mathbf{P}_{\widetilde{\mathbf{Z}}} = \widetilde{\mathbf{Z}}(\widetilde{\mathbf{Z}}^T\widetilde{\mathbf{Z}})^{-1}\widetilde{\mathbf{Z}}^T$ is the projection onto $\widetilde{\mathbf{Z}}$. For the following arguments, we make assumptions that $\max_{i,a} |\widetilde{x}_{ia}| < \infty$ and $\max_i |u_i| < \infty$ as $n \to \infty$. We denote the equality by ignoring $O(n^{-1})$ terms by '$\approx$'.

Let A_l^{ab} and $B_{l,ab}$ represent the (a, b)-element of matrixes $\mathbf{A}_l^{-1}$ and $\mathbf{B}_l$, where

$$\mathbf{A}_l = \widetilde{\mathbf{W}}_l^T\mathbf{Q}_{\widetilde{\mathbf{Z}}}\widetilde{\mathbf{W}}_l \quad \text{and} \quad \mathbf{B}_l = \widetilde{\mathbf{W}}_l^T\mathbf{r}\mathbf{r}^T\widetilde{\mathbf{W}}_l,$$

respectively, in which

$$\mathbf{r} = \mathbf{Q}_{\widetilde{\mathbf{Z}}}\mathbf{u}.$$

Now we study the test statistic (1) in the main text,

$$\begin{aligned}
t_l &= \mathbf{u}^T(\mathbf{Q}_{\widetilde{\mathbf{Z}}}\widetilde{\mathbf{W}}_l)(\widetilde{\mathbf{W}}_l^T\mathbf{Q}_{\widetilde{\mathbf{Z}}}\widetilde{\mathbf{W}}_l)^{-1}(\mathbf{Q}_{\widetilde{\mathbf{Z}}}\widetilde{\mathbf{W}}_l)^T\mathbf{u} \\
&= \mathrm{tr}\{(\widetilde{\mathbf{W}}_l^T\mathbf{Q}_{\widetilde{\mathbf{Z}}}\widetilde{\mathbf{W}}_l)^{-1}(\mathbf{Q}_{\widetilde{\mathbf{Z}}}\widetilde{\mathbf{W}}_l)^T\mathbf{u}\mathbf{u}^T(\mathbf{Q}_{\widetilde{\mathbf{Z}}}\widetilde{\mathbf{W}}_l)\} \\
&= \mathrm{tr}\{(\widetilde{\mathbf{W}}_l^T\mathbf{Q}_{\widetilde{\mathbf{Z}}}\widetilde{\mathbf{W}}_l)^{-1}(\widetilde{\mathbf{W}}_l^T\mathbf{r}\mathbf{r}^T\widetilde{\mathbf{W}}_l)\} \\
&= \mathrm{tr}(\mathbf{A}_l^{-1}\mathbf{B}_l) \\
&= \sum_{a=1}^{p}\sum_{b=1}^{p} A_l^{ab}B_{l,ab}.
\end{aligned}$$

Since we assumed that $g_{l,i}$ is centered such that $\mu_l = 0$,

$$E_{\mathbf{g}_l}(\mathbf{A}_l) = E_{\mathbf{g}_l}(\widetilde{\mathbf{W}}_l^T\mathbf{Q}_{\widetilde{\mathbf{Z}}}\widetilde{\mathbf{W}}_l) = E_{\mathbf{g}_l}\left\{\sum_{i=1}^{n}\sum_{j=1}^{n} g_{l,i}g_{l,j}\widetilde{\mathbf{x}}_i\widetilde{\mathbf{x}}_j^T(\mathbf{Q}_{\widetilde{\mathbf{Z}}})_{ij}\right\} = \sigma_l^2\sum_{i=1}^{n}\widetilde{\mathbf{x}}_i\widetilde{\mathbf{x}}_i^T(\mathbf{Q}_{\widetilde{\mathbf{Z}}})_{ii}$$

and

$$E_{\mathbf{g}_l}(\mathbf{B}_l) = E_{\mathbf{g}_l}\{(\mathbf{Q}_{\widetilde{\mathbf{Z}}}\widetilde{\mathbf{W}}_l)^T\mathbf{u}\mathbf{u}^T(\mathbf{Q}_{\widetilde{\mathbf{Z}}}\widetilde{\mathbf{W}}_l)\} = E_{\mathbf{g}_l}\left\{\sum_{i=1}^n\sum_{j=1}^n g_{l,i}g_{l,j}\widetilde{\mathbf{x}}_i\widetilde{\mathbf{x}}_j^T(\mathbf{Q}_{\widetilde{\mathbf{Z}}}\mathbf{u})_i(\mathbf{Q}_{\widetilde{\mathbf{Z}}}\mathbf{u})_j\right\}$$
$$= \sigma_l^2\sum_{i=1}^n\widetilde{\mathbf{x}}_i\widetilde{\mathbf{x}}_i^T(\mathbf{Q}_{\widetilde{\mathbf{Z}}}\mathbf{u})_i^2.$$

Therefore, if the approximation

$$E_{\mathbf{g}_l}(t_l) \approx \mathrm{tr}[\{E_{\mathbf{g}_l}(\mathbf{A}_l)\}^{-1}E_{\mathbf{g}_l}(\mathbf{B}_l)\}] \tag{S2}$$

holds, the approximation formula (7) in the main text is derived.

In what follows, we verify eq. (S2). To this end, Let $\bar{\mathbf{A}}_l = E_{\mathbf{g}_l}(\mathbf{A}_l)$ and $\bar{\mathbf{B}}_l = E_{\mathbf{g}_l}(\mathbf{B}_l)$. Then,

$$\begin{aligned}
E_{\mathbf{g}_l}(t_l) &= E_{\mathbf{g}_l}\{\mathrm{tr}(\mathbf{A}_l^{-1}\mathbf{B}_l)\}\\
&= E_{\mathbf{g}_l}(\mathrm{tr}[\{\bar{\mathbf{A}}_l - (\bar{\mathbf{A}}_l - \mathbf{A}_l)\}^{-1}\mathbf{B}_l])\\
&= E_{\mathbf{g}_l}(\mathrm{tr}[\bar{\mathbf{A}}_l^{-1/2}\{\mathbf{I} - \bar{\mathbf{A}}_l^{-1/2}(\bar{\mathbf{A}}_l - \mathbf{A}_l)\bar{\mathbf{A}}_l^{-1/2}\}^{-1}\bar{\mathbf{A}}_l^{-1/2}\mathbf{B}_l])\\
&= E_{\mathbf{g}_l}[\mathrm{tr}\{(\mathbf{I} - \mathbf{M}_l)^{-1}\mathbf{N}_l\}]\\
&= E_{\mathbf{g}_l}[\mathrm{tr}\{(\mathbf{I} + \sum_{m=1}^{\infty}\mathbf{M}_l^m)\mathbf{N}_l\}]\\
&= E_{\mathbf{g}_l}\{\mathrm{tr}(\mathbf{N}_l)\} + \sum_{m=1}^{\infty}E_{\mathbf{g}_l}\{\mathrm{tr}(\mathbf{M}_l^m\mathbf{N}_l)\} \qquad \text{(S3)}
\end{aligned}$$

where

$$\mathbf{M}_l = \mathbf{I} - \mathbf{L}_l, \quad \mathbf{L}_l = \bar{\mathbf{A}}_l^{-1/2}\mathbf{A}_l\bar{\mathbf{A}}_l^{-1/2} \quad \text{and} \quad \mathbf{N}_l = \bar{\mathbf{A}}_l^{-1/2}\mathbf{B}_l\bar{\mathbf{A}}_l^{-1/2}.$$

Also, define

$$\bar{\mathbf{L}}_l = E_{\mathbf{g}_l}(\mathbf{L}_l) = \bar{\mathbf{A}}_l^{-1/2}\bar{\mathbf{A}}_l\bar{\mathbf{A}}_l^{-1/2} \quad \text{and} \quad \bar{\mathbf{N}}_l = E_{\mathbf{g}_l}(\mathbf{N}_l) = \bar{\mathbf{A}}_l^{-1/2}\bar{\mathbf{B}}_l\bar{\mathbf{A}}_l^{-1/2}.$$

We express $A_{l,ab} = (\mathbf{A}_l)_{ab}$ and $B_{l,ab} = (\mathbf{B}_l)_{ab}$ in detail as follows.

$$
\begin{aligned}
A_{l,ab} &= (\widetilde{\mathbf{W}}_l^T \mathbf{Q}_{\widetilde{\mathbf{Z}}} \widetilde{\mathbf{W}}_l)_{ab} = \sum_{i=1}^n \sum_{j=1}^n \widetilde{w}_{l,ia} \widetilde{w}_{l,jb} (\mathbf{Q}_{\widetilde{\mathbf{Z}}})_{ij} \\
&= \sum_{i=1}^n \sum_{j=1}^n g_{l,i} g_{l,j} \widetilde{x}_{l,ia} \widetilde{x}_{l,jb} (\mathbf{Q}_{\widetilde{\mathbf{Z}}})_{ij} = \sum_{i=1}^n \sum_{j=1}^n g_{l,i} g_{l,j} G_{l,ab,ij}, \\
B_{l,ab} &= (\widetilde{\mathbf{W}}_l^T \mathbf{r}\mathbf{r}^T \widetilde{\mathbf{W}}_l)_{ab} = \sum_{i=1}^n \sum_{j=1}^n \widetilde{w}_{l,ia} \widetilde{w}_{l,jb} (\mathbf{r}\mathbf{r}^T)_{ij} \\
&= \sum_{i=1}^n \sum_{j=1}^n g_{l,i} g_{l,j} \widetilde{x}_{ia} \widetilde{x}_{jb} (\mathbf{r}\mathbf{r}^T)_{ij} = \sum_{i=1}^n \sum_{j=1}^n g_{l,i} g_{l,j} F_{l,ab,ij},
\end{aligned}
$$

in which

$$
G_{l,ab,ij} = \widetilde{x}_{l,ia} \widetilde{x}_{l,jb} (\mathbf{Q}_{\widetilde{\mathbf{Z}}})_{ij} \quad \text{and} \quad F_{l,ab,ij} = \widetilde{x}_{l,ia} \widetilde{x}_{l,jb} (\mathbf{r}\mathbf{r}^T)_{ij}.
$$

Because $g_{l,i}$s are identically and independently distributed with mean zero and variance σ_l^2, we have

$$
\bar{A}_{l,ab} = \sum_{i=1}^n \sum_{j=1}^n E_{\mathbf{g}_l}(g_{l,i} g_{l,j}) G_{l,ab,ij} = \sigma_l^2 \sum_{i=1}^n G_{l,ab,ii} = \sigma_l^2 \sum_{i=1}^n \widetilde{x}_{ia} \widetilde{x}_{ib} (\mathbf{Q}_{\widetilde{\mathbf{Z}}})_{ii}, \tag{S4}
$$

$$
\bar{B}_{l,ab} = \sum_{i=1}^n \sum_{j=1}^n E_{\mathbf{g}_l}(g_{l,i} g_{l,j}) F_{l,ab,ij} = \sigma_l^2 \sum_{i=1}^n F_{l,ab,ii} = \sigma_l^2 \sum_{i=1}^n \widetilde{x}_{ia} \widetilde{x}_{ib} (\mathbf{r}\mathbf{r}^T)_{ii}. \tag{S5}
$$

From the assumption that $\max_{i,a} |\widetilde{x}_{ia}| < \infty$,

$$
|\sum_{i=1}^n G_{l,ab,ii}| \leq \max_{i,a} |\widetilde{x}_{ia}|^2 \sum_{i=1}^n (\mathbf{Q}_{\widetilde{\mathbf{Z}}})_{ii} = \max_{i,a} |\widetilde{x}_{ia}|^2 (n-q) = O(n),
$$

which implies that $\bar{\mathbf{A}}_l = O(n)$, and hence, $\bar{\mathbf{A}}_l^{-1/2} = O(n^{-1/2})$. Similarly, by $\max_i |u_i| < \infty$,

$$
\begin{aligned}
|\sum_{i=1}^n F_{l,ab,ii}| &\leq \max_{i,a} |\widetilde{x}_{ia}|^2 ||\mathbf{r}||^2 = \max_{i,a} |\widetilde{x}_{ia}|^2 ||\mathbf{Q}_{\widetilde{\mathbf{Z}}} \mathbf{u}||^2 \\
&\leq \max_{i,a} |\widetilde{x}_{ia}|^2 ||\mathbf{u}||^2 \leq \max_{i,a} |\widetilde{x}_{ia}|^2 \max_i |u_i|^2 n = O(n),
\end{aligned}
$$

which implies that $\bar{\mathbf{B}}_l = O(n)$.

Define $\widetilde{x}_{ia}^* = \sum_{c=1}^p (\bar{\mathbf{A}}_l^{-1/2})_{ac} \widetilde{x}_{ic}$. By the assumption that $\max_{i,a} |\widetilde{x}_{ia}| < \infty$ as well

as that $\bar{\mathbf{A}}_l^{-1/2} = O(n^{-1/2})$, we have

$$\max_{i,a} |\widetilde{x}^*_{ia}| = O(n^{-1/2}). \tag{S6}$$

Then, let

$$G^*_{l,ab,ij} = \sum_{c=1}^{p}\sum_{d=1}^{p}(\bar{\mathbf{A}}_l^{-1/2})_{ac}(\bar{\mathbf{A}}_l^{-1/2})_{bd}G_{l,cd,ij} = \widetilde{x}^*_{ia}\widetilde{x}^*_{jb}(\mathbf{Q}_{\widetilde{\mathbf{Z}}})_{ij},$$
$$F^*_{l,ab,ij} = \sum_{c=1}^{p}\sum_{d=1}^{p}(\bar{\mathbf{A}}_l^{-1/2})_{ac}(\bar{\mathbf{A}}_l^{-1/2})_{bd}F_{l,cd,ij} = \widetilde{x}^*_{ia}\widetilde{x}^*_{jb}(\mathbf{r}\mathbf{r}^T)_{ij},$$

and then,

$$L_{l,ab} = (\bar{\mathbf{A}}_l^{-1/2}\mathbf{A}_l\bar{\mathbf{A}}_l^{-1/2})_{ab} = \sum_{i=1}^{n}\sum_{j=1}^{n} g_{l,i}g_{l,j}G^*_{l,ab,ij},$$
$$N_{l,ab} = (\bar{\mathbf{A}}_l^{-1/2}\mathbf{B}_l\bar{\mathbf{A}}_l^{-1/2})_{ab} = \sum_{i=1}^{n}\sum_{j=1}^{n} g_{l,i}g_{l,j}F^*_{l,ab,ij}.$$

Therefore, we have

$$\bar{L}_{l,ab} = \sigma_l^2\sum_{i=1}^{n} G^*_{l,ab,ii} \quad \text{and} \quad \bar{N}_{l,ab} = \sigma_l^2\sum_{i=1}^{n} F^*_{l,ab,ii}, \tag{S7}$$

both of which are of order $O(1)$ by the similar arguments above:

$$|\sum_{i=1}^{n} G^*_{l,ab,ii}| \le \max_{i,a}|\widetilde{x}^*_{ia}|^2 O(n) = O(1),$$

and

$$|\sum_{i=1}^{n} F^*_{l,ab,ii}| \le \max_{i,a}|\widetilde{x}^*_{ia}|^2 O(n) = O(1).$$

Derivation

Now recall eq. (S3),

$$E_{\mathbf{g}_l}(t_l) = E_{\mathbf{g}_l}\{\text{tr}(\mathbf{N}_l)\} + \sum_{m=1}^{\infty} E_{\mathbf{g}_l}\{\text{tr}(\mathbf{M}_l^m\mathbf{N}_l)\}.$$

We will show that the first term is the dominant term being of order $O(1)$, and, consequently,

$$E_{\mathbf{g}_l}(t_l) \approx E_{\mathbf{g}_l}\{\mathrm{tr}(\mathbf{N}_l)\},$$

which is of order $O(1)$.

The first term: We immediately have that

$$E_{\mathbf{g}_l}\{\mathrm{tr}(\mathbf{N}_l)\} = \mathrm{tr}(\bar{\mathbf{N}}_l) = \mathrm{tr}(\bar{\mathbf{A}}_l^{-1/2}\bar{\mathbf{B}}_l\bar{\mathbf{A}}_l^{-1/2}) = \mathrm{tr}(\bar{\mathbf{A}}_l^{-1}\bar{\mathbf{B}}_l),$$

the order of which is $O(1)$ as shown in (S7) below.

The second term: In what follows, we use induction to show that

$$E_{\mathbf{g}_l}\{\mathrm{tr}(\mathbf{M}_l^m\mathbf{N}_l)\} \approx 0$$

for any $m \geq 1$, which implies that the second term is negligible. As the induction step, first, we show that $E_{\mathbf{g}_l}\{\mathrm{tr}(\mathbf{M}_l^m\mathbf{N}_l)\} \approx 0$ for $m = 1$ and 2. Subsequently, assuming that $E_{\mathbf{g}_l}\{\mathrm{tr}(\mathbf{M}_l^s\mathbf{N}_l)\} \approx 0$ is true for any $s < m$, we show that $E_{\mathbf{g}_l}\{\mathrm{tr}(\mathbf{M}_l^m\mathbf{N}_l) \approx 0$ holds.

For $m = 1$: We have that

$$E_{\mathbf{g}_l}\{\mathrm{tr}(\mathbf{M}_l\mathbf{N}_l)\} = \mathrm{tr}(\bar{\mathbf{N}}_l) - E_{\mathbf{g}_l}\{\mathrm{tr}(\mathbf{L}_l\mathbf{N}_l)\}.$$

Because $g_{l,i}$s are independently and identically distributed random variables with zero mean and finite variance, for given coefficients $\xi_{i,j}$, we have

$$\sum_{i=1}^{n}\sum_{j=1}^{n}\xi_{i,j}E_{\mathbf{g}_l}(g_{l,i}g_{l,j}) = \sum_{i=1}^{n}\sum_{j=1}^{n}\sigma_l^2\xi_{i,j}1_{\{i=j\}} = \sigma_l^2\sum_{i=1}^{n}\xi_{i,i}.$$

Similarly, for given coefficients $\xi_{i,j}$ and $\psi_{i,j}$, we have

$$
\begin{aligned}
&\sum_{i_1=1}^{n}\sum_{j_1=1}^{n}\sum_{i=1}^{n}\sum_{j=1}^{n}\xi_{i_1,j_1}\psi_{i,j}E_{\mathbf{g}_l}(g_{l,i_1}g_{l,j_1}g_{l,i}g_{l,j})\\
&=\sum_{i_1=1}^{n}\sum_{j_1=1}^{n}\sum_{i=1}^{n}\sum_{j=1}^{n}\xi_{i_1,j_1}\psi_{i,j}\{\mu_{l,4}1_{\{i_1=j_1=i=j\}}+\sigma_l^4(1_{\{i_1=j_1\neq i=j\}}+1_{\{i_1=i\neq j_1=j\}}+1_{\{i_1=j\neq j_1=i\}})\}\\
&=\mu_{l,4}\sum_{i=1}^{n}\xi_{i,i}\psi_{i,i}+\sigma_l^4\sum_{i=1}^{n}\sum_{j=1,i\neq i_1}^{n}(\xi_{i_1,i_1}\psi_{i,i}+\xi_{i_1,i}\psi_{i_1,i}+\xi_{i_1,i}\psi_{i,i_1}). \qquad \text{(S8)}
\end{aligned}
$$

The second term is expressed as

$$
\begin{aligned}
&E_{\mathbf{g}_l}\{\mathrm{tr}(\mathbf{L}_l\mathbf{N}_l)\}\\
&=\sum_{a=1}^{p}\sum_{b=1}^{p}E_{\mathbf{g}_l}\left(\sum_{i_1=1}^{n}\sum_{j_1=1}^{n}\sum_{i=1}^{n}\sum_{j=1}^{n}g_{l,i_1}g_{l,j_1}g_{l,i}g_{l,j}G^*_{l,ab,i_1j_1}F^*_{l,ab,ij}\right)\\
&\approx\sum_{a=1}^{p}\sum_{b=1}^{p}E_{\mathbf{g}_l}\Bigg(\sum_{i_1=j_1\neq i=j}g_{l,i_1}g_{l,j_1}g_{l,i}g_{l,j}G^*_{l,ab,i_1j_1}F^*_{l,ab,ij}\\
&\quad+\sum_{i_1=i\neq j_1=j}g_{l,i_1}g_{l,j_1}g_{l,i}g_{l,j}G^*_{l,ab,i_1j_1}F^*_{l,ab,ij}+\sum_{i_1=j\neq j_1=i}g_{l,i_1}g_{l,j_1}g_{l,i}g_{l,j}G^*_{l,ab,i_1j_1}F^*_{l,ab,ij}\Bigg)\\
&\approx\sigma_l^4\sum_{a=1}^{p}\sum_{b=1}^{p}\left(\sum_{i=1}^{n}G^*_{l,ab,ii}\sum_{i=1}^{n}F^*_{l,ab,ii}+\sum_{i=1}^{n}\sum_{j=1}^{n}G^*_{l,ab,ij}F^*_{l,ab,ij}+\sum_{i=1}^{n}\sum_{j=1}^{n}G^*_{l,ab,ij}F^*_{l,ab,ji}\right)\\
&\approx\sigma_l^4\sum_{a=1}^{p}\sum_{b=1}^{p}\sum_{i=1}^{n}G^*_{l,ab,ii}\sum_{i=1}^{n}F^*_{l,ab,ii}\\
&=\sum_{a=1}^{p}\sum_{b=1}^{p}\bar{L}_{l,ab}\bar{N}_{l,ab}\\
&=\mathrm{tr}(\bar{\mathbf{L}}_l\bar{\mathbf{N}}_l)\\
&=\mathrm{tr}(\bar{\mathbf{A}}_l^{-1/2}\bar{\mathbf{A}}_l\bar{\mathbf{A}}_l^{-1/2}\bar{\mathbf{A}}_l^{-1/2}\bar{\mathbf{B}}_l\bar{\mathbf{A}}_l^{-1/2})\\
&=\mathrm{tr}(\bar{\mathbf{A}}_l^{-1/2}\bar{\mathbf{B}}_l\bar{\mathbf{A}}_l^{-1/2})\\
&=\mathrm{tr}(\bar{\mathbf{N}}_l).
\end{aligned}
$$

In the above, the approximations in the second and third line is due to (S8) with $\xi_{i_1,j_1}=G^*_{l,ab,i_1j_1}$ and $\psi_{i,j}=F^*_{l,ab,ij}$, $\mu_{l,4}<\infty$ and

$$
\sum_{i=1}^{n}G^*_{l,ab,ii}F^*_{l,cd,ii}=O(n^{-1}), \qquad \text{(S9)}
$$

for any a, b, c, d. (S9) is the special case of (S15) when $s = 1$ given in the following subsection. The approximation in the fourth line is due to

$$\sum_{i=1}^{n} \sum_{j=1}^{n} G^*_{l,ab,ij} F^*_{l,cd,ij} = O(n^{-1}), \tag{S10}$$

for any a, b, c, d, which is shown in the following subsection. Therefore,

$$E_{\mathbf{g}_l}\{\mathrm{tr}(\mathbf{M}_l \mathbf{N}_l)\} \approx \mathrm{tr}(\bar{\mathbf{N}}_l) - \mathrm{tr}(\bar{\mathbf{N}}_l) = 0.$$

For $m = 2$: We have that

$$\begin{aligned} E_{\mathbf{g}_l}\{\mathrm{tr}(\mathbf{M}_l^2 \mathbf{N}_l)\} &= E_{\mathbf{g}_l}[\mathrm{tr}\{(\mathbf{I} - \mathbf{L}_l)^2 \mathbf{N}_l\}] = E_{\mathbf{g}_l}[\mathrm{tr}\{(\mathbf{I} - 2\mathbf{L}_l + \mathbf{L}_l^2)\mathbf{N}_l\}] \\ &= \mathrm{tr}(\bar{\mathbf{N}}_l) - 2E_{\mathbf{g}_l}\{\mathrm{tr}(\mathbf{L}_l \mathbf{N}_l)\} + E_{\mathbf{g}_l}\{\mathrm{tr}(\mathbf{L}_l^2 \mathbf{N}_l)\} \\ &\approx -\mathrm{tr}(\bar{\mathbf{N}}_l) + E_{\mathbf{g}_l}\{\mathrm{tr}(\mathbf{L}_l^2 \mathbf{N}_l)\}, \end{aligned}$$

where we used the previous result $E_{\mathbf{g}_l}\{\mathrm{tr}(\mathbf{L}_l \mathbf{N}_l)\} \approx \mathrm{tr}(\bar{\mathbf{N}}_l)$.

Let $\mathcal{F}_{2,m,n}$ be the set of all partitions in which any pairing of two indexes is equal among $2m + 2$ indexes $(i_1, j_1, i_2, j_2, \ldots, i_m, j_m, i, j) \in \{1, 2, \ldots, n\}^{2(m+1)}$ but different pairs are distinct, which is equivalent to making $m + 1$ unordered subset of 2 elements from $2m + 2$ elements. For example,

$$\begin{aligned} &\mathcal{F}_{2,1,n} \\ &= \{(i_1, j_1, i, j) \in \{1, 2, \ldots, n\}^4 : \{i_1 = j_1 \neq i = j\} \cup \{i_1 = i \neq j_1 = j\} \cup \{i_1 = j \neq j_1 = i\}\}, \end{aligned}$$

which corresponds to the index set appearing in summation in the second line of (S8),

and

$$
\begin{aligned}
&\mathcal{F}_{2,2,n} \\
&= \{(i_1, j_1, i_2, j_2, i, j) \in \{1, 2, \ldots, n\}^6 : \\
&\{i_1 = j_1 \neq i_2 = j_2 \neq i = j\} \cup \{i_1 = j_1 \neq i_2 = i \neq j_2 = j\} \cup \{i_1 = j_1 \neq i_2 = j \neq j_2 = i\} \cup \\
&\{i_1 = j \neq i_2 = j_2 \neq i = j_1\} \cup \{i_1 = j \neq i_2 = i \neq j_2 = j_1\} \cup \{i_1 = j \neq i_2 = j_1 \neq j_2 = i\} \cup \\
&\{i_1 = j_2 \neq i_2 = j_1 \neq i = j\} \cup \{i_1 = j_2 \neq i_2 = i \neq j_1 = j\} \cup \{i_1 = j_2 \neq i_2 = j \neq j_1 = i\} \cup \\
&\{i_1 = i \neq i_2 = j_2 \neq j_1 = j\} \cup \{i_1 = i \neq i_2 = j_1 \neq j_2 = j\} \cup \{i_1 = i \neq i_2 = j \neq j_2 = j_1\} \cup \\
&\{i_1 = i_2 \neq i = j_2 \neq j_1 = j\} \cup \{i_1 = i_2 \neq i = j_1 \neq j_2 = j\} \cup \{i_1 = i_2 \neq i = j \neq j_2 = j_1\}\}.
\end{aligned} \tag{S11}
$$

Analogous to (S8), for given coefficients $\xi_{i,j}$, $\psi_{i,j}$ and $\phi_{i,j}$, we have

$$
\begin{aligned}
&\sum_{i_1=1}^{n}\sum_{j_1=1}^{n}\sum_{i_2=1}^{n}\sum_{j_2=1}^{n}\sum_{i=1}^{n}\sum_{j=1}^{n} \xi_{i_1,j_1}\psi_{i_2,j_2}\phi_{i,j} E_{\mathbf{g}_l}(g_{l,i_1}g_{l,j_1}g_{l,i_2}g_{l,j_2}g_{l,i}g_{l,j}) \\
&= \sum_{i_1=1}^{n}\sum_{j_1=1}^{n}\sum_{i_2=1}^{n}\sum_{j_2=1}^{n}\sum_{i=1}^{n}\sum_{j=1}^{n} \xi_{i_1,j_1}\psi_{i_2,j_2}\phi_{i,j}\{\mu_{l,6}1_{\{i_1=j_1=i_2=j_2=i=j\}} \\
&+ \sigma_l^2\mu_{l,4}(1_{\{i_1=j_1\neq i_2=j_2=i=j\}} + 1_{\{i_1=j_2\neq i_2=j=j_1=i\}} + 1_{\{i_1=j\neq i_2=j_2=i=j_1\}} + 1_{\{i_1=i_2\neq j_1=j_2=i=j\}} \\
&\quad + 1_{\{i_1=i\neq i_2=j_2=i=j_1\}} + 1_{\{i_2=i\neq i_1=j_1=j_2=j\}} + 1_{\{i_2=j_1\neq i_1=j_2=i=j\}} + 1_{\{i_2=j_2\neq i_1=j=j_1=i\}} \\
&\quad + 1_{\{i_2=j\neq i_1=j_2=i=j_1\}} + 1_{\{i=j_1\neq i_2=j_2=i_1=j\}} + 1_{\{i=j_2\neq i_2=j_1=i_1=j\}} + 1_{\{i=j\neq i_2=j_1=i_1=j_2\}} \\
&\quad + 1_{\{j_1=j_2\neq i_2=i_1=i=j\}} + 1_{\{j_1=j\neq i_2=i_1=i=j_2\}} + 1_{\{j_2=j\neq i_2=i_1=i=j_1\}}) \\
&+ \sigma_l^6(1_{\{i_1=j_1\neq i_2=j_2\neq i=j\}} + 1_{\{i_1=j_1\neq i_2=i\neq j_2=j\}} + 1_{\{i_1=j_1\neq i_2=j\neq j_2=i\}} \\
&\quad + 1_{\{i_1=j\neq i_2=j_2\neq i=j_1\}} + 1_{\{i_1=j\neq i_2=i\neq j_2=j_1\}} + 1_{\{i_1=j\neq i_2=j_1\neq j_2=i\}} \\
&\quad + 1_{\{i_1=j_2\neq i_2=j_1\neq i=j\}} + 1_{\{i_1=j_2\neq i_2=i\neq j_1=j\}} + 1_{\{i_1=j_2\neq i_2=j\neq j_1=i\}} \\
&\quad + 1_{\{i_1=i\neq i_2=j_2\neq j_1=j\}} + 1_{\{i_1=i\neq i_2=j_1\neq j_2=j\}} + 1_{\{i_1=i\neq i_2=j\neq j_2=j_1\}} \\
&\quad + 1_{\{i_1=i_2\neq i=j_2\neq j_1=j\}} + 1_{\{i_1=i_2\neq i=j_1\neq j_2=j\}} + 1_{\{i_1=i_2\neq i=j\neq j_2=j_1\}})\}.
\end{aligned} \tag{S12}
$$

Using (S12) and (S11), the second term is expressed as

$$
\begin{aligned}
&E_{\mathbf{g}_l}\{\mathrm{tr}(\mathbf{L}_l^2\mathbf{N}_l)\}\\
&=\sum_{a=1}^{p}\sum_{b=1}^{p}\sum_{c=1}^{p}E_{\mathbf{g}_l}\left(\sum_{i_1=1}^{n}\sum_{j_1=1}^{n}\sum_{i_2=1}^{n}\sum_{j_2=1}^{n}\sum_{i=1}^{n}\sum_{j=1}^{n}g_{l,i_1}g_{l,j_1}g_{l,i_2}g_{l,j_2}g_{l,i}g_{l,j}G^*_{l,ab,i_1j_1}G^*_{l,bc,i_2j_2}F^*_{l,ca,ij}\right)\\
&\approx\sum_{a=1}^{p}\sum_{b=1}^{p}\sum_{c=1}^{p}E_{\mathbf{g}_l}\left(\sum_{(i_1,j_1,i_2,j_2,i,j)\in\mathcal{F}_{2,2,n}}g_{l,i_1}g_{l,j_1}g_{l,i_2}g_{l,j_2}g_{l,i}g_{l,j}G^*_{l,ab,i_1j_1}G^*_{l,bc,i_2j_2}F^*_{l,ca,ij}\right)\\
&\approx\sigma_l^6\sum_{a=1}^{p}\sum_{b=1}^{p}\sum_{c=1}^{p}\sum_{i_1=1}^{n}\sum_{i_2=1}^{n}\sum_{i=1}^{n}G^*_{l,ab,i_1i_1}G^*_{l,bc,i_2i_2}F^*_{l,ca,ii}\\
&=\mathrm{tr}(\bar{\mathbf{L}}_l^2\bar{\mathbf{N}}_l)\\
&=\mathrm{tr}(\bar{\mathbf{A}}_l^{-1/2}\bar{\mathbf{A}}_l\bar{\mathbf{A}}_l^{-1/2}\bar{\mathbf{A}}_l^{-1/2}\bar{\mathbf{A}}_l\bar{\mathbf{A}}_l^{-1/2}\bar{\mathbf{N}}_l)\\
&=\mathrm{tr}(\bar{\mathbf{L}}_l\bar{\mathbf{N}}_l)\\
&=\mathrm{tr}(\bar{\mathbf{N}}_l).
\end{aligned}
$$

For the approximation in the second line, we used (S8), (S9) and (S15) when $s=2$, i.e.

$$\sum_{i=1}^{n}G^*_{l,ab,ii}G^*_{l,bc,ii}F^*_{l,ca,ii}=O(n^{-2}), \tag{S13}$$

combined with $\mu_{l,6}<\infty$. Also, in the third line, we used (S10) and

$$\sum_{i=1}^{n}\sum_{j=1}^{n}G^*_{l,ab,ij}G^*_{l,cd,ij}=O(n^{-1}), \tag{S14}$$

which is shown in the following subsection, making the summations over the constraints in $\mathcal{F}_{2,2,n}$ being of $O(n^{-1})$ except for the set $\{(i_1,j_1,i_2,j_2,i,j): i_1=j_1\neq i_2=j_2\neq i=j\}$. Therefore,

$$E_{\mathbf{g}_l}\{\mathrm{tr}(\mathbf{M}_l^2\mathbf{N}_l)\}\approx-\mathrm{tr}(\bar{\mathbf{N}}_l)+\mathrm{tr}(\bar{\mathbf{N}}_l)=0.$$

For general m: For induction, assume that

$$E_{\mathbf{g}_l}\{\mathrm{tr}(\mathbf{L}_l^s\mathbf{N}_l)\}\approx\mathrm{tr}(\bar{\mathbf{N}}_l)$$

is true for any $s < m$. Then, by the above induction assumption,

$$\begin{aligned} E_{\mathbf{g}_l}\{\mathrm{tr}(\mathbf{M}_l^m \mathbf{N}_l)\} &= E_{\mathbf{g}_l}[\mathrm{tr}\{(\mathbf{I} - \mathbf{L}_l)^m \mathbf{N}_l\}] \\ &= E_{\mathbf{g}_l}\left[\mathrm{tr}\left\{\sum_{s=0}^{m}(-1)^s \mathbf{L}_l^s \mathbf{N}_l\right\}\right] \\ &= \sum_{s=0}^{m-1}(-1)^s E_{\mathbf{g}_l}\{\mathrm{tr}(\mathbf{L}_l^s \mathbf{N}_l)\} + (-1)^m E_{\mathbf{g}_l}\{\mathrm{tr}(\mathbf{L}_l^m \mathbf{N}_l)\} \\ &\approx \sum_{s=0}^{m-1}(-1)^s \mathrm{tr}(\bar{\mathbf{N}}_l) + (-1)^m E_{\mathbf{g}_l}\{\mathrm{tr}(\mathbf{L}_l^m \mathbf{N}_l)\}. \end{aligned}$$

Then, by letting $\mathcal{P} = \{1, \ldots, p\}$ and $\mathcal{N} = \{1, \ldots, n\}$,

$$\begin{aligned} &E_{\mathbf{g}_l}\{\mathrm{tr}(\mathbf{L}_l^m \mathbf{N}_l)\} \\ &= \sum_{(a,a_1,a_2,\ldots,a_m)\in\mathcal{P}^{m+1}} E_{\mathbf{g}_l}\Bigg(\sum_{(i_1,j_1,i_2,j_2,\ldots,i_m,j_m,i,j)\in\mathcal{N}^{2m+2}} g_{l,i_1}g_{l,j_1}g_{l,i_2}g_{l,j_2}\cdots g_{l,i_m}g_{l,j_m}g_{l,i}g_{l,j} \\ &\qquad \times G^*_{l,aa_1,i_1j_1}G^*_{l,a_1a_2,i_2j_2}\cdots G^*_{l,a_{m-1}a_m,i_mj_m}F^*_{l,a_ma,ij}\Bigg) \\ &\approx \sum_{(a,a_1,a_2,\ldots,a_m)\in\mathcal{P}^{m+1}} E_{\mathbf{g}_l}\Bigg(\sum_{(i_1,j_1,i_2,j_2,\ldots,i_m,j_m,i,j)\in\mathcal{F}_{2,m,n}} g_{l,i_1}g_{l,j_1}g_{l,i_2}g_{l,j_2}\cdots g_{l,i_m}g_{l,j_m}g_{l,i}g_{l,j} \\ &\qquad \times G^*_{l,aa_1,i_1j_1}G^*_{l,a_1a_2,i_2j_2}\cdots G^*_{l,a_{m-1}a_m,i_mj_m}F^*_{l,a_ma,ij}\Bigg) \\ &\approx \sum_{(a,a_1,a_2,\ldots,a_m)\in\mathcal{P}^{m+1}} \sigma_l^{2(m+1)} \sum_{(i_1,i_2,\ldots,i_m,i)\in\mathcal{N}^{m+1}} G^*_{l,aa_1,i_1i_1}G^*_{l,a_1a_2,i_2i_2}\cdots G^*_{l,a_{m-1}a_m,i_mi_m}F^*_{l,a_ma,ii} \\ &= \mathrm{tr}(\bar{\mathbf{L}}_l^m \bar{\mathbf{N}}_l) \\ &= \mathrm{tr}(\bar{\mathbf{N}}_l), \end{aligned}$$

in which we used (S10) and (S14) as in the case of $m = 2$. Therefore, for any m, we have that $E_{\mathbf{g}_l}\{\mathrm{tr}(\mathbf{L}_l^m \mathbf{N}_l)\} \approx \mathrm{tr}(\bar{\mathbf{N}}_l)$, and that

$$E_{\mathbf{g}_l}\{\mathrm{tr}(\mathbf{M}_l^m \mathbf{N}_l)\} \approx \left\{\sum_{s=0}^{m-1}(-1)^s + (-1)^m 1\right\}\mathrm{tr}(\bar{\mathbf{N}}_l) = (1-1)^m \mathrm{tr}(\bar{\mathbf{N}}_l) = 0.$$

Finally, it follows from (S3) that

$$E_{\mathbf{g}_l}(t_l) \approx \mathrm{tr}(\bar{\mathbf{N}}_l) = \mathrm{tr}(\bar{\mathbf{A}}_l^{-1}\bar{\mathbf{B}}_l) = \mathrm{tr}(\bar{\mathbf{A}}_{l,(0)}^{-1}\bar{\mathbf{B}}_{l,(0)}),$$

where the last equality is due to (S4) and (S5), and the elements of $\mathbf{A}_{l,(0)}$ and $\mathbf{B}_{l,(0)}$ are defined by

$$(\bar{\mathbf{A}}_{l,(0)})_{ab} = \sum_{i=1}^{n} \widetilde{x}_{ia}\widetilde{x}_{ib}(\mathbf{Q}_{\widetilde{\mathbf{Z}}})_{ii} \quad \text{and} \quad (\bar{\mathbf{B}}_{l,(0)})_{ab} = \sum_{i=1}^{n} \widetilde{x}_{ia}\widetilde{x}_{ib}(\mathbf{Q}_{\widetilde{\mathbf{Z}}}\mathbf{u})_{i}^{2},$$

giving the approximation formula (7) in the main text.

Technical results

For any $s \geq 1$, because $(\mathbf{Q}_{\widetilde{\mathbf{Z}}})_{ii} = 1 - (\mathbf{P}_{\widetilde{\mathbf{Z}}})_{ii} \in [0,1]$ and hence $(\mathbf{Q}_{\widetilde{\mathbf{Z}}})_{ii}^{s} \leq 1$,

$$\begin{aligned}|\sum_{i=1}^{n} G^{*}_{l,a_1b_1,ii}\cdots G^{*}_{l,a_sb_s,ii}\cdot F^{*}_{l,cd,ii}| &= |\sum_{i=1}^{n}\{\widetilde{x}^{*}_{ia_1}\widetilde{x}^{*}_{ib_1}(\mathbf{Q}_{\widetilde{\mathbf{Z}}})_{ii}\}\cdots\{\widetilde{x}^{*}_{ia_s}\widetilde{x}^{*}_{ib_s}(\mathbf{Q}_{\widetilde{\mathbf{Z}}})_{ii}\}\cdot(\widetilde{x}^{*}_{ic}\widetilde{x}^{*}_{id}r_i^2)| \\ &\leq \max_{i,a}|\widetilde{x}^{*}_{ia}|^{2s+2}\sum_{i=1}^{n}(\mathbf{Q}_{\widetilde{\mathbf{Z}}})_{ii}^{s}r_i^2 \\ &\leq \max_{i,a}|\widetilde{x}^{*}_{ia}|^{2s+2}||\mathbf{r}||^2 \\ &= O(n^{-s-1})O(n) = O(n^{-s}). \qquad \text{(S15)}\end{aligned}$$

Derivation of (S10) To see that (S10) holds, letting $v_{iac} = \widetilde{x}^{*}_{ia}\widetilde{x}^{*}_{ic}r_i$, by the Cauchy–Schwarz inequality,

$$\begin{aligned}|\sum_{i=1}^{n}\sum_{j=1}^{n} G^{*}_{ab,ij}F^{*}_{cd,ij}| &= |\sum_{i=1}^{n}\sum_{j=1}^{n}(\widetilde{x}^{*}_{ia}x^{*}_{ic}r_i)(\widetilde{x}^{*}_{jb}\widetilde{x}^{*}_{jd}r_j)(\mathbf{Q}_{\widetilde{\mathbf{Z}}})_{ij}| \\ &= |\mathbf{v}_{ac}^{T}\mathbf{Q}_{\widetilde{\mathbf{Z}}}\mathbf{v}_{bd}| \\ &= |(\mathbf{Q}_{\widetilde{\mathbf{Z}}}\mathbf{v}_{ac})^{T}(\mathbf{Q}_{\widetilde{\mathbf{Z}}}\mathbf{v}_{bd})| \\ &\leq ||\mathbf{Q}_{\widetilde{\mathbf{Z}}}\mathbf{v}_{ac}||||\mathbf{Q}_{\widetilde{\mathbf{Z}}}\mathbf{v}_{bd}||.\end{aligned}$$

Here,

$$\begin{aligned}||\mathbf{Q}_{\widetilde{\mathbf{Z}}}\mathbf{v}_{ac}||^2 \leq ||\mathbf{v}_{ac}||^2 &= \sum_{i=1}^{n}(\widetilde{x}^{*}_{ia}\widetilde{x}^{*}_{ic}r_i)^2 \\ &\leq \max_{i,a}|\widetilde{x}^{*}_{ia}|^4||\mathbf{r}||^2 = \max_{i,a}|\widetilde{x}^{*}_{ia}|^4||\mathbf{Q}_{\widetilde{\mathbf{Z}}}\mathbf{u}||^2 \\ &\leq \max_{i,a}|\widetilde{x}^{*}_{ia}|^4||\mathbf{u}||^2 = O(n^{-2})O(n) = O(n^{-1}).\end{aligned}$$

Thus,

$$\sum_{i=1}^{n}\sum_{j=1}^{n} G^*_{l,ab,ij} F^*_{l,cd,ij} = O(n^{-1})$$

which is (S10).

Derivation of (S14) To see that (S14) holds,

$$\begin{aligned}
|\sum_{i=1}^{n}\sum_{j=1}^{n} G^*_{l,ab,ij} G^*_{l,cd,ij}| &= |\sum_{i=1}^{n}\sum_{j=1}^{n} (\widetilde{x}^*_{ia}\widetilde{x}^*_{ic})(\widetilde{x}^*_{jb}\widetilde{x}^*_{jd})(\mathbf{Q}_{\widetilde{\mathbf{Z}}})^2_{ij}| \\
&\leq \sum_{i=1}^{n}\sum_{j=1}^{n} |(\widetilde{x}^*_{ia}\widetilde{x}^*_{ic})(\widetilde{x}^*_{jb}\widetilde{x}^*_{jd})|(\mathbf{Q}_{\widetilde{\mathbf{Z}}})^2_{ij} \\
&\leq \max_{i,a} |\widetilde{x}^*_{ia}|^4 \sum_{i=1}^{n}\sum_{j=1}^{n} (\mathbf{Q}_{\widetilde{\mathbf{Z}}})^2_{ij} = \max_{i,a} |\widetilde{x}^*_{ia}|^4 \mathrm{tr}(\mathbf{Q}^2_{\widetilde{\mathbf{Z}}}) \\
&= \max_{i,a} |\widetilde{x}^*_{ia}|^4 \mathrm{tr}(\mathbf{Q}_{\widetilde{\mathbf{Z}}}) \leq O(n^{-2})O(n) = O(n^{-1}).
\end{aligned}$$

Consequently,

$$\sum_{i=1}^{n}\sum_{j=1}^{n} G^*_{l,ab,ij} G^*_{l,cd,ij} = O(n^{-1})$$

which is (S14).

l_{approx} is close to one under correct null model

Consider the score statistic t_l under the loglikelihood function $\ell = \ell(\eta_1, \ldots, \eta_n)$ and $u_i = (\partial/\partial\eta_i)\ell/\omega_i^{1/2}$ with $\omega_i = -(\partial^2/\partial^2\eta_i)\ell$. If the model is correct and n is large, by the Bartlett identity, $E[\{(\partial/\partial\eta_i)\ell\}\{(\partial/\partial\eta_{i'})\ell\}] = -E\{(\partial^2/\partial^2\eta_i)\ell\}1_{i=i'} = \omega_i 1_{i=i'}$, then, $\sum_{i=1}^n \widetilde{\mathbf{x}}_i\widetilde{\mathbf{x}}_i^T(\mathbf{Q}_{\widetilde{\mathbf{Z}}}\mathbf{u})^2_i \approx \sum_{i=1}^n \widetilde{\mathbf{x}}_i\widetilde{\mathbf{x}}_i^T\{\mathbf{Q}_{\widetilde{\mathbf{Z}}}E(\mathbf{u}\mathbf{u}^T)\mathbf{Q}_{\widetilde{\mathbf{Z}}}\}_{ii} = \sum_{i=1}^n \widetilde{\mathbf{x}}_i\widetilde{\mathbf{x}}_i^T(\mathbf{Q}_{\widetilde{\mathbf{Z}}}\mathbf{I}\mathbf{Q}_{\widetilde{\mathbf{Z}}})_{ii} = \sum_{i=1}^n \widetilde{\mathbf{x}}_i\widetilde{\mathbf{x}}_i^T(\mathbf{Q}_{\widetilde{\mathbf{Z}}})_{ii}$. Hence, t_{approx} approximates p, and l_{approx} is close to one if the model is correct.

Marginal association test

If $\mathbf{x}_i = 1$ for all i and $p = 1$, the test reduces to the marginal association test. Then, $t_{approx} = l_{approx} = \mathrm{tr}[\{\sum_{i=1}^n(\mathbf{Q}_{\widetilde{\mathbf{Z}}})_{ii}\}^{-1}\sum_{i=1}^n(\mathbf{Q}_{\widetilde{\mathbf{Z}}}\mathbf{u})^2_i] =$ $\mathrm{tr}[\mathrm{tr}(\mathbf{Q}_{\widetilde{\mathbf{Z}}})\}^{-1}\{\sum_{i=1}^n(\mathbf{Q}_{\widetilde{\mathbf{Z}}}\mathbf{u})^2_i\}] = ||\mathbf{Q}_{\widetilde{\mathbf{Z}}}\mathbf{u}||^2/(n-q)$. For Gaussian linear model, $l_{approx} = T_{approx} = \{||\mathbf{Q}_{\widetilde{\mathbf{Z}}}\mathbf{u}||^2/(n-q)\}/[\{||\mathbf{Q}_{\widetilde{\mathbf{Z}}}\mathbf{y}||^2 - ||\mathbf{Q}_{\widetilde{\mathbf{Z}}}\mathbf{u}||^2/(n-q)\}/n] =$

$\{||\mathbf{Q}_{\tilde{\mathbf{Z}}}\mathbf{y}||^2/(n-q)/[\{||\mathbf{Q}_{\tilde{\mathbf{Z}}}\mathbf{y}||^2 - ||\mathbf{Q}_{\tilde{\mathbf{Z}}}\mathbf{y}||^2/(n-q)\}/n]\} \approx 1$. Thus, the mean of the test statistics is approximately one irrespective of what null model is used.

Influence of centering $g_{l,i}$ and coding of $\mathbf{x}_i$

Our model is $\mathbf{w}_{l,i}\beta_l + \mathbf{z}_i\gamma_l$ where $\mathbf{w}_{l,i} = g_{l,i}\mathbf{x}_i$. Recall that $\mathbf{z}_i = (\mathbf{z}_{(1:p),i}, \mathbf{z}_{(1+p):q,i}) = (\mathbf{x}_i, \mathbf{z}_{(1+p):q,i})$. Then, for any constant c, $\mathbf{w}_{l,i}\beta_l + \mathbf{z}_i\gamma_l = g_{l,i}\mathbf{x}_i\beta_l + \mathbf{x}_i\gamma_{l,1:p} + \mathbf{z}_{(1+p):q,i}\gamma_{l,(1+p):q} = (g_{l,i} - c)\mathbf{x}_i\beta_l + \mathbf{x}_i(c\beta_l + \gamma_{l,1:p}) + \mathbf{z}_{(1+p):q,i}\gamma_{l,(1+p):q}$, which implies that subtracting c from $g_{l,i}$ does not alter the regression coefficients β_l. Consequently, the score test for testing $\beta_l = \mathbf{0}$ does not change if $g_{l,i}$ is centered. The influence is absorbed into the regression coefficients of $\mathbf{x}_i$.

Next, we consider the influence of coding of $\mathbf{x}_i$. For any invertible matrix $\mathbf{T}$ of size $p \times p$, denoting its inverse by $\mathbf{T}^{-1}$, we have that $\mathbf{w}_{l,i}\beta_l + \mathbf{z}_i\gamma_l = g_{l,i}\mathbf{x}_i\beta_l + \mathbf{x}_i\gamma_{l,1:p} + \mathbf{z}_{(1+p):q,i}\gamma_{l,(1+p):q} = g_{l,i}(\mathbf{x}_i\mathbf{T})(\mathbf{T}^{-1}\beta_l) + (\mathbf{x}_i\mathbf{T})(\mathbf{T}^{-1}\gamma_{l,1:p}) + \mathbf{z}_{(1+p):q,i}\gamma_{l,(1+p):q}$. Then, $\beta_l = \mathbf{0}$ is equivalent to $\mathbf{T}^{-1}\beta_l = \mathbf{0}$ since $\mathbf{T}$ is invertible. Therefore, for any invertible matrix $\mathbf{T}$ of size $p \times p$, replacing environment variables $\mathbf{x}_i$ by $\mathbf{x}_i\mathbf{T}$ does not alter the hypothesis test.

Technical details of simulation studies

Here, we describe the technical details of simulation studies in the main text.

Simulation scheme common to all scenarios

Phenotypic value y_i $(i = 1, \ldots, n)$ is modeled by the regression model eq. (1) in "The approximation formula" section of the main text or eq. (S1), with a given environment variable $\mathbf{x}_i$, q covariates $\mathbf{z}_i = (z_{1,i}, \ldots, z_{q,i})^T$ and each variant $g_{l,i}$ $(l = 1, \ldots, L)$. We set $\mathbf{x}_i = (1, z_{1,i})$ (i.e. the first covariate is the environment variable) and used additive coding for $g_{l,i}$ for each l.

For genotype data, we simulated n samples with $L = 2000$ variants consisting of 20 independent blocks, each of which had 100 SNPs made by summing two 100-dimensional binary (0 or 1) random variables so that each element takes a value in

$\{0, 1, 2\}$ (i.e. minor allele count). The 100-dimensional binary random variables were created by thresholding correlated normal random variables using `bindata` package for R with a given correlation matrix whose diagonal and off-diagonal elements are one and ρ, respectively. That is, the correlation between any pair of genetic variants is always the same value of ρ. Minor allele frequency at each variant was generated from a pre-specified distribution (see below).

Given three effect size parameters b_G, b_{GE} and b_Z as input, we generated phenotypic value, y_i, from the following model having the transformed conditional mean,

$$\eta_i^* = \tau(g_{1000,i})b_G + \tau(g_{1000,i})z_{1,i}b_{GE} + \sum_{j=1}^{q} z_{j,i}(b_Z/q), \quad \text{(S16)}$$

in which τ denotes a given genotype coding of the causal variant, $g_{1000,i}$, i.e. 1000th genetic variant. We considered quantitative and binary phenotypes. For quantitative phenotype, Gaussian linear regression model $\eta_i^* + \epsilon_i$ was considered, where $\epsilon_i \sim N(0, 1)$. For binary phenotype, logistic regression model with success probability $1/(1 + e^{-\eta_i^*})$ was considered.

The simulations are carried out for two sample sizes, $n = 1000$ and 10000, and for three effect size scenarios, $b_G = 0, b_Z = 0, b_{GE} = 0$, $b_G = 0, b_Z = 1, b_{GE} = 0$, and $b_G = 0, b_Z = 0, b_{GE} = 1$. For the scenarios where genotypic effect exists, i.e. when $(b_G, b_Z, b_{GE}) = (0, 1, 0)$ and $(0, 0, 1)$, we considered three genotype codings, additive, recessive, or dominant. We repeated the simulations 200 times to compare l_{approx} with l_{mean}.

In the following, we provide the technical details of the simulation scenarios described in Table 1 in the main text.

Baseline scenario

Base. This is the baseline scenario. It is used to make other scenarios by a slight modification. The true model is the linear model in (S1) with $q = 2$ and given (b_G, b_{GE}, b_Z) including one normally distributed covariate variable $z_{2,i}$. Environment variable $z_{1,i}$, covariate variable $z_{2,i}$ and genotypes are independent, where $z_{1,i}$ and $z_{2,i}$ are independent standard normal random variables. Genotypes are in linkage equilibrium ($\rho = 0$ where ρ is the off-diagonal element of correlation matrix among 100

SNPs in each of 20 independent blocks) with uniformly distributed minor allele frequencies in $[0.05, 0.5]$. The null model for all tests is correctly specified.

Other scenarios are created by the baseline scenario with modifications described below while other settings are unchanged.

Association among environment, covariate variables and/or genotypes

1a. Covariate is associated with genotypes by generating independent standard normal random variables $z_{1,i}$ (environment variable) and $z^*_{2,i}$, and the covarite variable $z_{2,i}$ is set as $z_{2,i} = z^*_{2,i}/50 + L^{-1}\sum_{l=1}^{L} g_{l,i}$.

1b. Environment variable is associated with genotypes by generating two independent standard normal random variables $z^*_{1,i}$ and $z_{2,i}$ (covariate variable), and the environment variable $z_{1,i}$ is set as $z_{1,i} = z^*_{1,i}/50 + L^{-1}\sum_{l=1}^{L} g_{l,i}$.

1c. Covariate and environment variables are associated with genotypes by generating two independent standard normal random variables $z^*_{1,i}$ and $z^*_{2,i}$, the environment variable $z_{1,i}$ is set as $z_{1,i} = z^*_{1,i}/50 + L^{-1}\sum_{l=1}^{L} g_{l,i}$, and the covariate variable $z_{2,i}$ is set as $z_{2,i} = z^*_{2,i}/50 + L^{-1}\sum_{l=1}^{L} g_{l,i}$.

1d. Covariate is associated with environment variable by generating environment variable $z_{1,i}$ and covariate variable $z_{2,i}$ from a bivariate normal distribution with mean zero, variance one and correlation 0.5.

Misspecified null model

2a. Covariate associated with genotypes is missed. The data is generated in the same way as scenario 1a, but the covariate $z_{1,i}$ is ignored in the null model.

2b. Covariate associated with genotypes and environment variable is missed. The data is generated in the same way as scenario 1c, but the covariate $z_{1,i}$ is ignored in the null model.

2c. Linear null model is incorrectly specified. Given (b_G, b_{GE}, b_Z), data is generated from the quadratic conditional mean model,
$\eta_i^* = \tau(g_{1000,i})b_G + \tau(g_{1000,i})z_{1,i}b_{GE} + \sum_{j=1}^{2} z_{j,i}(b_Z/2) - z_{1,i}^2$ rather than the linear model (S1).

2d. One outlier is involved. It is in the first index taking a value of 99, while the other data is generated from the linear model (S1) for $q = 2$ and given (b_G, b_{GE}, b_Z).

2e. Ten outliers are involved. These are in the first ten indexes taking a value of 99, while the other data is generated from the linear model (S1) for $q = 2$ and given (b_G, b_{GE}, b_Z).

Environment/covariate variable distribution

3a. Environment variable $z_{1,i}$ and five covariates $z_{2,i}, \ldots, z_{6,i}$ are independent standard normal random variables.

3b. Environment variable $z_{1,i}$ and one covariate $z_{2,i}$ are uniformly distributed in $[0, 5]$.

3c. Environment variable $z_{1,i}$ and one covariate $z_{2,i}$ are binary variables from independent Bernoulli distribution with success probability 0.5.

3d. Environment variable $z_{1,i}$ and one covariate $z_{2,i}$ are ordinal variable from independent binomial random variables with success probability 0.5 with number of trials 3.

Genotype distribution

4a. Genotypes are in linkage disequilibrium ($\rho = 0.5$ where ρ is the off-diagonal element of correlation matrix among 100 SNPs in each of 20 independent blocks) with uniformly distributed minor allele frequencies in $[0.05, 0.5]$.

4b. Genotypes are in linkage equilibrium ($\rho = 0$) with minor allele frequencies from Beta(1,10) distribution where values outside of $[0.05, 0.5]$ are truncated at the limit.

4c. Genotypes are in linkage disequilibrium ($\rho = 0.5$) with minor allele frequencies from Beta(1,10) distribution where values outside of $[0.05, 0.5]$ are truncated at the limit.
